# Multiscale Molecular Orientation in Polyacrylonitrile Precursors for Carbon Fibers: Processing Regulation, Characterization Methodologies, and Structure–Property Correlations

**DOI:** 10.3390/polym18192442

**Published:** 2026-10-07

**Authors:** Hailong Zhang, Chengyu Zhan, Ling Quan, Aijun Gao, Lianghua Xu

**Affiliations:** 1 School of Materials Science and Engineering, North China University of Water Resources and Electric Power, Zhengzhou 450045, China; zhanchengyu163@163.com; 2 College of Electrical Engineering, North China University of Water Resources and Electric Power, Zhengzhou 450045, China; quanling@ncwu.edu.cn; 3 National Carbon Fiber Engineering Research Center, Beijing University of Chemical Technology, Beijing 100029, China; gaoaj@buct.edu.cn

**Keywords:** polyacrylonitrile precursor, hierarchical orientation, spinning process, structure–property relationship, characterization technique, carbon fiber

## Abstract

Polyacrylonitrile (PAN) precursor fibers represent the dominant feedstock for manufacturing high-performance carbon fibers, and the hierarchical molecular orientation established during precursor processing fundamentally determines the tensile strength and elastic modulus of the final products. However, the precise regulation of multiscale orientational structures across spinning, hot-drawing, and thermal treatment stages remains a long-standing bottleneck for breaking the performance ceiling of carbon fibers. This review systematically elucidates the formation and evolution mechanisms of oriented structures at four hierarchical levels: molecular chains, crystalline domains, microfibrils, and fiber assemblies. Flow-induced orientation behaviors in three mainstream spinning routes (conventional wet spinning, dry-jet wet spinning, and electrospinning) are comparatively analyzed, followed by a discussion on crystallization-driven orientation during hot stretching and orientation inheritance throughout pre-oxidation and carbonization processes. Quantitative evidence from reported PAN precursor datasets confirms that increasing the orientation factor from 0.7 to 0.9 is associated with a tensile-strength gain from approximately 2.4 to 4.7 GPa and a modulus gain from 230 to 550 GPa in the corresponding carbon fibers, illustrating the magnitude of the orientation-performance relationship within specific material systems. We critically appraise widely used characterization techniques including X-ray scattering, polarized spectroscopy, optical birefringence, and electron microscopy, with particular focus on their spatial resolution, applicable scenarios, and inherent limitations, and propose combined characterization strategies for multiscale orientation analysis. Critical bottlenecks, including cross-scale modeling gaps, dynamic orientation kinetics under multi-field coupling, and industrial scalability limitations, are identified, and future research directions such as in situ synchrotron characterization and machine learning-assisted process optimization are outlined. This review builds a systematic framework linking molecular-level orientation control to macroscopic carbon fiber properties, providing theoretical guidance for the rational design of high-performance PAN precursors.

## 1. Introduction

### 1.1. PAN Fibers

Industrial interest in polyacrylonitrile (PAN) fibers began in the 1930s and reached large-scale production in the 1940s when DuPont introduced polar solvents (dimethylformamide, DMF) for solution spinning [1]. The transition from textile commodity to structural precursor occurred in 1959, when Shindo at the Osaka Technical Research Institute first reported carbon fibers derived from PAN precursors [2]. A decisive advance followed in the 1960s at the UK Royal Aircraft Establishment, where applied tensile stress during pre-oxidation markedly improved mechanical properties and established PAN as a credible precursor for load-bearing carbon fibers. Toray’s commercial launch of T300 in 1971 opened the sporting-goods market, and Boeing’s adoption of T300 for aircraft structures in 1982 cemented PAN-based carbon fibers in aerospace engineering [3].

Beyond conventional solvents such as DMF, dimethyl sulfoxide (DMSO), and aqueous sodium thiocyanate, N-methylmorpholine-N-oxide (NMMO) has recently emerged as a green, recyclable solvent for PAN spinning. NMMO containing about 8–10 wt% water can dissolve PAN at concentrations up to 55 wt%, far exceeding the 12–18 wt% limit of traditional solvents, thereby reducing solvent recovery load and suppressing void formation during phase separation. The route also permits co-spinning of PAN with renewable polymers such as cellulose, although large-scale implementation remains constrained by the high dissolution temperature (~120 °C) and limited long-term spinning stability data [4,5].

Outside the carbon-fiber sector, PAN fibers serve as “synthetic wool” in thermal apparel, as chemically inert filtration media [6], and as biocompatible scaffolds in tissue engineering [7]. However, the dominant driver of PAN research today is its role as the precursor for high-performance carbon fibers (PAN-CFs), which exceed >95% carbon and combine high specific strength, low density, and long-term durability. These properties make PAN-CFs indispensable in aerospace, wind-turbine blades, and automotive lightweighting, while imposing strict demands on structural uniformity during precursor manufacturing [8,9,10].

The global carbon fiber market was valued at approximately USD 5.86 billion in 2026, with PAN-based precursors accounting for over 85% of total production volume [11]. The production chain, from acrylonitrile polymerization to composite fabrication, is characterized by high technical barriers and substantial value addition. Global demand is projected to reach 164,900 tons by 2025 and 266,100 tons by 2030, with a compound annual growth rate near 10.05% [12]. This expansion is driven by defense, aerospace, new-energy vehicles, wind-energy blades, and sporting goods. Because the mechanical performance of the final carbon fiber is largely predetermined during PAN spinning and thermal treatment, this market expansion underscores the need for refined mechanistic understanding and tighter process control over precursor orientational structure.

### 1.2. The Orientation Bottleneck

Among the structural parameters governing PAN-CF performance, molecular orientation, defined as the preferential alignment of chain segments along the fiber axis, exerts the strongest influence on final mechanical properties [13]. Orientational order established during spinning and drawing dictates the crystalline texture of the precursor, which in turn governs the fiber’s response to thermal stabilization and carbonization. Precursors with poor chain alignment exhibit chain slippage and premature fracture under tensile loading, whereas well-aligned chains facilitate graphitic layer formation and deliver superior tensile strength and modulus. Quantitative data illustrate the magnitude of this effect. Across published PAN precursor datasets, raising the WAXD crystalline orientation factor from approximately 0.7 to 0.9 is associated with an increase in carbon-fiber tensile strength from roughly 2.4 to 4.7 GPa [14]. The corresponding tensile modulus rises from approximately 230 to 350 GPa in the as-carbonized state, and can reach 550 GPa after high-temperature graphitization under tension, where further refinement of graphite preferred orientation takes place [15]. These values correspond to industrial-grade dry-jet wet-spun PAN precursors processed with optimized multi-stage hot-drawing protocols. The orientation factor refers to graphite crystalline preferred orientation measured by wide-angle X-ray diffraction, and mechanical properties were obtained from standard tensile testing of continuous carbon fiber tows.

Orientation control remains a bottleneck because PAN structure is inherently hierarchical. An ideal carbon fiber requires coordinated alignment across molecular chains, microfibrils, and fibrils, yet processing conditions optimized for one length scale frequently degrade order at another. First, at the microstructural level, insufficient chain alignment in the precursor nucleates microdefects during carbonization, including grain-boundary dislocations and microvoids, which impair load-transfer efficiency. Second, at the fiber level, radial orientational gradients generate localized stress concentrations under mechanical loading, preventing the material from approaching its theoretical strength. As a consequence, empirical tuning of spinning or drawing parameters, in the absence of a mechanistic framework for orientational inheritance, cannot resolve the strength–modulus trade-off.

### 1.3. Outstanding Scientific and Technical Questions

Several fundamental aspects of PAN orientational order remain poorly understood despite extensive research.

(1) Dynamics under multi-field coupling

During spinning, molecular chains are subjected to simultaneous flow, thermal, and electric fields. Shear and elongational stresses cooperate to drive alignment, but current kinetic models fail to capture segmental relaxation and reorientation at the strain rates encountered in industrial processing. Less still is known about how the conformational state fixed during spinning propagates through the cyclization reactions of pre-oxidation and the condensation reactions of carbonization. The interplay between chemical kinetics, such as cyclization rate, and physical orientational processes, including chain straightening and thermal relaxation, has not been resolved at either the atomic or mesoscopic scale.

(2) Quantitative orientation-property links

Correlations between precursor orientational features, including cross-scale alignment uniformity and defect distribution, and the mechanical properties of derived carbon fibers remain predominantly empirical. The absence of reliable quantitative descriptors hinders predictive design of carbon-fiber grades from precursor specifications.

(3) Processing and characterization limitations

Conventional mechanical drawing and thermal stretching improve orientational order but often induce structural damage and incur high energy costs. Electrospinning produces highly oriented nanofibers but lacks scalability for industrial tow production. On the characterization side, real-time tracking of chain dynamics and defect nucleation during spinning remains challenging. In particular, in situ techniques capable of monitoring orientational evolution concurrently across multiple length scales are unavailable, restricting both mechanistic understanding and process optimization.

### 1.4. Scope and Objectives

This review addresses the formation, evolution, and characterization of orientational structures in PAN fibers through three interconnected objectives. First, it analyzes orientational mechanisms across the molecular, microfibrillar, and fibrillar scales, with emphasis on how spinning parameters and drawing protocols regulate hierarchical alignment. Second, it evaluates quantitative structure–property relationships linking precursor orientational order to the tensile strength and modulus of derived carbon fibers, identifying the metrics with greatest predictive value. Third, it critically assesses existing characterization tools, including X-ray scattering, vibrational spectroscopy, birefringence, and electron microscopy, in terms of spatial resolution and industrial applicability, and discusses how their combined deployment can resolve the multiscale nature of orientation.

By integrating these three perspectives, this review connects orientational science to carbon-fiber engineering. It closes by identifying unresolved questions and actionable research priorities, including in situ synchrotron characterization, multi-field processing strategies, and data-driven modeling, as pathways toward next-generation PAN-based carbon fibers.

## 2. Preparation Process and Orientation Structure Evolution in PAN Fibers

### 2.1. Chemical Structure and Spinning Technologies

#### 2.1.1. Copolymer Composition

Copolymer composition determines a controlling influence on spinning dope rheology, processability, and the mechanical performance of derived carbon fibers. PAN is synthesized via free-radical polymerization of acrylonitrile (AN). During thermal treatment, pendant cyano groups (C≡N) undergo intramolecular cyclization to form ladder-type aromatic structures, which provide the structural basis for carbon-fiber tensile strength.

**Figure 1:**
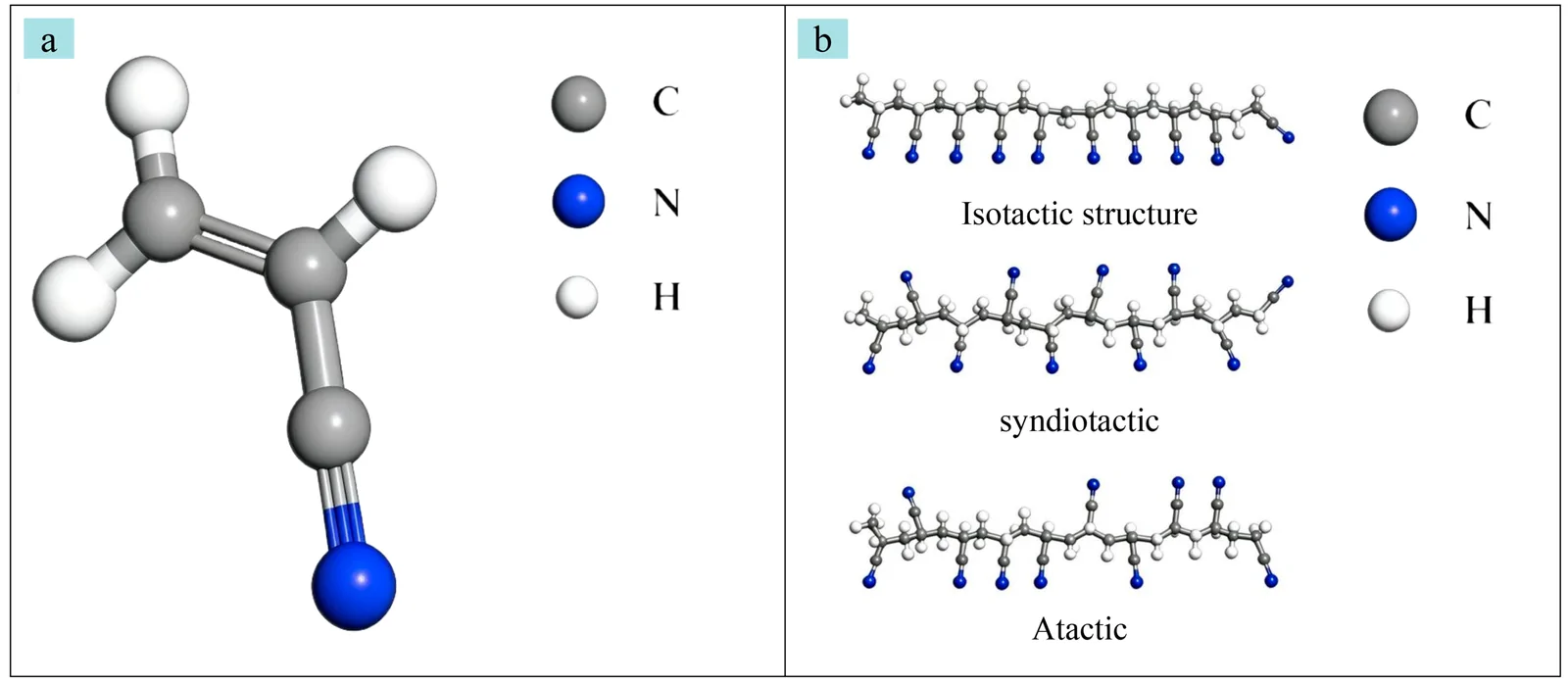
(**a**) Acrylonitrile basic structural unit and (**b**) isotactic, syndiotactic, and atactic configurations of PAN.

The chemical structure of the AN repeat unit is depicted in Figure 1a. PAN homopolymer can exist in three stereoregular configurations: isotactic, syndiotactic, and atactic, as shown in Figure 1b [16]. Homopolymeric PAN, however, presents excessively high dope viscosity and poor spinnability because of strong dipole–dipole interactions between adjacent cyano groups. This limitation is addressed industrially by incorporating comonomers, most commonly itaconic acid [17,18,19] and methyl acrylate [20]. These comonomers enhance copolymer hydrophilicity, melt/solution processability, and thermal stabilization behavior [21,22,23]. A representative optimized molar ratio of AN:MA:IA = 96:3:3 delivers a spinning dope with good spinnability and controlled pre-oxidation kinetics. Carbon fibers produced from this precursor achieve a tensile strength of 3.8 ± 0.4 GPa and an elastic modulus of 257.5 ± 7.5 GPa [24].

#### 2.1.2. Comparison of Spinning Technologies

The spinning route establishes the initial orientational state of PAN fibers, including molecular chain alignment and crystalline order, and thereby governs an upper bound on the mechanical properties of the resulting carbon fibers. Table 1 compares four principal spinning technologies, namely wet spinning, dry-jet wet spinning, dry spinning, and electrospinning, with respect to process characteristics, orientational mechanisms, advantages, and limitations.

### 2.2. Definition and Formation Mechanism of Orientation

Orientational order in PAN fibers describes the preferential alignment of structural entities, including molecular chains, crystalline domains, and lamellar stacks, along the fiber axis. This alignment constitutes an entropy-reducing process driven by external flow, stress, or thermal gradients. The orientational architecture of PAN fibers is hierarchical and multiscale, evolving continuously from spinning through drawing, pre-oxidation, and carbonization. These coupled orientational features collectively determine the mechanical performance of the resulting carbon fibers.

#### 2.2.1. Multiscale Orientation Hierarchy

(1) Molecular chain orientation (0.1–10 nm)

The PAN molecular chain, composed of covalently linked AN repeat units, forms the fundamental structural entity. Under applied stress, chain segments align preferentially along the loading direction. During spinning, shear flow within the spinneret orifice subjects macromolecules to forces that drive the transition from random-coil to extended-chain conformations along the flow direction, with interchain hydrogen bonding stabilizing the nascent oriented state.

Strong dipole–dipole interactions between adjacent -C≡N groups further promote segmental alignment along the stretching axis, forming local paracrystalline domains. Spinning parameters, including dope concentration, temperature, and extrusion rate, regulate the extent of chain orientation. Comonomers such as itaconic acid or methyl acrylate disrupt cyano dipole ordering through steric hindrance and altered hydrogen bonding, introducing localized structural disorder.

Molecular chain orientation serves two coupled functions: it directly governs the mechanical properties of the precursor fiber, and it provides a structural template for crystalline domain formation and alignment during subsequent thermal processing.

(2) Crystalline domain orientation (10–100 nm)

During spinning and drawing, chain folding and stacking drive crystallization. The azimuthal narrowing of the (100) reflection describes the preferential alignment of paracrystalline chain-packing bundles within the fiber. Under thermal drawing, crystal nuclei grow preferentially along pre-oriented chain directions, generating an anisotropic crystalline texture. Chain orientation in the amorphous regions further induces crystalline alignment through hydrogen bonding and dipole–dipole interactions across the crystalline-amorphous interface.

The degree of crystalline orientation correlates directly with the tensile modulus of both precursors and carbon fibers. Optimized thermal drawing, with temperatures maintained at 80–100 °C above the glass transition temperature and precise draw-ratio control, effectively promotes crystalline orientation. This processing window enhances structural order and reinforces the load-bearing capacity of the final carbon fibers.

(3) Lamellar and microfibrillar orientation (100 nm–1 μm)

Lamellar and microfibrillar structures comprise alternating crystalline and amorphous domains aligned along the fiber axis. During fiber formation, shear thinning of the dope induces liquid–liquid phase separation, generating polymer-rich primary fibrils that aggregate into microfibrils through hydrogen bonding and van der Waals forces. Nanoscale porosity forms as solvent and nonsolvent counter-diffuse through the nascent filament; the rate mismatch drives demixing and leaves elongated microvoids along the fiber axis. In the air gap of dry-jet wet spinning and during electrospinning, concurrent evaporation further concentrates the dope and raises viscosity, kinetically preserving oriented chain conformation before solidification.

Axial alignment of microfibrils produces regional orientation gradients within the fiber. Rapid cooling at the fiber surface forms a dense skin layer, whereas the core retains a porous structure with loose molecular packing. Voids elongate along the stretching direction, creating anisotropic void morphologies that impair transverse mechanical strength. Highly oriented molecular chains and crystallites drive the axial alignment of lamellar stacks.

The orientation state of lamellar structures governs internal stress-transfer efficiency under mechanical loading. Well-ordered lamellar architectures enhance load-distribution uniformity by mitigating stress concentration, particularly under high external stress. This hierarchical orientational mechanism ultimately determines the tensile resilience and structural integrity of PAN precursors and their derived carbon fibers.

(4) Fiber bundle and macroscopic orientation (1 μm–1 mm)

At the macroscopic scale, PAN fibers exhibit collective orientational behavior built upon smaller-scale hierarchical features. In industrial manufacturing, fiber assembly alignment is controlled through spinneret arrangement, winding speed, and post-spinning drawing and heat-setting treatments. During fiber bundle consolidation, mechanical drawing drives individual fibers within the bundle into parallel alignment. This macroscopic orientation results from the combined effects of mechanical stretching and inter-fiber frictional and cohesive interactions.

The degree of fiber bundle orientation profoundly influences the anisotropic mechanical behavior of carbon fibers and their composites. Highly oriented bundles exhibit enhanced axial tensile strength and reduced transverse deformability. In high-performance composite fabrication, precise macroscopic orientation control enables tailored anisotropy in stiffness, strength, thermal conductivity, and thermal expansion, properties essential for optimizing structural efficiency in aerospace and energy storage applications. Skin-core morphology, a common structural feature of solution-spun PAN fibers, introduces radial gradients in both chain packing density and orientational order. This heterogeneity persists through thermal treatment and translates into radial variations in mechanical properties, limiting the bulk performance of fiber tows relative to the intrinsic properties of well-aligned surface regions.

#### 2.2.2. Flow-Field-Induced Orientation During Spinning

In PAN fiber spinning, flow fields comprising both shear and elongational components provide the primary driving force for molecular chain orientation. Flow-field engineering enables targeted modulation of initial chain alignment. Each spinning technology employs characteristic flow regimes that yield distinct molecular orientation profiles, which govern the mechanical strength, thermal stability, and dimensional stability of the resulting carbon fibers.

(1) Wet Spinning

In wet spinning, PAN dope is extruded through a spinneret into a coagulation bath, where counter-diffusion of solvent and coagulant induces phase separation and solidification. Flow-induced orientation arises from two concurrent contributions: shear stress inside the spinneret channel and elongational stress in the coagulation bath. Shear deformation during extrusion aligns chain segments along the flow direction, creating short-range ordered domains in the amorphous phase.

Upon entering the coagulation bath, simultaneous solvent extraction and coagulant infiltration cause rapid phase separation, producing the characteristic skin-core gradient morphology of wet-spun fibers. Subsequent elongational flow in the bath applies axial stretching, progressively aligning molecular chains and consolidating the initial oriented structure. The interplay between diffusion-controlled phase separation and flow-induced stretching determines the final hierarchical orientation profile, governing the balance between skin layer densification and core structural evolution.

Higher spinning speeds and lower coagulation bath temperatures promote more pronounced molecular orientation. Elevated shear rates during spinneret passage enhance chain pre-alignment, while reduced bath temperatures slow solvent diffusion kinetics. Solution viscosity, concentration, and solvent type also critically influence chain alignment during solidification [38].

**Figure 2:**
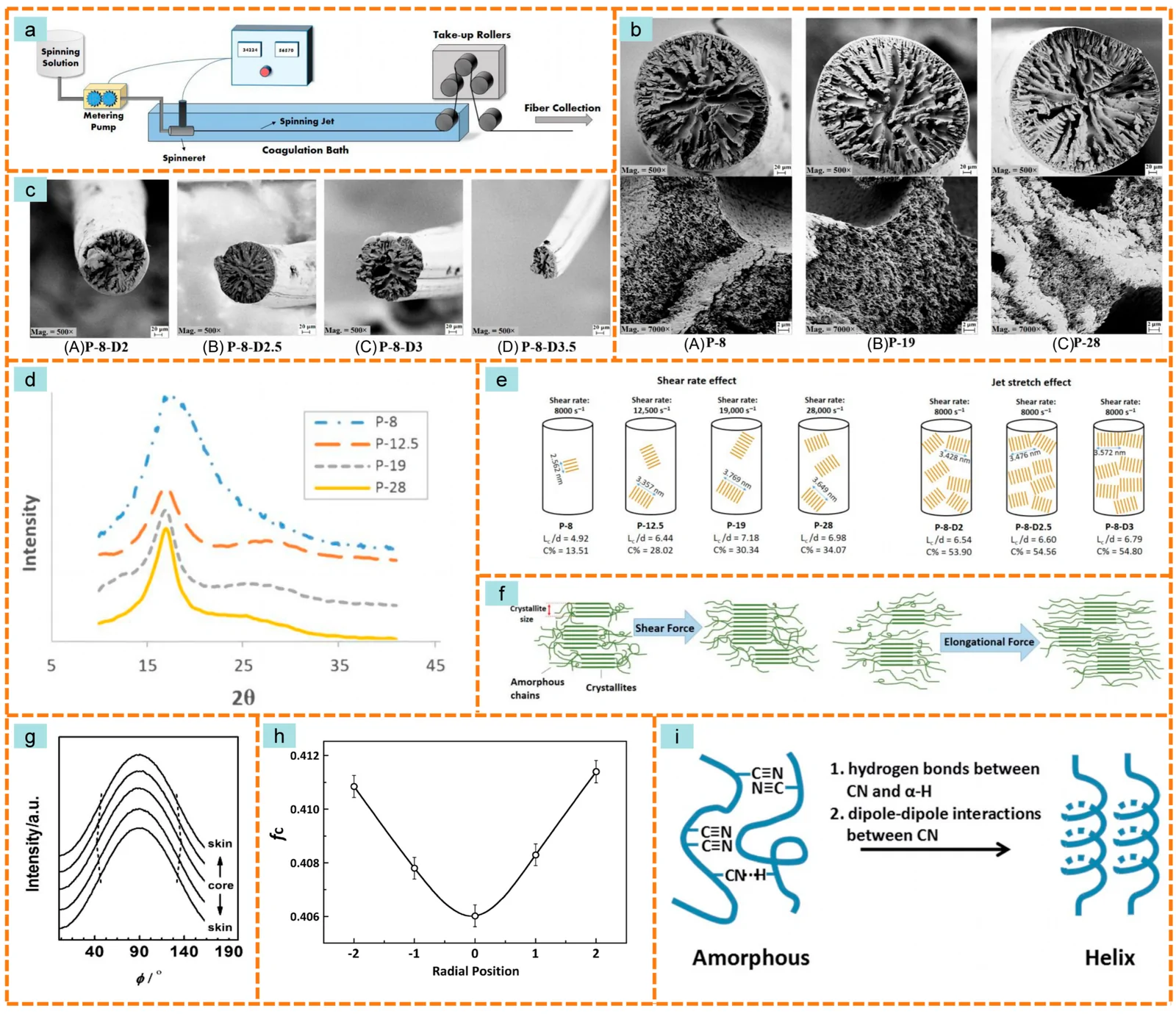
(**a**) Schematic of the wet spinning setup; (**b**) FESEM images of cross-section and internal morphology of PAN fibers at different shear rates; (**c**) FESEM images at different jet stretch ratios; (**d**) XRD patterns at different shear rates; (**e**) schematic of crystalline parameters at different spinning conditions; (**f**) schematic of shear and elongational effects on crystallite size and crystallinity [26]; (**g**) azimuthal distribution of (100) diffraction at different radial positions; (**h**) Hermans orientation factor variation along the radial direction; (**i**) schematic of interactions within PAN molecular chains [27].

Mirbaha et al. [26] investigated the effects of shear and elongational forces on orientation and properties of wet-spun PAN fibers. Figure 2a shows the wet spinning setup. A metering pump controls the volumetric flow rate to generate varying shear forces at the spinneret, while take-up rollers provide tensile stretch in the coagulation bath. Cross-sectional and internal morphologies at different shear rates are presented in Figure 2b. Increasing shear rate raises the jet swell ratio, which disrupts axial chain orientation through radial expansion, producing loose fibril arrangement, disordered pores, and reduced orientational order. Figure 2c shows interfacial morphologies under different jet stretch ratios. The cross-section remains circular at stretch ratios up to 2.5, whereas surface grooves appear at a ratio of 3.5. Elongational force effectively promotes axial chain orientation, maintaining circular cross-sections and compact structures. Excessive stretch ratios, however, induce intensified chain motion that compromises orientation and produces irregular cross-sections. Figure 2d presents XRD patterns reflecting orientational behavior at different shear rates. The crystalline peak intensity increases and the full width at half maximum decreases with shear rate, indicating that shear facilitates axial orientation and enhances crystallinity. At an ultra-high shear rate of 28,000 s^−1^, the crystalline peak intensity slightly decreases, suggesting that excessive shear disrupts orientational order. Figure 2e illustrates crystalline parameters of PAN fibers. Under different shear rates, the crystallite layer index Lc/d increases from 4.92 to 7.18, and crystallinity rises from 13.51% to 34.07%; under different stretch ratios, Lc/d increases from 6.54 to 6.79 and crystallinity from 53.90% to 54.80%. Higher orientational degrees correspond to more crystalline layers and more regular chain arrangement. Figure 2f schematically illustrates the effects of shear and elongational forces on crystallite size and crystallinity. Shear force increases crystallite size, while elongational force stretches and orients amorphous chains, increasing both crystallite size and crystallinity. All experiments used a 20 wt% PAN solution in DMF with aqueous DMF coagulation baths; crystalline orientation and crystallinity were characterized by WAXD.

Zhou et al. [27] examined the radial variation in initial molecular orientation in PAN nascent fibers under shear flow during wet spinning. During extrusion through the spinneret, the shear field provides the primary driving force for initial orientation. Owing to the radial velocity gradient within the spinneret channel, molecular chains in the skin region experience substantially stronger shear forces, leading to rapid disentanglement and pre-alignment of chain segments along the flow direction. In contrast, chains in the core region undergo negligible shear deformation and remain predominantly in random-coil conformations.

Upon entering the coagulation bath, counter-diffusion between solvent and nonsolvent dominates phase separation kinetics and magnifies the radial orientation discrepancy. The skin layer undergoes earlier phase separation and solidification, freezing the pre-oriented structure and allowing sufficient time for relaxation and ordered assembly. The crystal orientation factor thus exhibits a declining gradient from the fiber surface toward the core, as shown in Figure 2g,h. Under the coupled effects of strong shear and rapid solidification, chains in the skin layer stabilize into a more ordered helical conformation with enhanced interchain hydrogen bonding and dipolar interactions, yielding markedly higher packing regularity than the core region, as depicted in Figure 2i.

Reported orientation levels across wet-spinning studies show notable scatter, which arises primarily from three coupled variables: spinneret aspect ratio, coagulation bath temperature, and comonomer formulation. For example, Mirbaha et al. [25] observed a crystallinity rise from 13.51% to 34.07% with increasing shear rate, whereas Zhou et al. [26] reported a more moderate orientation gradient across the fiber radius under comparable shear conditions. This discrepancy is attributed to two factors: first, the higher comonomer content in the formulation used by Zhou et al. introduces steric hindrance that suppresses dipole-driven chain ordering; second, the lower coagulation bath temperature in the work of Mirbaha et al. accelerates phase separation and kinetically traps shear-induced orientation before relaxation can occur.

A more systematic divergence exists between wet-spun and dry-jet wet-spun fibers. Even at equivalent total draw ratios, dry-jet wet-spun fibers consistently show 15–25% higher overall orientation degree and more uniform radial orientation profiles relative to wet-spun counterparts. This advantage stems from the air-gap stage, where uniaxial elongational flow acts on homogeneous polymer solution prior to phase separation, producing more uniform chain pre-alignment than the shear-diffusion coupled environment inside a coagulation bath.

The practical implication is that coagulation bath temperature and solvent concentration must be staged rather than held constant if skin-core gradients are to be minimized while retaining high orientation.

(2) Dry-jet wet spinning

Dry-jet wet spinning is distinguished by an air gap of 0.5–20 mm between the spinneret exit and the coagulation bath, establishing a three-stage orientational control sequence: shear flow, elongational flow, and solidification. Within the air gap, the PAN solution undergoes Barus-effect die swell upon exiting the spinneret, followed by uniaxial elongation driven by spinning pump pressure and take-up force from the coagulation bath. This process generates elongational strain rates of 1–10 s^−1^, during which PAN molecular chains progress from entanglement through extension to eventual slippage. The tensile stress developed in the air gap significantly enhances molecular orientation, and orientation degree has been shown to scale directly with air-gap length [28]. Concurrent solvent evaporation in the air gap increases solution viscosity and prolongs molecular relaxation times, permitting more complete chain alignment during elongation. The subsequent coagulation bath stage follows orientation mechanisms similar to those in conventional wet spinning, but the pre-orientation imparted in the air gap substantially diminishes dependence on coagulation conditions alone. This hierarchical flow design yields fibers with superior structural integrity and more homogeneous orientation profiles relative to traditional wet spinning.

Ambient humidity in the air gap and coagulation bath temperature critically modulate flow-induced orientation. Relative humidity below 40% promotes surface pore formation via premature solidification, whereas humidity maintained at 60–70% yields fibers with smooth morphologies and homogeneous orientation profiles. Elevated coagulation bath temperatures accelerate solvent-coagulant diffusion kinetics; at the optimum of 40 °C, fibers exhibit circular cross-sections and reduced internal porosity. Such structural refinement facilitates orientational inheritance during subsequent pre-oxidation and carbonization. Compared with conventional wet-spun fibers, dry-jet wet-spun PAN fibers display superior core-region orientation and a more radially uniform structural distribution, as verified by birefringence mapping and small-angle X-ray scattering [29]. This improved orientational uniformity corresponds to higher tensile strength and elastic modulus in the derived carbon fibers.

**Figure 3:**
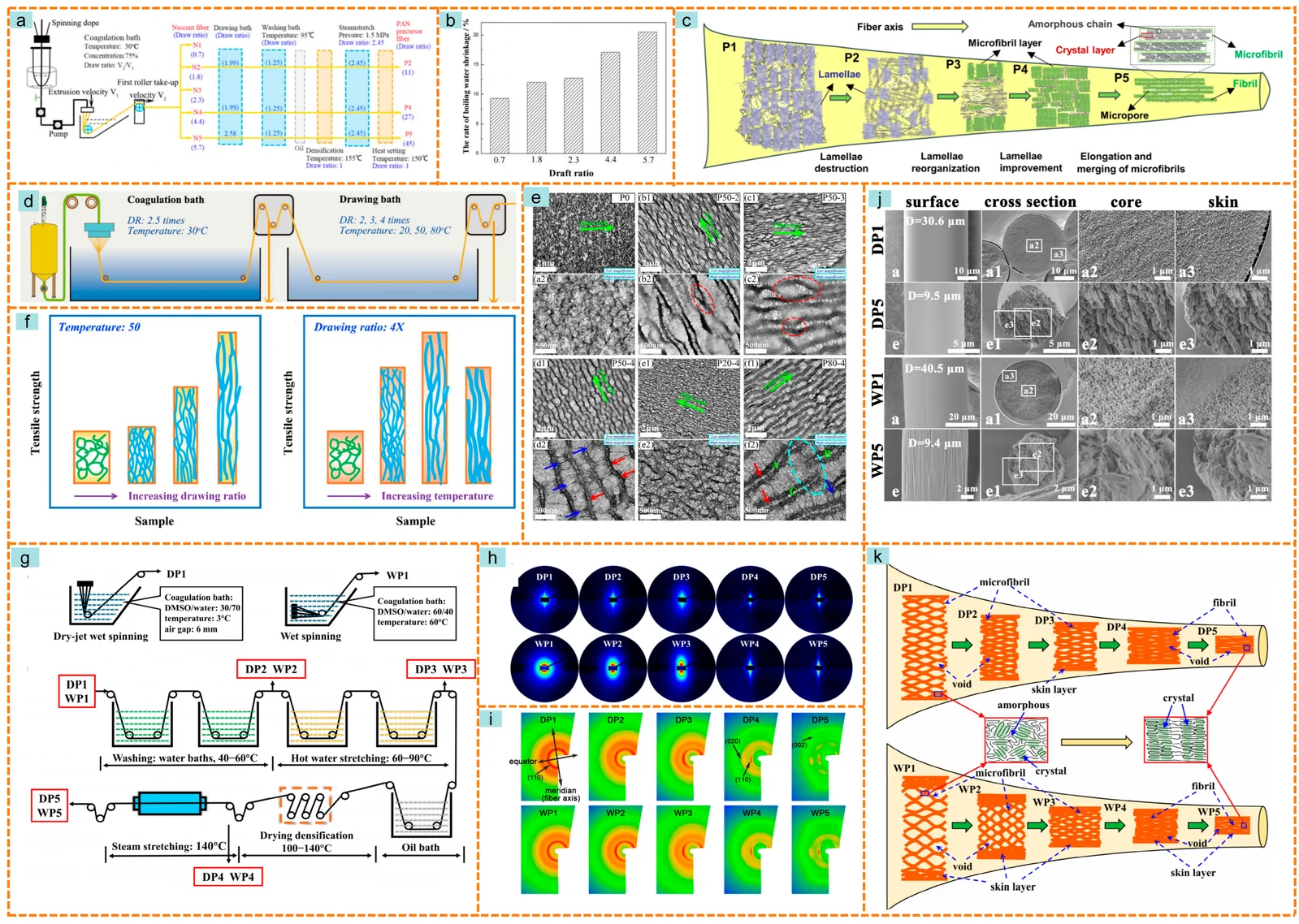
(**a**) Dry-jet wet spinning process schematic; (**b**) boiling water shrinkage of nascent fibers at different draw ratios [28]; (**c**) structural evolution mechanism from P1 to P5 [30]; (**d**) fiber stretching process schematic; (**e**) HRTEM morphology of longitudinal ultrathin section after solution etching; (**f**) microfibril structural evolution during stretching [39]; (**g**) comparison of dry-jet wet spinning and wet spinning; (**h**) SAXS patterns of PAN fibers, (**i**) 2D WAXS patterns; (**j**) SEM images of surface, cross-section, core and skin; (**k**) structural evolution schematic [31].

Gao et al. [28] demonstrated that the high-magnitude axial draw flow field within the coagulation bath constitutes the critical hydrodynamic condition for efficient orientation in dry-jet wet spinning, as shown in Figure 3a. As axial draw flow intensity increases, PAN molecular chains undergo simultaneous slippage, disentanglement, and aligned arrangement during solvent-nonsolvent dual diffusion. Orientational order in the amorphous region increases markedly with draw ratio, as confirmed by boiling water shrinkage measurements, as shown in Figure 3b.

He et al. [30] tracked orientation evolution across the full dry-jet wet spinning route. They resolved distinct flow-field contributions at five stages: coagulation and forming (P1); water washing and boiling drawing (P2–P3); densification (P4); and steam drawing with heat setting (P5). These fields collectively govern chain disentanglement and alignment, lamellar reconstruction, microfibril directional stacking, and axial regularization of microvoids, as illustrated in Figure 3c. The study established that synergistic control of hierarchical flow fields provides the core technical strategy for producing highly oriented PAN precursor fibers.

Gao et al. [39] revealed synergistic regulation of oriented structure by axial draw flow and thermal fields, as shown in Figure 3d. The axial draw flow serves as the dominant field driving orientation development and simultaneously induces preferential alignment of crystalline domains along the fiber axis. The thermal field modulates chain mobility and thus exerts a strong regulatory effect on flow-induced orientation, as depicted in Figure 3e,f. However, excessively high temperatures trigger thermal relaxation of pre-oriented molecular chains, which reduces overall orientation, lowers crystallinity, and deteriorates mechanical properties.

Cao et al. [31] systematically compared flow-field characteristics across the full dry-jet wet spinning and conventional wet spinning routes, elucidating the differentiated regulatory effects of air-gap draw, coagulation shear, and post-drawing flow fields on hierarchical orientational structure, as shown in Figure 3g. In dry-jet wet spinning, the as-spun dope experiences a steady flow field of weak shear and strong draw within the air gap. This initial hydrodynamic condition promotes early disentanglement and axial alignment of PAN chains at the onset of gelation, establishing a homogeneous pre-oriented structure. Upon entering the coagulation bath, the coupled diffusion-shear flow field supported a mild and uniform phase transition. Consequently, orientational evolution in the skin and core layers exhibits high synchronization, yielding as-spun fibers with high orientational order, minimal skin-core heterogeneity, and compact structural integrity. In conventional wet spinning, the spinneret jet directly enters the coagulation bath and encounters a highly unsteady shear-diffusion flow field. Intense solvent-nonsolvent counter-diffusion causes rapid skin-layer solidification while core-layer formation lags significantly. This asynchronous solidification disrupts pre-established molecular orientation through local stress fields, producing a pronounced skin-core heterogeneous structure. Consequently, the overall orientational uniformity and structural integrity of wet-spun as-spun fibers are substantially inferior to those of dry-jet wet-spun fibers, as confirmed in Figure 3h–j.

During post-spinning drawing, the effectiveness of axial draw flow in enhancing orientation differs markedly between the two fiber types. Owing to their homogeneous initial orientation and low defect density, dry-jet wet-spun fibers support cooperative orientation of hierarchical structures, including molecular chains, crystalline domains, and microfibrils, under the draw flow field. This produces regular lamellar alignment along the fiber axis, directional merging of microfibrils, and axial elongation and collapse of microvoids, raising both orientational order and densification concurrently. By contrast, wet-spun fibers are constrained by their initial skin-core heterogeneity. The draw-flow field cannot effectively eliminate orientational defects in the core layer, often causing local orientational inhomogeneity and microfibril fracture. The gain in overall orientational perfection is therefore limited, as illustrated in Figure 3k. The dry-jet wet-spinning process employed a 6 mm air gap and a 3 °C coagulation bath, with orientational structure characterized via a combination of WAXD and SAXS.

The air gap is therefore the critical design element: too short and chain extension is incomplete; too long and solvent evaporation destabilizes the jet before coagulation.

(3) Electrospinning

Electrospinning imparts orientational order to PAN nanofibers through two coupled mechanisms: shear-induced chain alignment within the spinneret needle and electric-field-driven jet elongation, with the choice of collection geometry exerting additional control over the final molecular arrangement. As the solution passes through the needle bore, shear forces induce partial alignment of PAN chains along the flow direction. Application of a high-voltage electrostatic field transforms the pendant droplet into a Taylor cone, from which a charged jet is ejected and undergoes elongation mediated by whipping instability. Jet thinning proceeds under the competing effects of electrostatic driving and viscoelastic resistance, and the resulting elongational flow field produces a draw ratio that scales directly with the degree of molecular orientation.

Collection geometry strongly influences the final orientational texture. Static flat-plate collectors lead to random jet deposition and relatively poor overall alignment. High-speed rotating drums, by contrast, impose tangential velocity gradients that introduce shear, promoting preferential alignment of nanofibers along the drum rotation direction. Such control over fiber alignment is especially relevant for PAN-derived carbon nanofiber precursors, as the inherited anisotropic structure directly affects carbonization kinetics and the degree of graphene layer orientation in the final product [40].

**Figure 4:**
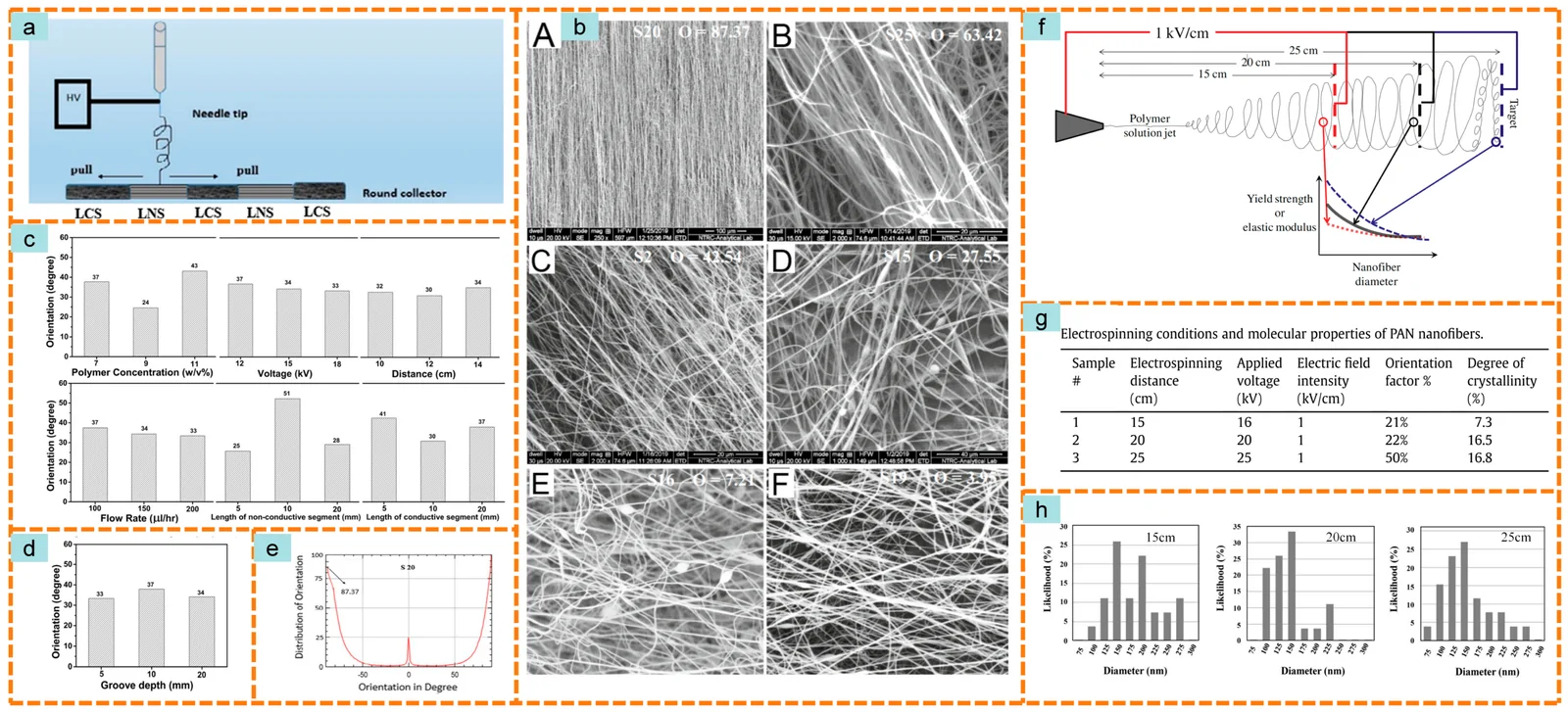
(**a**) Schematic of the needle electrospinning setup; (**b**) SEM images of nanofibers with (**A**–**F)** different orientations; (**c**,**d**) orientation distribution; (**e**) effects of different variables on fiber orientation [34]; (**f**) schematic of size effects on mechanical properties and electrospinning parameters; (**g**) electrospinning conditions and molecular properties of PAN nanofibers; (**h**) diameter distribution at 1 kV/cm for different collection distances [35].

Malik et al. [34] showed that high, controllable alignment in electrospun PAN nanofibers arises from the interplay of four coupled flow and field effects: high-voltage elongational flow drives molecular alignment, a directional electric field dictates the preferred orientation axis, solvent diffusion and evaporation kinetically form the oriented structure, and physical confinement further improves alignment uniformity. The coordinated action of these four factors enables the production of PAN nanofibers with high, tunable orientational order. To realize this control, the authors employed a segmented cylindrical collector with alternating conductive and insulating strips, as shown in Figure 4a. Axial electric field lines form between adjacent conductive segments, creating stable localized directional fields above the insulating regions that steer the electrospun jets into alignment along the collector axis. Experimental measurements confirmed that insulating segment length is the key parameter controlling alignment quality. Optimal insulating segment dimensions produce uniform, stable directional fields, yielding a maximum alignment angle of 87.37° and substantially improving alignment uniformity across the fiber mat, as shown in Figure 4b,c. Solvent evaporation and diffusion act to kinetically stabilize the transient oriented conformation of the jet. Increasing the collection distance extends jet flight time and allows more complete solvent volatilization, which suppresses fiber coalescence and structural relaxation upon deposition, thereby locking in the oriented morphology. An excessively long collection distance, however, reduces the effective electric field strength and provides insufficient jet stretching, degrading overall alignment efficiency. Conversely, overly high applied voltage amplifies jet whipping instability and counteracts the alignment benefits of directional electric guidance, as shown in Figure 4c. Grooved textures on the collector surface introduce physical confinement that acts in concert with the electric field to further refine alignment precision, as shown in Figure 4d. Groove depth limits lateral fiber displacement via physical constraints, working with the axial electric guidance to improve alignment consistency along the collector axis. This dual-field synergy yields more uniformly aligned structures with fewer structural defects, as shown in Figure 4e.

Naraghi et al. [35] described three interrelated flow phenomena that together determine the degree of alignment in electrospun PAN nanofibers: elongational flow provides the driving force for molecular alignment, solvent evaporation-diffusion kinetically locks in oriented conformations, and bending-induced instability homogenizes the orientation distribution. Effective coordination of these three flow effects is essential for producing PAN nanofibers with high orientational order. At a constant electric field strength, the tip-to-collector distance governs jet flight time, which in turn modulates the intensity of elongational flow and the kinetics of solvent evaporation and diffusion, as shown in Figure 4f. Short tip-to-collector distances limit jet flight time, restricting solvent evaporation and leaving substantial plasticizing solvent within the deposited nanofibers. This allows substantial relaxation of pre-aligned PAN chains, such that the orientation factor remains in the low range of 21–22%, as shown in Figure 4g. Longer distances expose the jet to sustained tensile drawing and repeated perturbations from high-order bending instability. Accelerated convective solvent loss progressively increases jet viscosity, allowing sustained shear stress to act on PAN macromolecules and promote thorough chain disentanglement and axial alignment. Under these conditions, the oriented molecular conformation is rapidly kinetically trapped, and a maximum orientation factor of 50% is reached at the longest tested tip-to-collector distance of 25 cm, as shown in Figure 4g. A clear correlation between fiber diameter and orientation degree emerges from the interplay between radial solvent diffusion and fiber dimensions. Nanofibers thinner than 300 nm have a high specific surface area, such that convective solvent removal from the surface proceeds much faster than internal radial diffusion. This mismatch produces dense structures with highly aligned surface layers and low residual solvent content, enhancing both molecular alignment and its long-term stability. Thicker fibers, by contrast, retain significant residual solvent in the core, which readily promotes chain relaxation and reduces overall orientational order, as shown in Figure 4h. Orientation values were measured by polarized Raman spectroscopy for nanofibers electrospun from 9 wt% PAN/DMF solutions at an applied electric field of 1 kV/cm.

The extent of molecular orientation in electrospun PAN nanofibers depends on a combination of parameters: electric field strength, solution concentration, feed rate, and tip-to-collector distance. Solution viscosity and concentration exert the strongest influence, as highly viscous solutions tend to promote jet instability and cause large variations in alignment. Solvent evaporation rate also plays an important role, as faster solvent removal drives rapid solidification that preserves the molecular alignment established during jet elongation. Therefore, Orientation degree can be adjusted systematically by co-optimizing intrinsic solution properties (viscosity, conductivity, polymer molecular weight) and extrinsic processing parameters (electric field gradient, feed rate, collection geometry). Such multi-parameter control allows the orientation distribution to be tailored from isotropic to highly anisotropic, with direct consequences for the mechanical performance and thermal stability of the resulting PAN-derived carbon nanomaterials. Advanced process monitoring via high-speed imaging and in situ rheological characterization has further clarified the complex interplay between jet instability dynamics and orientational evolution, providing valuable guidance for scaling electrospinning to industrial throughput while preserving structural quality [36].

The orientation achieved during electrospinning represents an upper bound for PAN chain alignment established in a single step, without multi-stage hot drawing. After stabilization and carbonization, this orientation is retained, but the resulting carbon nanofibers (typically below 500 nm in diameter) do not reach the tensile strength of industrial continuous tows. Two structural reasons account for this gap: first, the absence of multi-stage drawing prevents further amplification of crystalline orientation and microfibrillar coalescence; second, the small filament diameter and surface-dominated defect population limit the statistical strength ceiling. The cyclization and carbonization sequence itself follows the same pathway described in Section 2.2.4, but the starting orientation set during jet elongation cannot be refined downstream. The trade-off between alignment and tow scalability remains the central limitation of this route. Electrospinning is therefore best understood as a model system for probing the upper limits of flow-induced orientation in PAN, rather than a direct route to high-performance carbon fiber.

#### 2.2.3. Crystallization-Driven Orientation During Hot-Drawing

Hot drawing represents a key thermomechanical step in PAN precursor manufacture that establishes high levels of molecular orientational order. Controlled temperature windows, drawing speeds, and draw ratios coordinate to modulate molecular chain dynamics and crystallization kinetics, thereby shaping the hierarchical orientational architecture of the final fiber. This treatment drives a structural evolution from largely amorphous, mobile chain arrangements to semicrystalline alignment, with the applied stress field promoting the nucleation and growth of oriented crystalline domains. The resulting multiscale orientational structure, from intramolecular chain extension to supramolecular crystallite alignment, translates directly into improved tensile strength and thermo-dimensional stability in the resulting precursor fibers [37].

Crystallization and orientational development proceed in a tightly coupled manner during hot drawing. When subjected to simultaneous thermal activation and tensile stress, PAN molecular chains undergo a conformational shift from random coiled states to highly ordered arrangements, giving rise to oriented crystalline domains that in turn serve as nucleation sites for subsequent crystal growth [41]. The resulting crystalline regions act as robust physical crosslinks that anchor adjacent amorphous chain segments and substantially suppress chain relaxation and disorientation. This reinforcing effect directly improves the mechanical properties of PAN precursors, most notably tensile strength and elastic modulus [42].

Temperature and drawing rate are the two primary parameters governing crystallization-mediated orientational development. At temperatures approaching the glass transition, enhanced segmental thermal motion imparts sufficient mobility to the molecular chains. Applied tensile stress drives progressive chain alignment along the draw direction, establishing a pre-oriented structural state. As temperature rises further, these pre-aligned segments undergo folding and stacking to form locally ordered crystalline precursors. Crystal nuclei propagate via surface-induced nucleation, attracting adjacent chains to adsorb and grow along specific crystallographic planes, and ultimately producing uniformly oriented lamellar crystallites.

Applied tensile stress lowers the free-energy barrier for crystal nucleation by a factor of 2–3, accelerating nucleation kinetics and favoring preferential crystal growth along the drawing direction to yield highly oriented crystalline structures [43]. At low drawing rates, elevated temperatures promote chain disentanglement and rearrangement, facilitating both crystallization and orientational refinement. By contrast, rapid drawing at lower temperatures restricts crystal growth and generates a high density of fine crystallites, which improves fiber toughness and initial modulus. However, excessively high drawing rates can cause localized overstretching and permanent fiber damage.

Reported crystallinity and orientational responses to hot drawing vary notably across studies, driven by the competing interplay of chain relaxation and stress-induced crystallization. At low drawing rates close to the glass transition temperature, chain disentanglement and conformational rearrangement dominate, yielding high crystallinity alongside moderate gains in orientational order. At high drawing rates below the glass transition temperature, crystal growth is kinetically constrained, resulting in high orientational order but low crystallinity with a fine-grained crystalline morphology. Differences in initial precursor structure, particularly the degree of pre-orientation inherited from spinning, further amplify these effects: highly oriented precursors exhibit smaller crystallinity improvements during drawing, as their chain conformations are already extended and provide less driving force for folding and stacking.

#### 2.2.4. Orientation Inheritance Across Pre-Oxidation and Carbonization

The preservation and evolution of orientational order across pre-oxidation and carbonization dictate the conversion of PAN precursors into high-performance carbon fibers. This structural continuity arises from progressive rigidification of chain conformations paired with stepwise chemical transformation, with some precursor structural features retained while others are substantially remodeled. It encompasses a set of coupled processes, including intermolecular crosslinking, oriented growth of graphite microcrystals, and defect mitigation, which enable the retention and progressive refinement of multiscale orientational architectures across all processing stages and ultimately determine the final mechanical performance of the derived carbon fiber.

Pre-oxidation is a complex thermochemical process generally performed in air at 200–300 °C in the general industrial process. During this stage, pendant nitrile groups undergo cyclization, oxidation, and crosslinking reactions to form thermally stable ladder-type aromatic structures [44]. The global axial orientational framework of molecular chains established during spinning and drawing is largely preserved. It should be noted that this molecular-level promotion of cyclization does not always translate into better mesoscale void retention. The competition between thermal relaxation and chemical fixation during stabilization proceeds through three kinetic regimes. Below the glass transition temperature (Tg ≈ 85–90 °C), segmental motion is kinetically frozen and the precursor orientation is preserved mechanically. Above Tg but before appreciable cyclization (~90–200 °C), chains gain mobility and tend to contract toward random-coil conformations; without applied tension, axial orientation measurably decays in this window. Once ladder conversion reaches roughly 40–60% (~220–260 °C), a percolating crosslinked network forms and chain segments are chemically immobilized; from this point onward orientation is fixed regardless of further temperature rise. Slower heating allows cyclization to keep pace with thermal motion, whereas applied tension delays disorientation by suppressing random-coil contraction.

The pre-existing orientational architecture of the PAN precursor is reinforced through constrained anisotropic thermal shrinkage, accompanied by a reduction in crystallinity and restricted segmental mobility. Under applied thermal stress, residual crystalline domains reorient preferentially along the fiber axis and become progressively incorporated into the growing ladder structure [45]. These ladder domains act as a structural template that guides subsequent thermochemical reactions. The overall skin-core radial gradient of chain packing density is also inherited from the precursor. Cyclization initiates preferentially in regions with high initial orientation, which increases chain rigidity and suppresses disordered segmental motion at elevated temperatures. Initial PAN crystallinity decreases progressively as cyclization reactions disrupt regular chain stacking. Ladder-type aromatic crosslinked structures form and gradually replace the original crystalline phase. These ladder act as permanent physical crosslinking junctions that preserve the molecular chain orientation developed during spinning and pre-drawing. Skin-core orientational gradients are partially smoothed as crosslinking rigidifies chain conformations, but radial heterogeneities in reaction extent persist and may amplify at extended treatment times. Chain-end scission and irregular crosslinking also introduce localized orientational defects.

Carbonization is carried out under inert atmosphere at 800–1500 °C and involves dehydrogenation, condensation polymerization, and incipient graphitization of the pre-oxidized fiber, and high temperature graphitization for structural characterization extends up to 3000 °C. The axial preferential orientation of the polymer backbone is inherited and further transferred to the developing turbostratic graphite microcrystals. Non-carbon elements, including nitrogen, hydrogen, and oxygen, are released as small-molecule byproducts, while the remaining carbon atoms rearrange and condense into turbostratic graphite microcrystalline structures. The oriented framework established during pre-oxidation acts as a structural template for carbonization, guiding further chain alignment along the pre-existing orientation direction at high temperature. This templating effect drives the preferential alignment of graphite microcrystals along the fiber axis, enabling both the inheritance and further enhancement of orientational order. The elongated morphology of mesoscale microvoids along the fiber axis is preserved throughout pyrolysis. Original PAN crystalline domains are fully replaced by turbostratic graphite stacks. Crystallite size increases progressively with temperature, accompanied by improved in-plane structural order. Microvoids undergo axial elongation and partial collapse, with rising aspect ratios and decreasing relative volume as carbonization proceeds. Application of moderate tensile stress during carbonization effectively suppresses transverse growth of graphite microcrystals, promotes axial alignment, and further elevates both orientation degree and mechanical performance.

Precise tuning of tensile stress through pre-oxidation and carbonization is essential for minimizing orientational defects and improving alignment uniformity [46]. During pre-oxidation, chain scission and crosslinking generate defects such as chain-end fractures and irregular crosslinked networks, which cause chain misalignment, reduce overall orientational uniformity, and introduce local orientational heterogeneity. The inherent skin-core structure of PAN precursors amplifies these defect gradients via non-uniform oxygen diffusion, creating radial orientation gradients that translate into spatial variations in mechanical properties. Moderate tensile stress counteracts thermally induced disorientation, directs microcrack propagation along the fiber axis, reduces microvoid aspect ratios, and alleviates stress concentrations arising from fiber shrinkage [47].

Carbonization involves pyrolysis and extensive chain rearrangement, which can induce orientational disorder and randomization in defect-prone regions and degrade alignment uniformity. Highly crosslinked or over-oxidized domains formed during pre-oxidation often show poor orientation retention during carbonization, with negative consequences for the final mechanical properties of the carbon fiber. Appropriately applied tensile stress mitigates shrinkage-related defects, preserves the oriented template structure, and improves the mechanical performance of the resulting carbon fibers [48].

The extent of orientational inheritance and structural evolution during thermal treatment is strongly dependent on stabilization and carbonization conditions. Temperature ramp rate, maximum treatment temperature, applied tensile stress, and atmospheric composition all modulate the balance between orientational preservation and structural modification. Slow heating rates during pre-oxidation allow cyclization to keep pace with thermal relaxation, so the rigidity crossover is reached at a lower temperature and the window of uncontrolled chain contraction is minimized. This also promotes uniform cyclization and reduces skin-core reaction heterogeneity. Excessively high temperatures or prolonged holding times during carbonization cause excessive graphitic crystallite growth and defect coalescence, which can degrade overall orientational uniformity despite well-aligned precursor structures. Applied tensile stress throughout thermal processing is a critical process parameter: moderate tension suppresses thermal relaxation of oriented chains and promotes axial graphitic alignment, while excessive stress induces chain fracture and microvoid propagation.

### 2.3. Structure–Property Relationships Between Orientation and Performance

#### 2.3.1. Mechanical Properties

**Figure 5:**
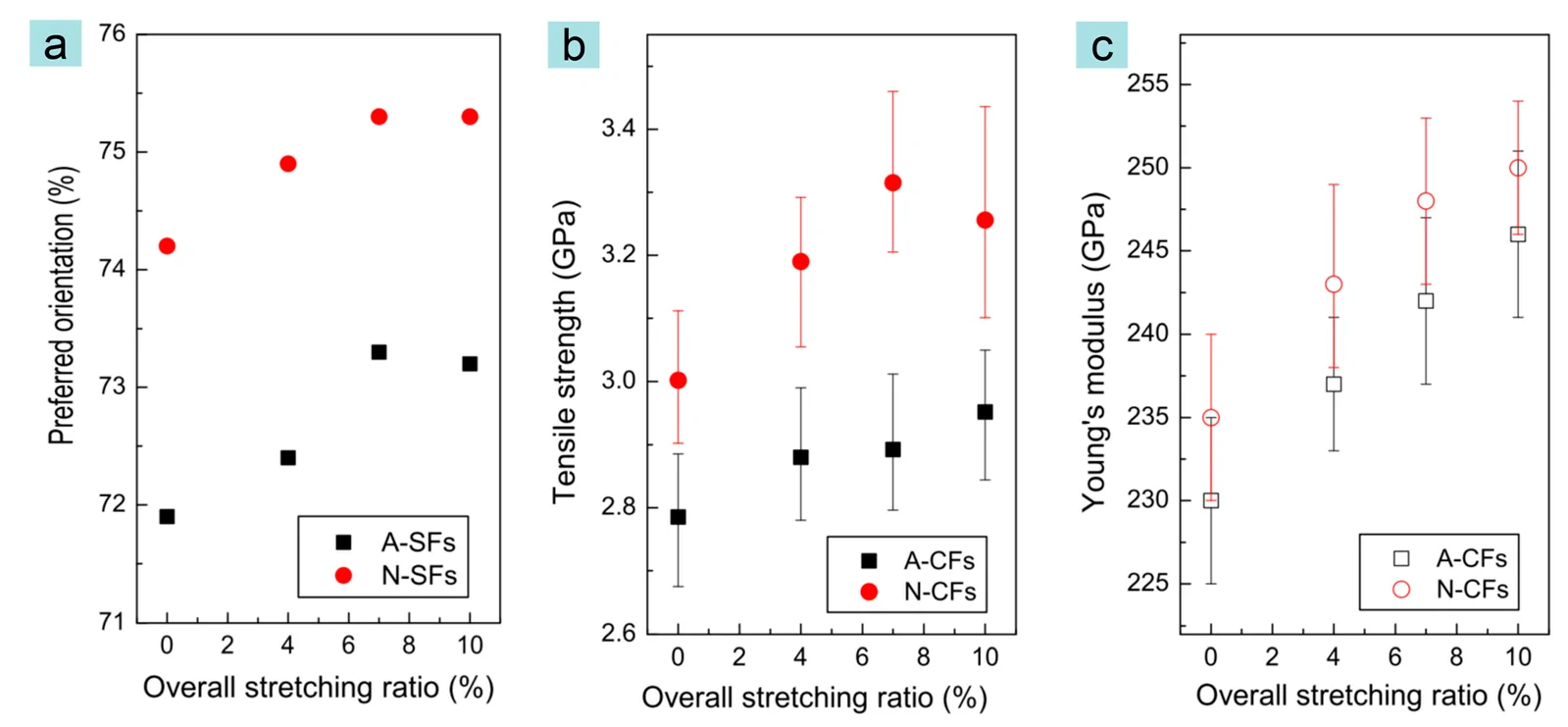
(**a**) Preferred orientation; (**b**) tensile strength; (**c**) Young’s modulus of CFs [14].

Multiscale orientational order in PAN fibers governs the mechanical performance of both precursor fibers and their derived carbon fibers. Molecular chain alignment and crystalline orientation determine both tensile strength and elastic modulus, with the magnitude of improvement scaling with the degree of orientational order across the molecular, crystalline, and microfibrillar length scales [14,42,49], as illustrated in Figure 5.

Higher molecular orientation correlates directly with higher tensile strength. As the orientation factor increases, molecular chains pack more regularly along the fiber axis, strengthening interchain hydrogen bonding and van der Waals interactions while reducing random segment distributions and structural defects. This ordered arrangement optimizes load transfer efficiency under applied stress [50,51]. A rise in the PAN precursor fiber orientation factor from 81.6% to 90% produces a nearly 2.6-fold increase in tensile strength, from 2.4 to 6.2 cN/dtex [25]. These values were obtained for wet-spun PAN homopolymer fibers after boiling-water drawing. Chain orientation was quantified by polarized Fourier-transform infrared spectroscopy, and tensile strength was measured via single-filament testing and reported in cN/dtex, a specific-strength unit normalized by fiber linear density.

Tensile modulus exhibits a similarly strong dependence on orientational order. Highly aligned chains reduce interchain slippage under load, requiring greater force to induce deformation [52]. High levels of drawing-induced orientation in PAN precursors translate directly into elevated modulus in both precursors and carbon fibers [15]. For carbon fibers, a higher preferred orientation factor of graphite microcrystals corresponds to progressive increases in modulus.

Modulus enhancement arises from two synergistic structural effects. First, preferential orientation promotes the growth of graphite microcrystals into ordered lamellar structures that impede crack propagation and dissipate stress. Second, it restricts chain mobility in amorphous domains, suppressing plastic deformation and increasing structural rigidity. For PAN-derived carbon fibers, the orientation factor typically increases with draw ratio until reaching a plateau; at extreme draw ratios, it may decline, with corresponding drops in tensile strength and modulus, as shown in Figure 5. Excessive orientational order can induce chain scission and micropore formation, leading to a counterintuitive reduction in overall mechanical performance. Modulus values were derived from WAXD-measured graphite preferred orientation of carbon fibers produced via continuous pre-oxidation at 180–230 °C and stretched by the ratio range of 0–12% in nitrogen before oxidation, followed by carbonization at temperature range from 350 °C to 1300 °C in high-pure nitrogen with a stress of 15 MPa.

Quantitative correlations between orientation factor and tensile strength vary noticeably across published studies. For instance, an orientation factor increase from 0.7 to 0.9 yields a strength rise from 2.4 to 4.7 GPa for carbon fibers in the dataset of Qin et al. [14], whereas other studies report smaller strength gains at equivalent orientation improvements. Three main sources account for this scatter. First, precursor comonomer composition and impurity content directly affect defect density during carbonization, so the same macroscopic orientation factor can correspond to very different microdefect populations. Second, thermal stabilization and carbonization protocols vary widely between laboratories; optimized temperature-ramp profiles and applied tension better preserve orientational order and produce higher strength at the same precursor orientation level. Third, fiber diameter and tow size introduce statistical effects: finer single filaments generally approach the intrinsic strength of the material more closely than large tows with inherent inter-filament property dispersion.

Beyond orientational order, multiple complementary structural features and processing parameters collectively determine the final mechanical performance of PAN-derived carbon fibers. Crystallinity and crystalline domain size act in concert with orientation to govern elastic modulus: higher crystallinity reinforces stiffness but may reduce toughness if accompanied by large defect populations. Defect density, including chain-end scissions, microvoids, and grain boundary dislocations, directly limits achievable tensile strength by initiating crack propagation; highly oriented structures with high defect density can still fail at low applied loads. Mesoscale void structure further modulates mechanical response: elongated acicular voids aligned along the fiber axis minimize stress concentration, whereas irregular, isotropic pores act as critical failure initiation sites. Precursor molecular weight and copolymer composition also exert strong indirect effects: higher molecular weight reduces chain entanglement density and improves orientation efficiency during drawing, while comonomers modulate cyclization kinetics during stabilization and influence defect formation during carbonization.

Direct cross-study comparison of absolute orientation-performance values requires careful matching of precursor copolymer composition, processing history, and characterization methodology.

It should be noted that these numerical ranges reflect specific published precursor-carbon-fiber datasets rather than the commercial performance ceiling. Commercial high-strength PAN-based carbon fibers such as T1000 and T1100 reach tensile strengths of 6–7 GPa through a combination of higher molecular weight, tighter defect control, and optimized multi-stage drawing and carbonization conditions. The orientation-property correlations discussed here quantify the contribution of orientational order within a given processing framework, not the absolute upper limit of fiber strength.

#### 2.3.2. Thermal Properties

Orientational order exerts a strong influence on the thermal behavior of PAN fibers by modulating chain mobility, intermolecular interactions, and thermal degradation pathways [49,53]. Highly oriented fibers exhibit higher glass transition temperatures, improved thermal stability, and reduced thermal shrinkage compared with poorly oriented counterparts, and these effects carry through to the resulting carbon fibers.

**Figure 6:**
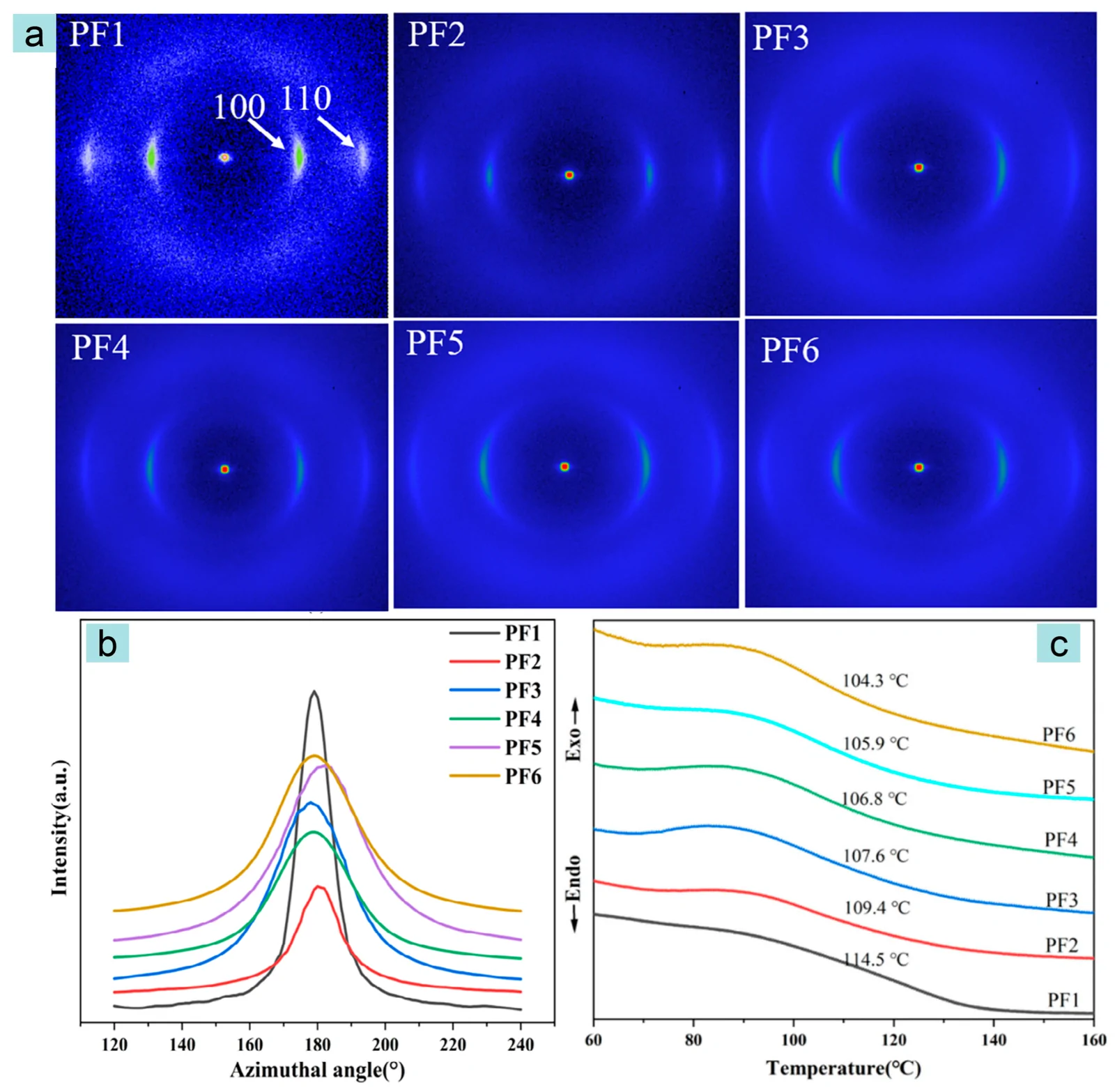
(**a**) Typical 2D WAXD images; (**b**) azimuthal scanning curves at 2θ = 17° for precursor fibers; (**c**) glass transition behavior [54].

High orientational order restricts segmental mobility, which elevates the glass transition temperature. Dense molecular packing and strengthened hydrogen bonding and van der Waals interactions in highly oriented PAN fibers suppress chain motion at elevated temperatures, inhibiting premature chain scission and thermal degradation, as shown in Figure 6 [54,55,56,57,58,59,60]. This stabilization effect becomes more pronounced during thermal processing. Oriented architectures facilitate the formation of thermally stable ladder-type structures during pre-oxidation and the ordered growth of graphite microcrystals during carbonization, both of which improve resistance to thermal decomposition.

Thermal shrinkage upon heating arises primarily from chain disorientation and relaxation. High degrees of orientational order mitigate this shrinkage through rigidified chain networks and intermolecular constraints that lock in the oriented structure. Axial alignment of molecular chains and crystalline domains also produces markedly anisotropic thermal conductivity, with conductivity along the fiber axis substantially higher than in the radial direction, a key property for high-performance carbon fiber applications requiring tailored heat transfer.

#### 2.3.3. Chemical Stability

Chemical stability in PAN fibers scales with the degree of orientational order [61,62]. The extent of molecular chain alignment and the density of chain packing directly govern resistance to chemical degradation. Highly oriented PAN fibers exhibit enhanced chemical inertness because ordered chain arrangements minimize accessible surface area and reduce diffusion pathways for corrosive reagents [63].

Dense intermolecular packing in highly oriented fibers restricts reagent penetration into the fiber bulk and reinforces hydrogen bonding and van der Waals interactions, suppressing chemical attack-induced chain scission or structural disruption. PAN precursors with high orientational order consistently show superior resistance to acid, alkali, and oxidative degradation, a property that is inherited and amplified in the resulting carbon fibers.

#### 2.3.4. Moisture Absorption

Moisture uptake in PAN fibers correlates with orientational order through associated changes in surface topology, pore size distribution, and interchain packing density [64,65]. Poorly oriented fibers typically show higher moisture absorption due to looser chain packing, larger interchain gaps, and more porous internal structures that provide pathways for moisture penetration. Highly oriented fibers feature dense chain alignment and reduced void volume, resulting in lower moisture uptake. This orientation-dependent property makes PAN fibers tunable for textile applications requiring specific moisture management characteristics [66]. For carbon fiber precursors, however, low moisture absorption is generally preferred to minimize defect formation during thermal processing.

#### 2.3.5. Electrical Properties

**Figure 7:**
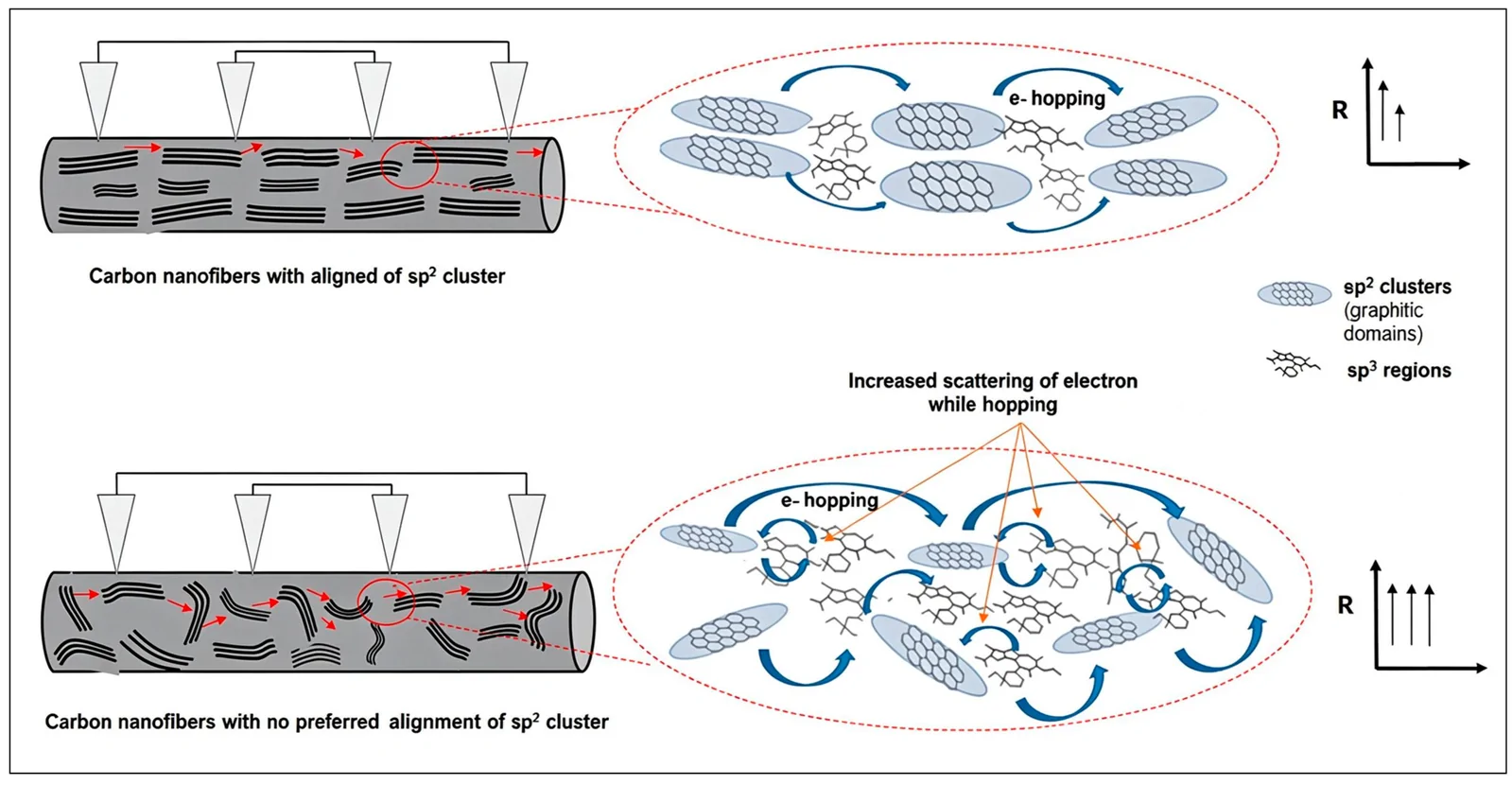
Schematic illustration of the proposed electrical transport mechanism in carbon nanofibers [68].

Electrical conductivity in PAN fibers exhibits a strong dependence on orientational order. Increased molecular chain alignment improves interchain charge transport efficiency by reducing hopping barriers and facilitating ordered charge carrier migration. This orientation-dependent conductivity becomes more pronounced after conversion to carbon fibers. Well-aligned orientational structures promote the formation of continuous graphite microcrystalline networks during carbonization, significantly increasing electrical conductivity, as illustrated in Figure 7. Orientation also modulates piezoelectric behavior. High molecular chain alignment facilitates preferential alignment of dipole-containing moieties along the fiber axis, strengthening the piezoelectric response through enhanced dipole coupling and ordered charge separation under mechanical stress [67].

#### 2.3.6. Optical Properties

The optical properties of PAN fibers, including light transmittance and scattering, correlate directly with the degree of molecular chain alignment [69]. As orientation increases, chains align more uniformly within the fiber matrix, reducing structural inhomogeneities and refractive index fluctuations. Highly oriented PAN fibers exhibit diminished light scattering due to fewer disordered interchain regions, resulting in superior light transmittance. Orientation also modulates surface smoothness and gloss: high orientational order promotes dense, regular molecular packing at the fiber surface, minimizing diffuse scattering and enhancing specular gloss.

PAN fibers also possess intrinsic ultraviolet (UV) resistance [70,71]. The nitrile (-C≡N) groups in PAN molecules absorb UV radiation and dissipate the absorbed energy as heat through molecular vibrations, mitigating UV-induced photodegradation. This UV-shielding effect strengthens with increasing orientation, as improved chain ordering promotes more efficient intermolecular energy transfer, enhancing resistance to UV damage and photochemical aging.

#### 2.3.7. Quantitative Design Targets

Pulling together the structure–property correlations reviewed above, a set of quantitative targets can be defined for a PAN precursor intended for high-strength or high-modulus carbon fiber.

For high-strength carbon fibers (~4–7 GPa), the precursor orientation factor fc should exceed 0.85; crystallinity should be in the 40–55% range (higher crystallinity correlates with strength but also with brittleness and defect sensitivity); the skin-core orientation difference should be less than 0.05 in fc; microvoid aspect ratio should be in the range of 8–20; and single-filament diameter should be controlled to 1.0–1.2 dtex with diameter CV below 2%.

For high-modulus carbon fibers (~350–550 GPa), precursor orientation should be pushed further (fc > 0.90), with higher draw ratios and tighter molecular alignment. Modulus is more sensitive to graphite preferred orientation after carbonization than to precursor crystallinity alone, but the precursor must retain orientation through high-temperature treatment without microvoid coalescence.

Counter-indicators are equally important. Precursor crystallinity above 65% is associated with excessive brittleness and increased defect population during drawing; an orientation factor below 0.7 caps carbon-fiber strength near 2.5 GPa regardless of downstream processing; and large isotropic voids (aspect ratio below 3) act as fracture initiation sites even when bulk orientation is high.

These ranges represent typical values reported in the cited literature, summarized in Table 2, and they are not universal specifications.

## 3. Characterization of Orientational Structures in PAN Fibers

Orientational structures in PAN fibers span multiple length scales, from molecular chains and crystalline domains to mesoscale microfibrils and macroscopic fiber assemblies. Accurate characterization of these hierarchical architectures is a prerequisite for deciphering orientational formation mechanisms and quantifying structure–property relationships. Current analytical approaches to PAN fiber orientation include diffraction, spectroscopic, and microscopic techniques, each with characteristic strengths in spatial resolution, temporal resolution, and accessible length scales. Deployed in combination, these methods provide complementary information that enables comprehensive assessment of orientational order across all relevant structural levels.

### 3.1. X-Ray Scattering Techniques

X-ray scattering methods represent the primary analytical toolbox for PAN fiber orientation characterization, owing to their sensitivity to both crystalline and mesoscale structural features. These techniques exploit coherent scattering of X-rays by electron clouds, where the periodicity of oriented structures generates distinct scattering signatures. Analysis of the azimuthal distribution, intensity variation, and spatial profile of these signals yields quantitative information on orientational order in both crystalline regions and mesoscale architectures.

Based on the scattering angular range, X-ray scattering techniques divide into two broad categories. Wide-angle X-ray diffraction (WAXD) probes crystalline-scale orientation, enabling quantification of crystallite alignment degree and crystallographic texture. Small-angle X-ray scattering (SAXS) targets mesoscale orientation, providing insight into microfibril and lamellar arrangement. Together, WAXD and SAXS constitute a complementary multiscale characterization framework covering the key structural hierarchies of PAN fiber orientation.

#### 3.1.1. Wide-Angle X-Ray Diffraction

WAXD is the benchmark technique for quantifying orientational order in PAN fibers. It should be noted that PAN does not possess a true three-dimensional crystalline lattice; the characteristic equatorial reflection near 2θ ≈ 17° arises from hexagonal packing of chain segments with paracrystalline distortion rather than Bragg diffraction from a perfect crystal. The “crystalline orientation factor” notation used below should be read as the orientation factor of paracrystalline domains. The azimuthal distribution of these signals reflects the angular distribution between crystal plane normals and the fiber axis, enabling quantitative assessment of crystalline orientational order.

For PAN fibers, The two equatorial reflections at 2θ ≈ 17° and 2θ ≈ 29° correspond to the (100) and (110) packings in the paracrystalline hexagonal array (interplanar spacings ≈ 5.3 Å and ≈ 3.0 Å, respectively). The stronger (100) reflection is used for orientation analysis because its azimuthal width is less affected by overlapping diffuse scattering from defective interfibrillar regions. A two-dimensional area detector captures the full diffraction ring pattern. The equatorial diffraction arcs are then integrated to yield intensity profiles as a function of azimuthal angle ϕ, from which the full width at half-maximum (H) of the diffraction peak is extracted. The orientation degree (fc) is calculated using the empirical relation:

fc = (180° − H)/180°(1)

where H is the full width at half maximum of the azimuthal intensity profile of the equatorial (100) reflection, and fc is the orientation factor of paracrystalline domains (conventionally termed the crystalline orientation factor in the literature). For perfect axial alignment, H = 0° and fc = 1; for random orientation, H = 180° and fc = 0. This empirical approximation applies exclusively to uniaxially oriented PAN fibers and is consistent with the form used in prior WAXD orientation analyses of PAN precursors [76]. This calculation applies exclusively to uniaxially oriented PAN fibers tested with a 2D area detector using the (100) crystalline reflection at approximately 2θ = 17°. WAXD primarily probes long-range ordered crystalline domains, delivering quantitative information including the degree of crystalline orientation, crystallite dimensions along and perpendicular to the fiber axis, and bulk crystallinity. It is insensitive to short-range order in amorphous regions and cannot directly characterize molecular chain alignment in the non-crystalline phase.

**Figure 8:**
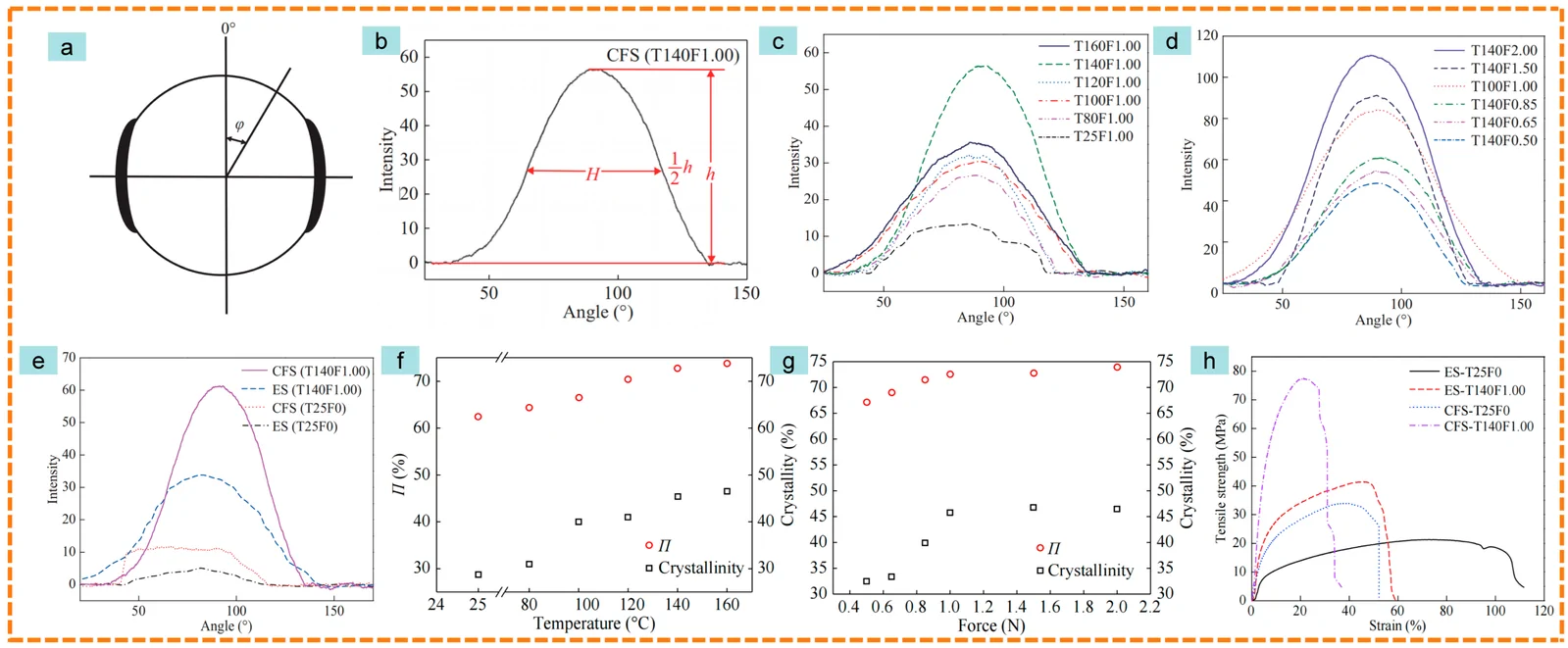
(**a**) Define of the azimuthal angle; (**b**) intensity integration of azimuthal angle; (**c**) azimuthal profile of the (100) reflection for centrifugal spinning PAN nanofibers at different drawing temperatures under a constant force of 1 N; (**d**) profile at different drawing forces under a constant drawing temperature of 140 °C; (**e**) profiles of centrifugal spinning and electrospinning PAN nanofibers after drawing at 140 °C and 1 N; (**f**) orientation degree π of centrifugal spinning PAN nanofibers as a function of drawing force at 140 °C; (**g**) orientation degree as a function of drawing temperature at 1 N; (**h**) stress–strain curves of centrifugal spinning and electrospinning PAN nanofibers at 140 °C under 1 N [76].

Wang et al. [76] employed azimuthal-scanning WAXD to investigate how spinning method, centrifugal spinning (CFS) versus conventional electrospinning (ES), and hot-air bath drawing conditions influence the orientational structure of PAN nanofibers. The equatorial diffraction pattern from a bundle of aligned PAN nanofibers is shown in Figure 8a, and the corresponding integrated intensity profile as a function of azimuthal angle ϕ, from which H is extracted, is presented in Figure 8b. Figure 8c and Figure 8d show the variation in H for CFS-derived nanofibers as a function of drawing temperature and stress, respectively. At a fixed drawing force of 1.0 N, H decreases monotonically with increasing temperature, and at 140 °C, H decreases progressively with increasing drawing stress. The reduction in H directly reflects improved molecular chain alignment and higher crystalline orientational order. Differences in orientation between as-spun and drawn nanofibers are shown in Figure 8e. As-spun CFS nanofibers show moderate initial orientation, which improves slightly after hot-air bath drawing. As-spun ES nanofibers, by contrast, show negligible initial orientation but develop substantial alignment after drawing, highlighting the effectiveness of post-drawing treatment for ES-based materials.

The evolution of orientation and crystallinity in CFS nanofibers with temperature and stress is summarized in Figure 8f,g. Both crystalline orientation degree and crystallinity increase with rising temperature and stress, indicating a synergistic improvement in structural order. Tensile strength profiles for as-spun and drawn nanofibers from both processes are shown in Figure 8h. As-spun CFS nanofibers exhibit substantially higher tensile strength than their ES counterparts, consistent with their higher initial crystalline order. After hot-air bath drawing, both fiber types show marked increases in tensile strength and concomitant reductions in elongation at break. This behavior arises from two synergistic effects: crystallization induced by the combination of temperature and drawing stress, and stress-directed alignment that enhances crystalline domain orientation along the fiber axis.

#### 3.1.2. Small-Angle X-Ray Scattering

SAXS probes mesoscale structures in the 1–100 nm size range in PAN fibers, based on elastic scattering of X-rays by electron density inhomogeneities such as micropores, microfibril bundles, and crystalline–amorphous interfaces. Oriented mesostructures introduce strong anisotropy into SAXS patterns: structures aligned along the fiber axis produce intense scattering signals perpendicular to the fiber axis. Analysis of the shape, azimuthal distribution, and intensity of these scattering patterns yields quantitative data on orientation degree, aspect ratio, and spatial distribution of mesoscale features.

For PAN fibers, SAXS characteristically shows a pair of scattering lobes along the equatorial direction, corresponding to micropores aligned parallel to the fiber axis. The ellipticity of these lobes correlates positively with orientational order: a more flattened elliptical shape indicates a smaller angle between the micropore long axis and the fiber axis, reflecting higher alignment regularity.

The Ruland model is commonly used to derive scattering parameters and orientation-related metrics for micropores, including the mean orientation angle (Beq), micropore length (L), average chord length (Lp), aspect ratio (L/Lp), and relative micropore volume (Vrel). Anisotropic SAXS patterns from axially oriented micropores appear as rhombic features for PAN precursors and elongated streaks for carbon fibers. The scattering intensity distribution along the equatorial direction, perpendicular to the fiber axis, correlates directly with micropore alignment. Smaller Beq values indicate higher axial orientation, while the L/Lp ratio provides a complementary measure of overall orientational order. All microvoid orientation parameters were calculated from 2D SAXS patterns collected at room temperature with a pinhole-collimated X-ray beam, assuming a Gaussian distribution of micropore orientation angles. SAXS targets mesoscale structural features in the 1–100 nm size range, including the orientation, aspect ratio, and spatial distribution of acicular microvoids, microfibril bundles, and crystalline-amorphous interfaces. It does not provide atomic-scale information on molecular chain conformation or individual crystallographic lattice parameters.

Assuming a Gaussian distribution of micropore orientation angles, linear fitting can be used to quantify Beq and micropore length L. The azimuthal width distribution of scattering intensity along the equatorial direction is defined as:

(2) $$B_{\pi /2}(S)=\frac{1}{I(S,\pi /2)}\int I(S,\phi )\mathrm{d\phi}$$

**Figure 9:**
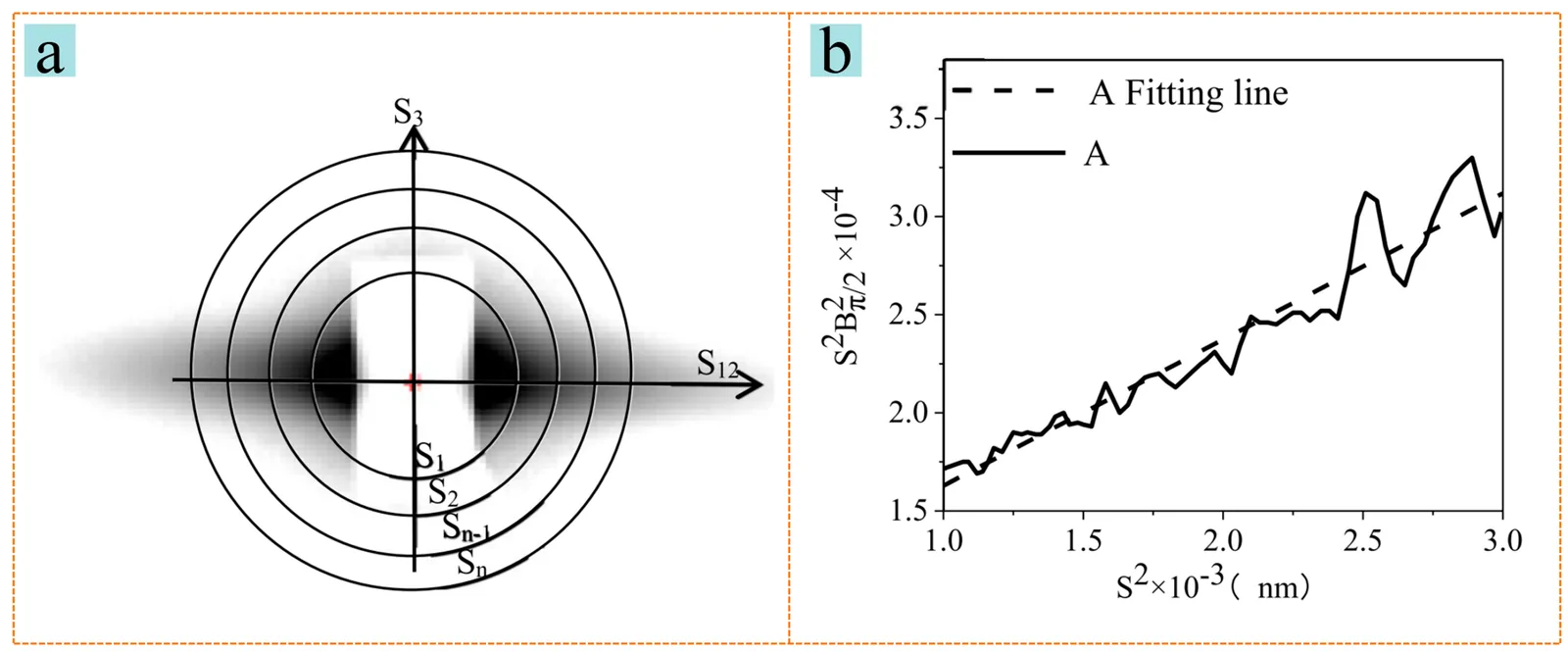
(**a**) Schematic diagram of intensity integration along the azimuth direction; (**b**) S^2^ ~ S^2^B^2^_π2_(S) plot for PAN fiber [31].

In this expression, S = 2sinθ/λ is the scattering magnitude (λ is the X-ray wavelength and θ is half the scattering angle), and B_π/2_(S) is the azimuthal full width at half maximum of the equatorial scattering lobe at a given S in radians. The calculation method for B_π/2_(S) is illustrated in Figure 9a. Using scattering vector S_1_, S_2_, ···, S_n_ as radius, the scattering intensity of the 2D SAXS pattern is integrated over the azimuthal angle *φ*. This yields intensities I(S_1_, π/2), I(S_2_, π/2), ..., I(S_n_, π/2), from which the corresponding Bπ/2(S) values are determined.

This linear relation follows directly from the Ruland model under the assumption of a Gaussian distribution of micropore orientations [31].

(3) $$S^{2}B_{\pi /2}^{2}(S)=1/L^{2}+S^{2}B_{\mathrm{eq}}^{2}$$

where Beq is the mean deviation of micropore long axes from the fiber axis, and L is the micropore length (in nm). Both parameters are obtained from the intercept and slope, respectively, of the S^2^ vs. S^2^B^2^_(π/2)_(S) plot shown in Figure 9b.

(4) $$I(S,\pi /2)\propto n\rho_{m}\;\frac{L^{2}}{\sqrt{L+{\left(\mathrm{SL}B_{\mathrm{eq}}\;\right)}^{2}}}\frac{L_{P}^{4}}{\;{\left[1+{\left(2\pi L_{p}\;\right)}^{2}\right]}^{3/2}\;}\;\;$$

(5) $$V_{\mathrm{rel}}\propto \mathrm{nL}L_{P}^{2}\;$$

where Lp is the average chord length of the electron-density inhomogeneities, and n is the number density of micropores, and Vrel is the relative micropore volume. These parameters are derived from the medium- and high-angle regions of the same 1D SAXS profile used for the orientation analysis above.

**Figure 10:**
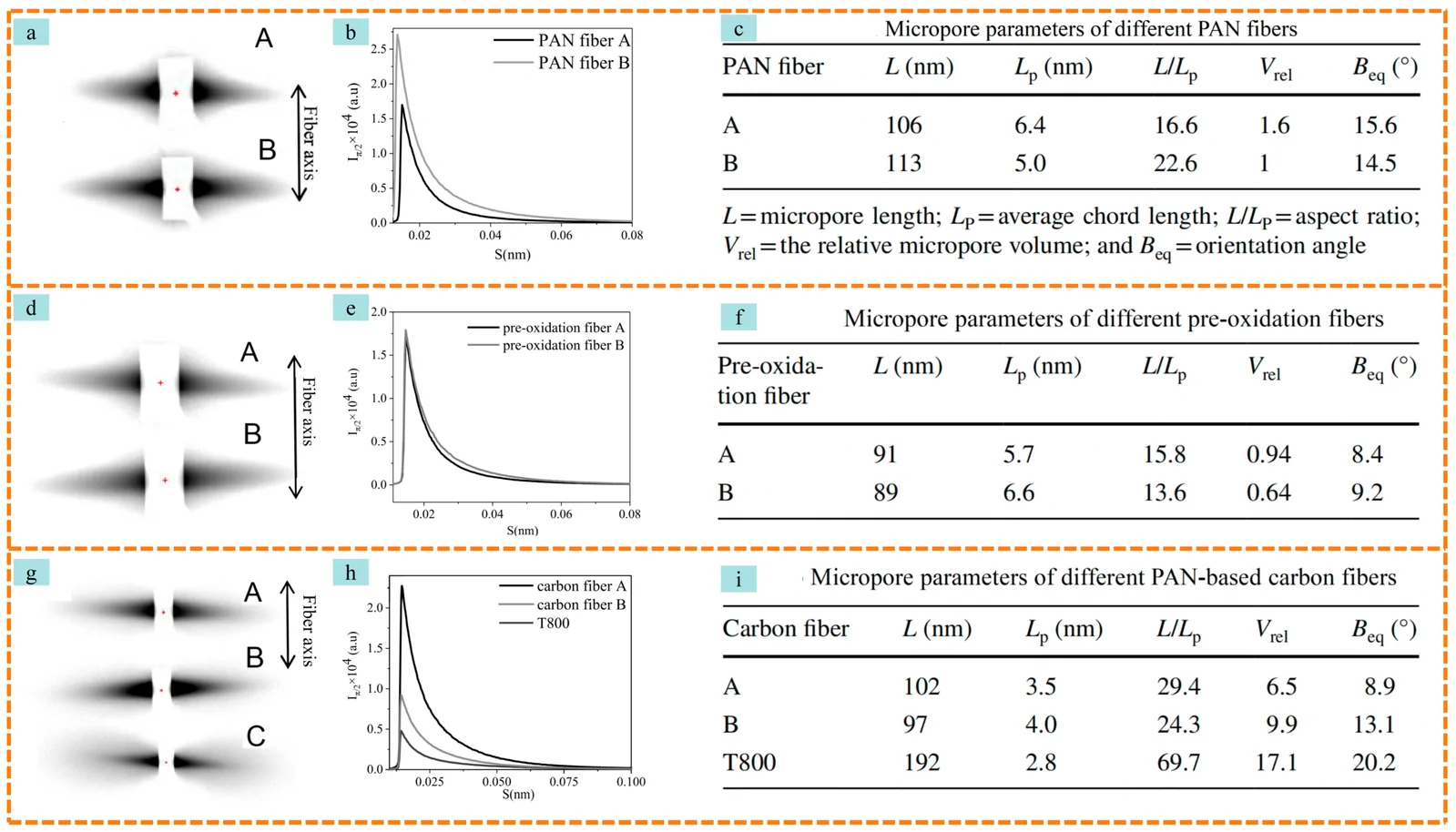
(**a**) 2D SAXS patterns of PAN fibers at room temperature; (**b**) linear SAXS profiles in the perpendicular direction; (**c**) Micropore parameters of different PAN fibers; (**d**) 2D SAXS patterns of PAN fibers after pre-oxidation at 240 °C; (**e**) linear SAXS profiles in the perpendicular direction after pre-oxidation at 240 °C; (**f**) Micropore parameters of different pre-oxidation fibers; (**g**) 2D SASX patterns of different PAN-based carbon fibers at room temperature; (**h**) linear SAXS profiles of different PAN-based carbon fibers in the perpendicular fiber direction [72]; (**i**) Micropore parameters of different PAN-based carbon fibers.

Cui et al. [72] used SAXS to characterize micropore orientation in PAN precursors, pre-oxidized fibers, and carbon fibers as a function of draw ratio, clarifying how draw ratio influences micropore orientation evolution across the full fabrication route. At the precursor stage, fibers with a high draw ratio exhibit stronger initial micropore orientation. Figure 10a shows that 2D SAXS patterns of precursors with different draw ratios all display broad rhombic symmetry, indicating that micropores are highly oriented along the fiber axis with an acicular morphology. The absence of distinct scattering peaks in Figure 10b confirms an aperiodic micropore distribution in PAN precursors. Figure 10c further show that the high-draw-ratio precursor (fiber B) has a larger micropore aspect ratio (22.6 vs. 16.6) and a smaller orientation angle (14.5° vs. 15.6°) than the low-draw-ratio fiber, consistent with higher initial orientational order. This behavior originates from molecular chain alignment during drawing, which deforms micropores into elongated acicular shapes. High draw ratios also induce partial pore collapse, which concentrates the orientation of the remaining pore structure.

During pre-oxidation, the trend reverses at the mesoscale: the low-draw-ratio precursor (fiber A) retains better micropore orientation than the high-draw-ratio one (fiber B). As shown in Figure 10d,e, 2D SAXS patterns of pre-oxidized fibers maintain their rhombic shape, indicating that preferential micropore orientation along the fiber axis is preserved, though with reduced scattering intensity. Figure 10f shows that after pre-oxidation, fiber A exhibits a smaller orientation angle (Beq = 8.4°) and a higher aspect ratio (15.8) than fiber B. This does not contradict the molecular-level argument in Section 2.2.4 that chain alignment promotes cyclization. Rather, it reflects a void-stability effect: the low-draw-ratio precursor has larger, more perfect crystallites and fewer drawing-induced defects. Cyclization disrupts its crystalline framework less severely, so acicular micropores experience less collapse and retain their axial alignment. The high-draw-ratio fiber, despite stronger initial pore orientation, contains more defect-rich small crystallites; vigorous cyclization in these regions causes local pore collapse that partially erases the initial orientation gain.

After carbonization, 2D SAXS patterns appear as elongated streaks perpendicular to the fiber axis (Figure 10g), indicating that micropores remain preferentially oriented along the fiber axis but form denser structures. Figure 10h confirms the absence of periodic scattering peaks, verifying the aperiodic nature of micropores in carbon fibers. As summarized in Figure 10i, the low-draw-ratio fiber A maintains superior micropore orientation (Beq = 8.9°) and a larger aspect ratio (29.4) even after carbonization. This reflects the higher structural stability of fiber A through pre-oxidation and carbonization, which minimizes pore collapse and orientation loss. Commercial T800 carbon fibers exhibit a substantially higher micropore aspect ratio of 69.7 and a larger orientation angle of 20.2°, associated with their highly elongated micropore morphology.

These cross-stage SAXS results quantitatively verify the inheritance and modification of mesoscale orientational structures through thermal treatment. The axial alignment of microvoids is preserved across all processing stages, confirming that the precursor orientational template is transmitted through pre-oxidation and carbonization. At the same time, microvoid morphology is substantially modified: the micropore aspect ratio nearly doubles from precursor to carbon fiber, while the mean orientation angle decreases by nearly half, reflecting both pore elongation and improved overall orientational ordering during high-temperature treatment.

It is worth emphasizing that the SAXS-derived pore orientation and the WAXD-derived crystalline orientation factor are not interchangeable metrics. High draw ratio improves the initial pore orientation but does not guarantee better retention of that orientation through thermal treatment. The optimal precursor design therefore requires balancing initial orientation against defect population and crystallite stability.

### 3.2. Spectroscopic Techniques

Spectroscopic techniques access orientational order at the molecular level by probing the anisotropy of molecular vibrational responses. The two principal methods in this class are Fourier-transform infrared (FTIR) spectroscopy and Raman spectroscopy, both chemically specific and non-destructive. When implemented with polarized light, they reveal orientational characteristics through the differential response of molecular vibrational modes to the polarization direction of the incident beam.

#### 3.2.1. Polarized Fourier-Transform Infrared Spectroscopy

Polarized FTIR spectroscopy quantifies orientational order by measuring the polarization dependence of infrared absorption arising from molecular vibrational dipole transitions. Analysis of the absorption anisotropy of characteristic functional groups enables quantitative assessment of chain alignment. For PAN fibers, the cyano groups stretching vibration at 2240 cm^−1^ serves as the primary marker for orientation analysis due to its high intensity and signal stability. When polarized infrared light is polarized parallel to the fiber axis, absorption by axially aligned cyano groups is enhanced; when polarized perpendicular, absorption is attenuated. This dichroic difference is quantified via the orientation factor (ƒ).

The dichroic ratio (D) is defined as [42]:

D = A_‖_/A_⊥_ (6)

where A_‖_ is the absorbance with the electric vector of the incident radiation parallel to the fiber axis, and A_⊥_ is the absorbance with the vector perpendicular to the axis.

The Herman’s orientation factor is given by [42]:

(7) $$f=\frac{\left(D-1)(2D_{0}+2\right)}{\left(D+2)(2D_{0}-1\right)}$$

where f is the amorphous-chain orientation factor, D is the dichroic ratio defined in Equation (6). D_0_ = 2cot^2^α, where α is the angle between the -C≡N transition-dipole moment and the PAN molecular-chain axis. For perfectly aligned fibers, D approaches infinity and ƒ = 1; for randomly oriented fibers, D = 1 and ƒ = 0. A value of *α* ≈ 70° is typically adopted for uniaxially drawn PAN fibers [44]. The factor was derived from the dichroic ratio of the 2240 cm^−1^ -C≡N stretching band, assuming a fixed 70° angle between the nitrile-group transition dipole and the PAN molecular backbone axis.

**Figure 11:**
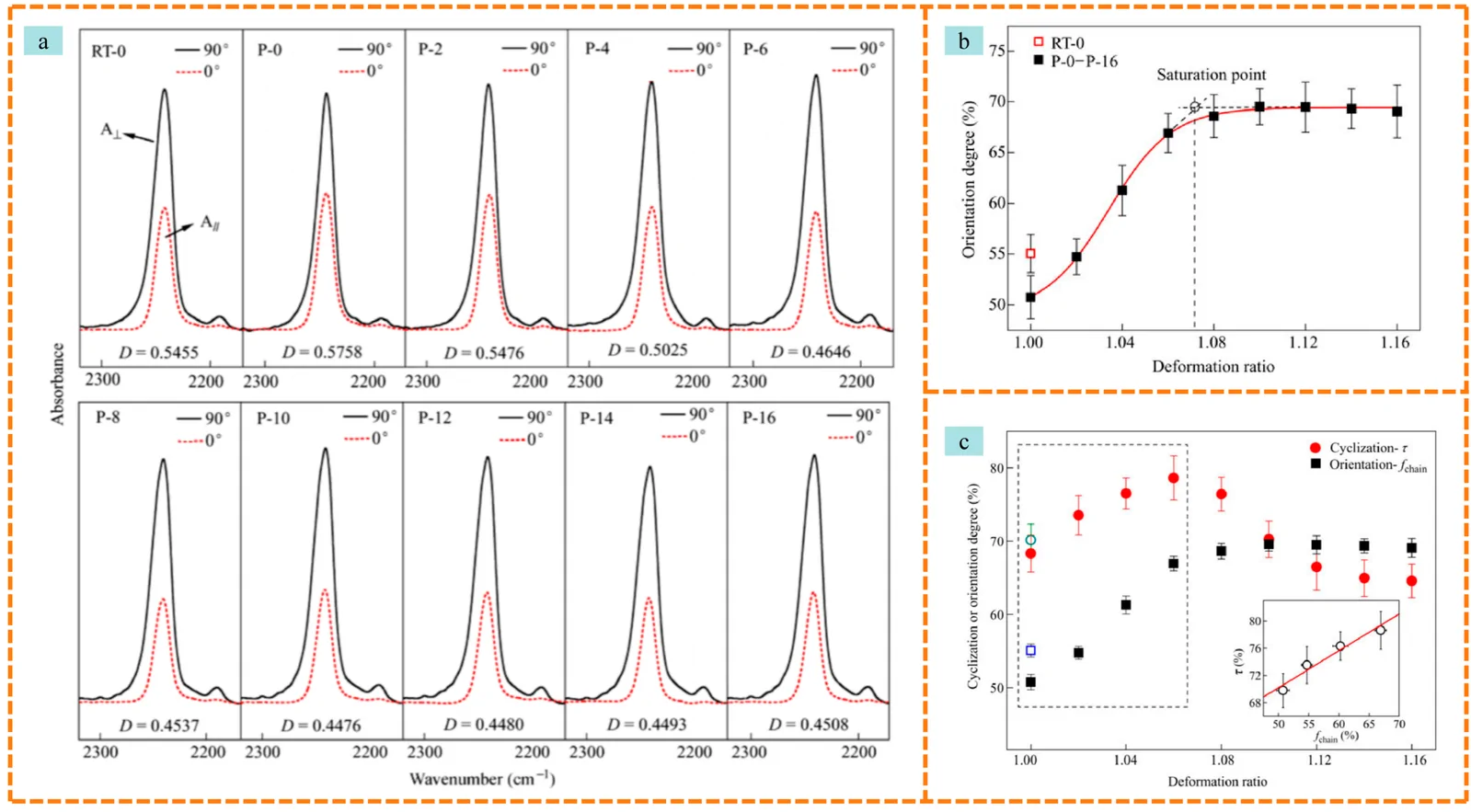
(**a**) Polarized FTIR spectra of the nitrile group absorption; (**b**) evolution of PAN chain orientation during thermal stretching; (**c**) orientation degree after hot-stretching at 180 °C and cyclization degree after stabilization [42].

Liu et al. [42] employed polarized FTIR to investigate chain orientation in copolymer PAN fibers during deformation prior to thermal stabilization, and its influence on the subsequent stabilization process. Polarized FTIR spectra of the -C≡N stretching absorption in the 2200–2300 cm^−1^ region are presented in Figure 11a. Absorbance with the electric vector perpendicular to the fiber axis (A_⊥_) exceeds that with the vector parallel (A_‖_), and the dichroic ratio D decreases progressively with increasing tensile deformation, consistent with the development of preferential chain alignment along the draw direction.

The evolution of the molecular chain orientation degree (f_chain_) during thermal stretching is shown in Figure 11b. Heating as-received PAN fibers from room temperature to 180 °C at constant length causes a marked drop in f_chain_, attributed to increased chain entropy and the release of residual spinning stresses above the glass transition temperature, which produces spontaneous disorientation. When fibers are stretched at 180 °C with deformation ratios λ from 1.00 to 1.08, f_chain_ increases substantially. Beyond λ = 1.08, f_chain_ saturates at approximately 69.5%, indicating that chain alignment has reached its equilibrium limit.

The relationship between orientation and cyclization during stabilization is summarized in Figure 11c. At λ below 1.06, the cyclization degree τ increases with rising f_chain_. Chain alignment along the fiber axis reduces entanglement, steric hindrance, and dipole–dipole repulsion between -C≡N groups, facilitating cyclization. At λ above 1.08, τ declines sharply. This reversal arises from excessive deformation-induced chain yielding, which increases the spacing between -C≡N groups and impedes cyclization.

Polarized FTIR specifically characterizes the orientational state of molecular chains in the amorphous phase via the anisotropic response of polar functional groups, predominantly -C≡N. It provides information on average chain alignment and segmental orientation but cannot resolve the spatial distribution of crystalline domains or mesoscale structural heterogeneities. Polarized FTIR is highly sensitive to molecular chain orientation in the amorphous phase, but its spatial resolution is limited by the infrared spot size, typically 5–10 μm, which restricts detection of radial orientation gradients. Inhomogeneous sample thickness can also introduce absorbance deviations; these effects are mitigated through baseline correction and averaging over multiple scans.

#### 3.2.2. Polarized Raman Spectroscopy

Polarized Raman spectroscopy quantifies molecular orientation in PAN fibers through the anisotropic scattering response of polymer chains to polarized excitation. Its submicrometer spatial resolution enables characterization of local orientational variations within individual fibers.

For PAN fibers, the vibrational modes showing strong polarization dependence are the -C≡N stretching vibration near 2240 cm^−1^ and the C-C backbone stretching vibration near 1394 cm^−1^. Both modes exhibit intrinsic anisotropy arising from the ordered packing of PAN molecular chains. Raman scattering intensity is highly sensitive to the angle between the transition dipole moment of the vibrational mode and the polarization directions of the incident and scattered light. The -C≡N stretching mode is preferred for orientation analysis due to its strong signal intensity and direct correlation with backbone alignment.

Two polarization configurations are used, following Porto notation. Parallel polarization (I_‖_) has both incident and scattered light polarized parallel to the fiber axis. Cross polarization (I_⊥_) has incident light parallel to the fiber axis and scattered light perpendicular to it. The intensity ratio I_‖_/I_⊥_ reflects the degree of molecular chain alignment, with higher ratios indicating more pronounced orientational order along the fiber axis.

(8) $$f=\frac{3\cos^{2}\theta -1}{2}$$

(9) $$\cos^{2}\theta =\frac{I_{//}}{I_{//}+4I_{⊥}}$$

where θ is the angle between the molecular chain axis and the fiber axis; I_⊥_ is the Raman scattering intensity measured with the incident polarization perpendicular to the fiber axis, and I_‖_ is the intensity measured with the incident polarization parallel to the fiber axis. For random orientation, *f* = 0; for perfect alignment along the fiber axis, *f* = 1. This treatment follows the Bower framework for uniaxially oriented polymers [77].

Polarized Raman spectroscopy probes both amorphous chain conformation and crystalline vibrational modes, offering information on molecular-level orientational order with sub-micrometer spatial resolution. It can detect local variations in chain alignment across the fiber cross-section but is less sensitive to long-range mesoscale structures such as microvoid morphology.

**Figure 12:**
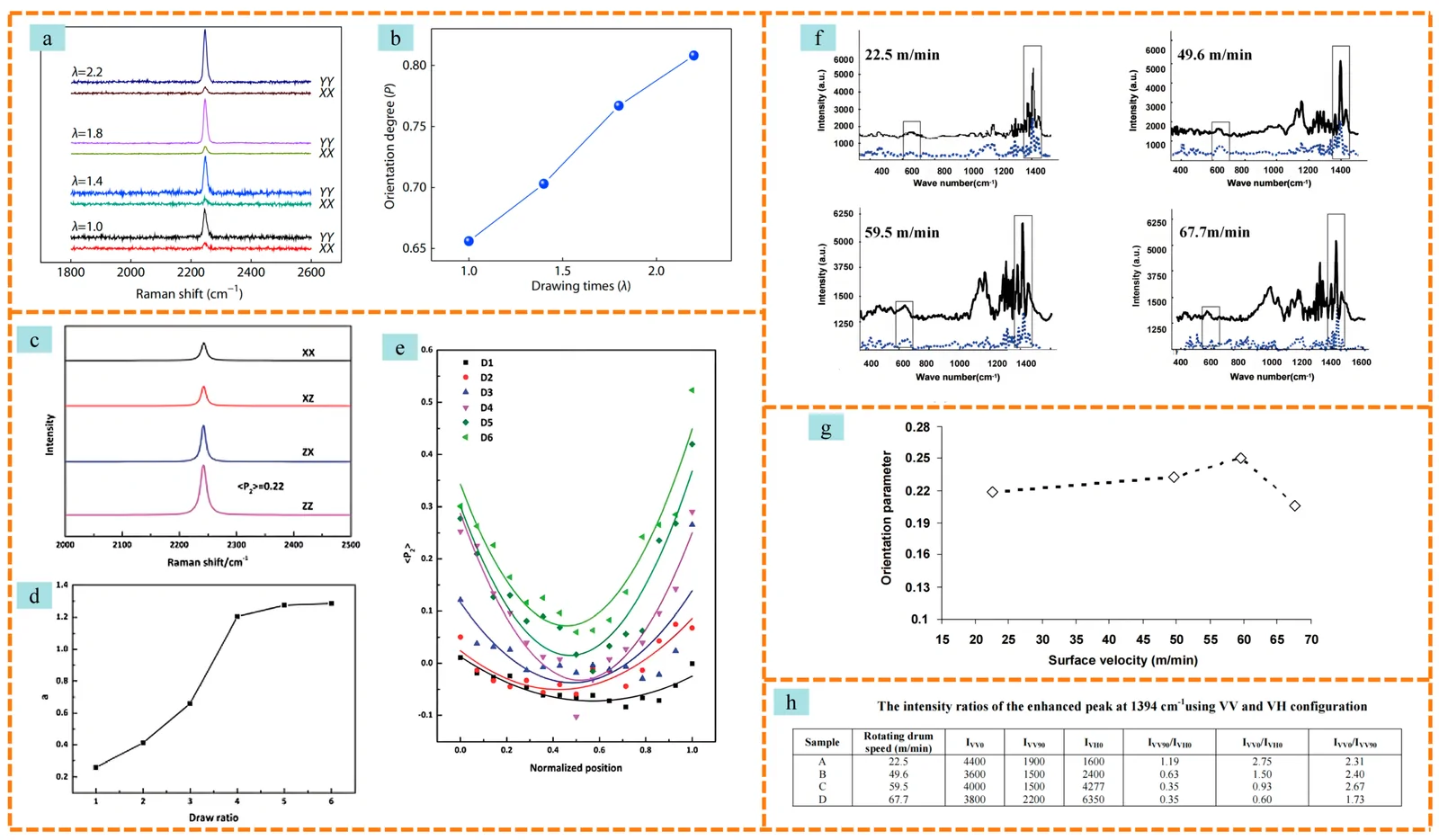
(**a**) Polarized Raman spectra of a single electrospun coPAN nanofiber; (**b**) orientation degree versus drawing time [51]; (**c**) polarized Raman spectra of PAN fiber in the 2000–2500 cm^−1^ region; (**d**) radial distribution of P2 values; (**e**) quadratic coefficient a versus draw ratio [78]; (**f**) Raman spectra under VV mode at different take-up speeds with fiber axis at 0° and 90° to polarization plane; (**g**) orientation parameter versus take-up speed; (**h**) intensity ratios of the 1394 cm^−1^ peak under VV and VH configuration [79].

Xu et al. [51] used polarized Raman spectroscopy to characterize molecular orientation in electrospun PAN nanofibers as a function of draw ratio. Polarized Raman spectra in perpendicular (XX) and parallel (YY) polarization configurations are shown in Figure 12a. The evolution of -C≡N group orientation with increasing draw ratio is presented in Figure 12b; the gradual increase in alignment is attributed to the transition of macromolecular chains from coiled to extended conformations under tensile force.

Sha et al. [78] examined the radial evolution of molecular orientation in wet-spun PAN precursor fibers via polarized Raman spectroscopy. Polarized Raman spectra in the 2000–2500 cm^−1^ region at a representative radial position are shown in Figure 12c, including parallel-polarized (xx, zz) and cross-polarized (xz, zx) configurations. The intensity difference between xx and zz spectra confirms anisotropic molecular orientation. The radial distribution of the orientation parameter P2 for fibers at different draw ratios is plotted in Figure 12d. Overall molecular orientation increases with draw ratio, consistent with enhanced tensile stress. Figure 12e reveals a pronounced skin-core difference in P2 values: orientation in the skin layer increases more strongly than in the core as draw ratio rises. This gradient arises from the rapid reduction in dimethyl sulfoxide concentration in the outer layer during wet-spinning coagulation, which induces instantaneous PAN precipitation and dense skin formation. The core remains in a semisolid state with higher chain mobility, so tensile stress is borne predominantly by the rigid skin layer, producing more efficient chain alignment in that region.

Sadrjahani et al. [79] investigated the dependence of molecular orientation on take-up speed in electrospun PAN nanofibers. Orientation measurements were performed with the fiber axis at φ = 0° (parallel) and φ = 90° (perpendicular) relative to the polarization plane of the incident laser. Raman spectra under VV polarization at these two angles for fibers prepared at different take-up speeds are shown in Figure 12f; the intensity variation in the 1394 cm^−1^ peak was used to quantify orientation. The orientation parameter as a function of take-up speed is plotted in Figure 12g, with a distinct maximum at 59.5 m/min followed by a decline at higher speeds. This reduction at excessive take-up speeds arises from insufficient drawing time: rapid collection leaves inadequate opportunity for macromolecular chains to rearrange and align along the tension direction. Average orientation parameters derived from intensity ratios under VV and VH polarization modes are summarized in Figure 12h.

### 3.3. Optical Birefringence

Optical birefringence provides a well-established approach for quantifying orientational order in polymeric materials. The technique measures the refractive index difference between orthogonal directions in a fiber under polarized light, enabling quantitative evaluation of chain alignment degree. Its relative operational simplicity, fast response, and non-destructive nature make it suitable for routine spinning process optimization and structure–property relationship investigations. A key strength is its ability to capture the bulk-averaged orientational state of fibers, which is particularly valuable for tracking molecular chain orientation dynamics during continuous spinning operations.

Birefringence measurements are typically performed using the Senarmont compensator method with a polarized optical microscope. Individual PAN fibers are mounted flat on a microscope slide, immersed in glycerin to minimize light scattering, and covered with a coverslip.

The fiber birefringence (Δn*_f_*), defined as the refractive index difference between the fiber axial (n_//_) and transverse (n_⊥_) directions, is calculated as [80]:

(10) $$\Delta n_{f}=n_{//}-n_{⊥}=\frac{\lambda }{d}\cdot \frac{\theta }{180}$$

where Δn*_f_* is the fiber birefringence in dimensionless refractive-index units, *λ* is the wavelength of sodium light, *θ* is the average retardation angle, and *d* is the fiber diameter.

The orientation factor derived from birefringence (*f*_Δ*n*_), linking optical anisotropy to molecular alignment, is given by:

(11) $$f_{\Delta n}=\frac{\Delta n_{f}}{\Delta n_{f0}}\cdot \frac{\rho_{\mathrm{cr}}}{\rho_{f}}$$

where *f*_Δ*n*_ is the birefringence-derived orientation factor, Δ*n_f_*_0_ is the birefringence of a perfectly oriented PAN fiber; *ρ_cr_* and *ρ_f_* are the densities of the crystalline phase and the bulk PAN fiber, respectively.

**Figure 13:**
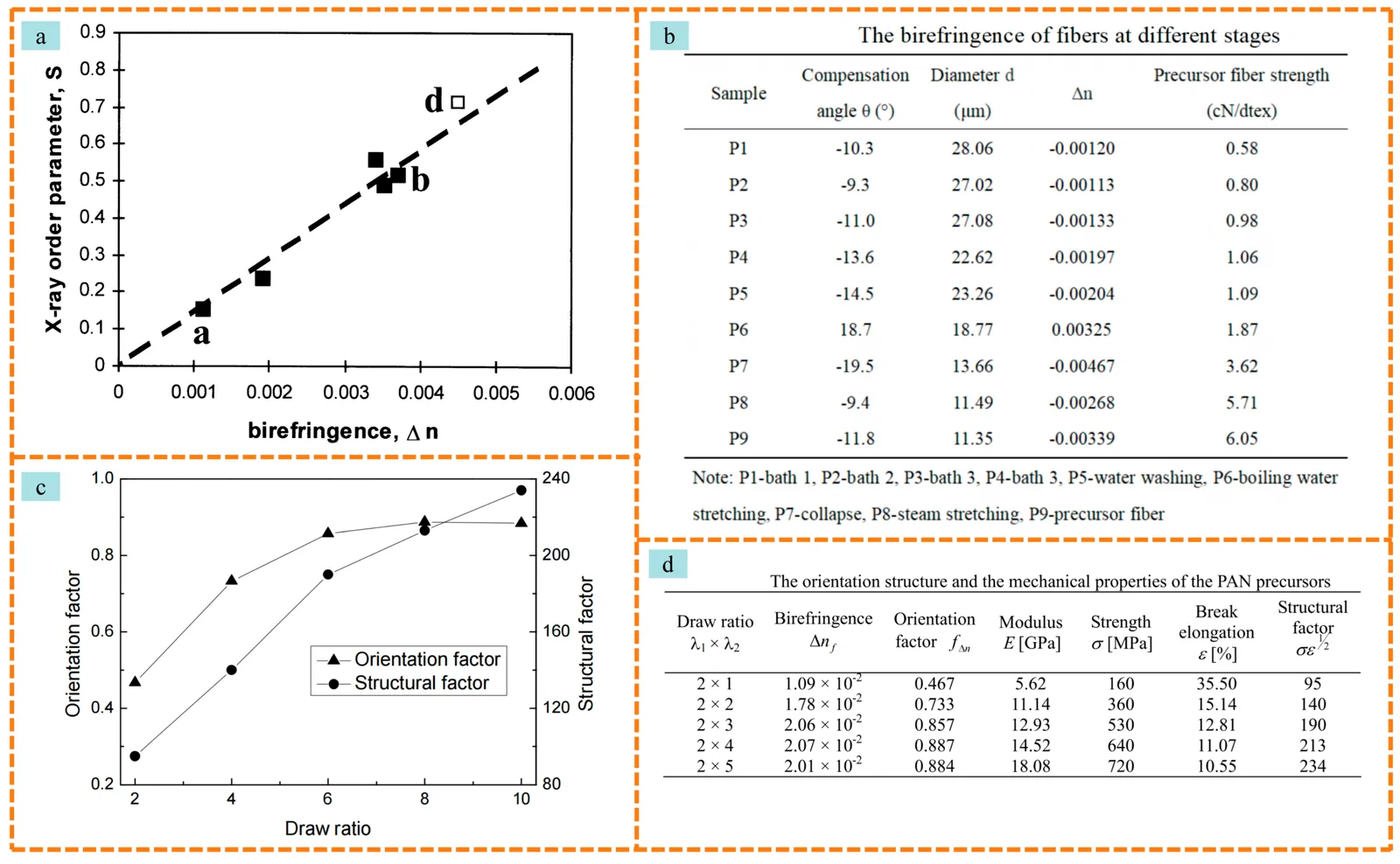
(**a**) Birefringence versus X-ray order parameter for several fibers [81]; (**b**) birefringence evolution across different spinning stages [82]; (**c**) orientational order in PAN precursors as a function of draw ratio; (**d**) orientational order in PAN precursors as a function of draw ratio [80].

Davidson et al. [81] characterized molecular orientation in melt-spun high-acrylonitrile AMLON fibers and solution-spun commercial acrylic fibers using a combination of optical birefringence and X-ray diffraction (XRD). As shown in Figure 13a, the orientational order parameter derived from XRD correlates linearly with fiber birefringence, consistent with the fundamental link between optical anisotropy and molecular alignment. The relatively low birefringence values observed were attributed to the near-perpendicular orientation of highly polarizable -C≡N groups relative to the polymer backbone. For PAN fibers with low comonomer content, highly aligned samples exhibited small, negative birefringence, a phenomenon associated with the unique dipole orientation of nitrile groups in low-comonomer systems. Wang et al. [82] used the Senarmont compensation method to quantify birefringence of PAN precursors across different spinning stages. The data in Figure 13b show birefringence shows a steady upward trend across the spinning sequence, reflecting progressive ordering of polar groups along the macromolecular backbone and increased alignment of chain segments parallel to the fiber axis. Tensile strength evolves in parallel with birefringence, consistent with the intrinsic structure–property coupling in oriented polymer fibers. An et al. [80] determined orientation factors of PAN precursors as a function of draw ratio via polarized microscopy birefringence measurements. Figure 13c,d show that the orientation factor reaches a maximum at a draw ratio of 8. The results confirm a strong correlation between birefringence, molecular orientation, and tensile strength, highlighting the utility of birefringence as an indirect metric for evaluating both orientational order and mechanical performance of PAN fibers.

Due to its fast response, low operational cost, and compatibility with continuous fiber tow processing lines, optical birefringence is the most widely deployed technique for real-time orientation monitoring in industrial PAN precursor production lines.

### 3.4. Microscopic Imaging Techniques

Microscopic imaging delivers direct morphological insight into orientational structures in PAN fibers across length scales ranging from macroscopic fiber bundles down to atomic-scale lattice features. Available modalities span polarizing microscopy for macroscopic assessment to scanning tunneling microscopy for atomic-resolution characterization. Beyond visualizing surface and internal morphology, these methods enable quantitative correlation of orientational parameters with processing conditions, yielding mechanistic insight into ordering processes and supporting process optimization.

#### 3.4.1. Three-Dimensional Structural Imaging

X-ray computed tomography (XCT) overcomes the spatial limitations of conventional two-dimensional (2D) imaging via three-dimensional (3D) reconstruction. Its non-destructive character and volumetric information content make it suitable for investigating spatial orientational variations without compromising fiber integrity. For PAN-based materials, XCT is particularly valuable for analyzing orientation distributions within fiber tows and composite preforms.

**Figure 14:**
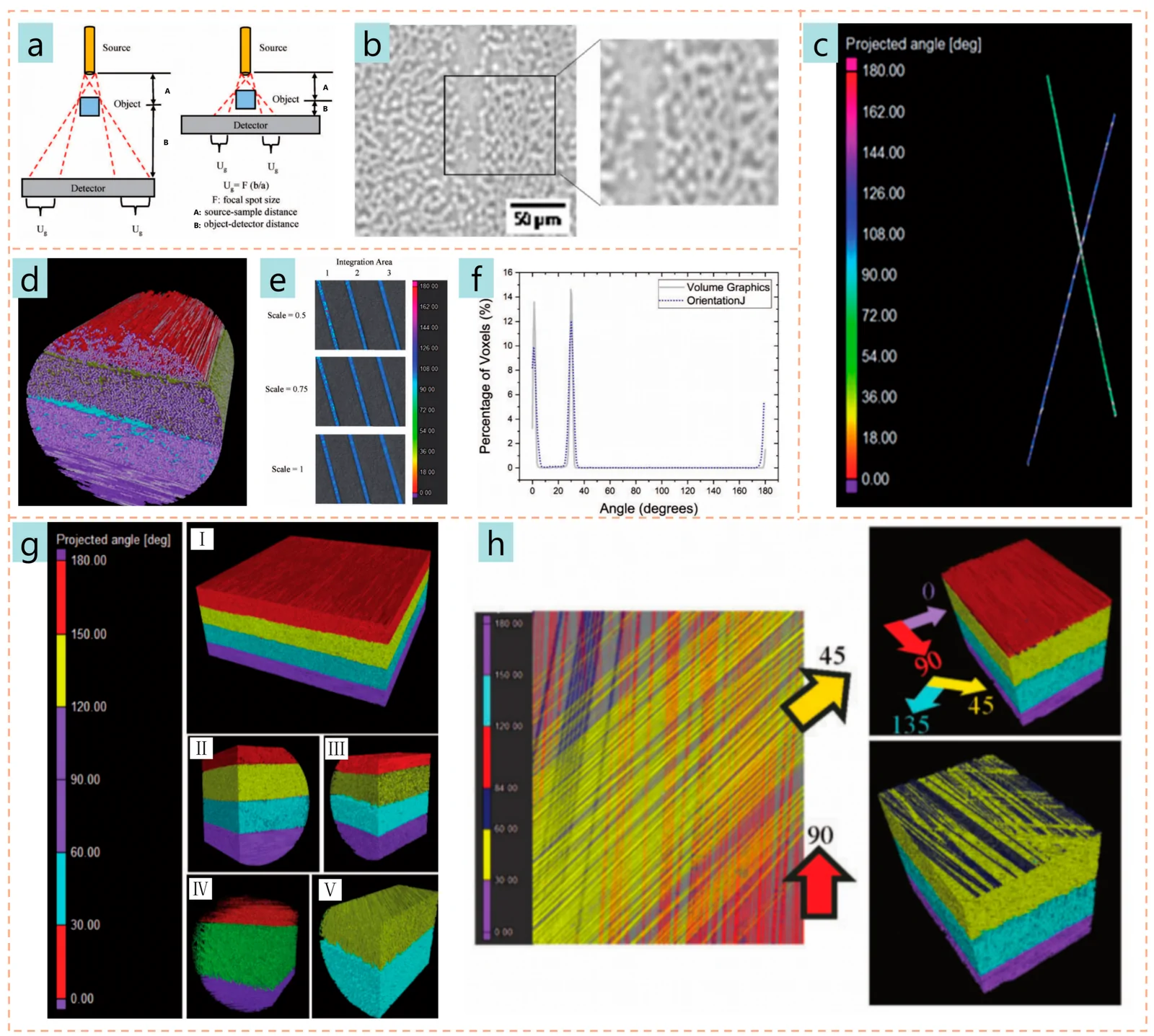
(**a**) Geometric unsharpness (Ug) as a function of object-to-detector spacing; (**b**) cross-sectional image at 1.99 μm voxel size; (**c**) excessive analysis scale produces no orientation data at fiber edges; (**d**) excessive integration area introduces boundary errors; (**e**) influence of scale and integration area on single-fiber orientation analysis; (**f**) histogram comparison of orientation results from optical microscopy and XCT; (**g**) orientation analysis of quasi-isotropic laminate at different scan settings: (**I**) 1.99 μm voxel size with the 0.4× objective lens, (**II**) 0.74 μm voxel size with the 4× objective lens, (**III**) 0.75 μm voxel size with the 20× objective lens, (**IV**) 0.38 μm voxel size with the 20× objective lens, and (**V**) 0.38 μm voxel size with the 40× objective lens; (**h**) interlaminar fiber misalignment captured at 20× objective with 0.75 μm voxel size [83].

Sietins et al. [83] established a quantitative framework for fiber orientation analysis using X-ray micro-CT, tested on two sample systems: a cross-plied AS4 carbon fiber model and a quasi-isotropic laminate with a 0°/+45°/−45°/90° layup. CT images with controlled voxel size and geometric unsharpness (Ug) were acquired by adjusting the objective-detector distance. Figure 14a shows that geometric unsharpness decreases with shorter object-to-detector distances, improving edge resolution. Figure 14b presents a cross-sectional image at 1.99 μm voxel size; given the nominal diameter of AS4 fibers at approximately 5.2 μm, this voxel size resolves the cross-section with fewer than 3 pixels, insufficient for accurate boundary delineation. A voxel size below 1 μm is therefore recommended. Figure 14c demonstrates that excessive analysis scales fail to extract edge orientation data, while Figure 14d shows that overly large integration areas introduce boundary errors. Figure 14e depicts orientation results of a single fiber under different scale-integration area combinations. The histogram comparison in Figure 14f shows excellent consistency between XCT and optical microscopy data, validating quantitative accuracy. Figure 14g presents laminate orientation results under different scan settings. Setting I at 1.99 μm voxel size with 0.4× objective captures global orientation but fails to resolve interlaminar misalignment. Settings II through V, with higher resolution at ≤0.75 μm voxel size, enable clear differentiation of interlaminar structures at reduced field of view. Figure 14h shows that the 20× objective with 0.75 μm voxel size effectively resolves interlaminar fiber misalignment, a critical feature for composite integrity evaluation.

#### 3.4.2. Mesoscopic Structural Imaging

Scanning electron microscopy (SEM) provides nanoscale resolution for visualizing fibril and microfibril arrangement at fiber surfaces, internal interfaces, and cross-sections via secondary electron detection. Its ability to capture multi-dimensional morphological features makes it useful for correlating processing-induced microfibril orientation with macroscopic fiber performance.

**Figure 15:**
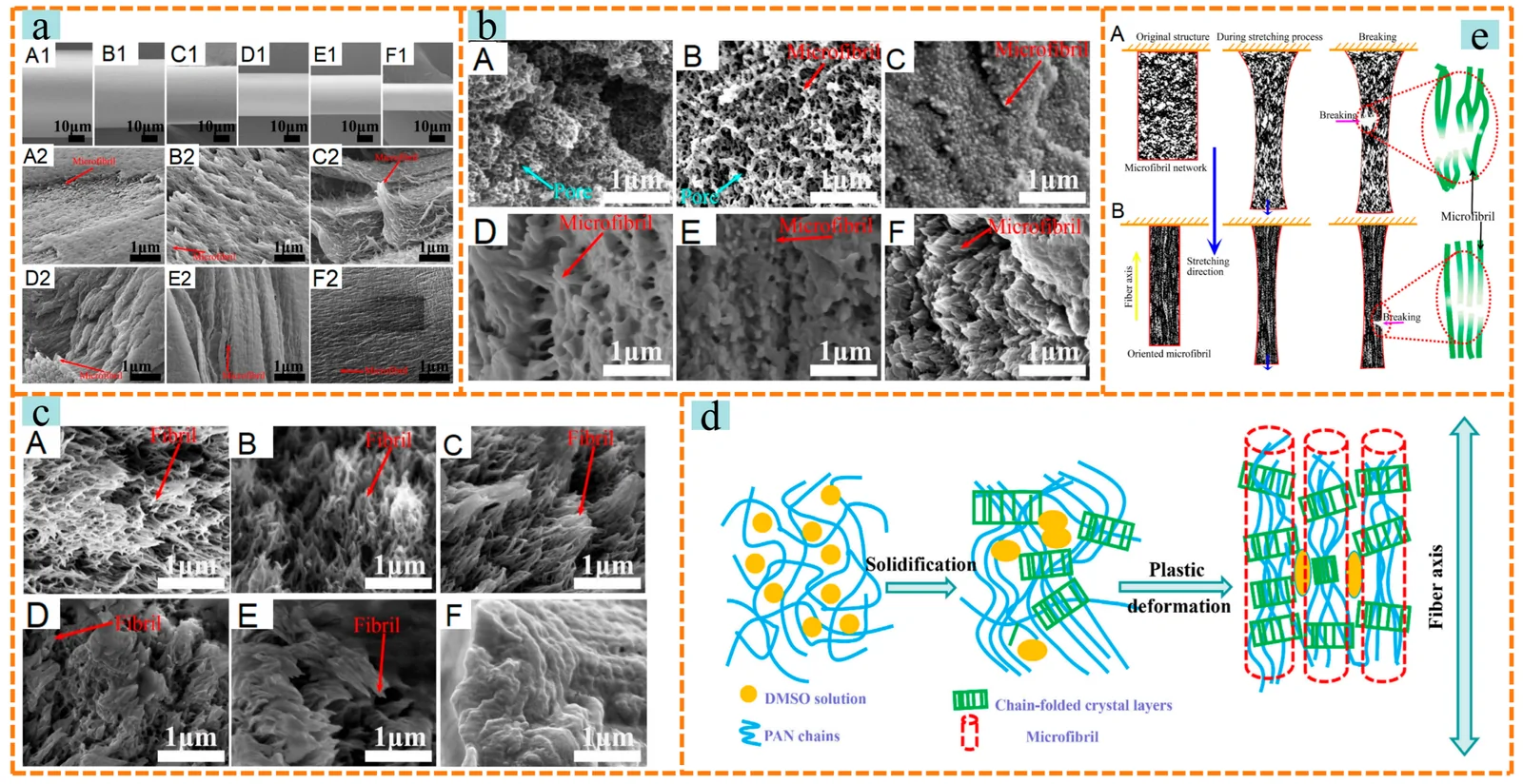
(**a**) Surface morphology of PAN fiber at six wet-stretching stages P1–P6: (**A1**–**F1**) untreated surfaces; (**A2**–**F2**) surfaces after ultrasonic etching; (**b**) cryo-fractured cross-sections at stages (**A**–**F**); (**c**) tensile fracture cross-sections at stages (**A**–**F**); (**d**) schematic of microfibril evolution during wet stretching; (**e**) (**A**,**B**) schematic of microfibril transformation during fracture [84].

Gao et al. [84] characterized surface, cross-sectional, and fracture morphologies of PAN fibers across six wet-stretching stages (P1–P6) using SEM. As-prepared fiber surfaces are smooth and free of distinct defects, and fiber diameter decreases from 53 μm at P1 to 17 μm at P6 with increasing draw ratio, confirming substantial fiber thinning by wet stretching (Figure 15a(A1–F1)). After ultrasonic etching, microfibrillar structures are exposed: microfibrils at P1 are loose and randomly oriented, whereas those at P4–P6 progressively align along the fiber axis with increased aspect ratios, demonstrating that stretching drives both microfibril orientation and densification (Figure 15a(A2–F2)).

Cryo-fractured cross-sections reveal clear structural transitions (Figure 15b(A–F)). P1–P2 exhibit a porous network with no discernible microfibril orientation; P3–P6 display well-defined microfibril bundles. Microfibril pull-out appears at P4 and P6, indicating that stretching induces microfibril aggregation into bundles, axial alignment, and enhanced interfibrillar adhesion. Tensile fracture surfaces further reflect orientational differences (Figure 15c(A–F)). P1–P3 show extensive pull-out of individual microfibrils, consistent with weak interfibrillar bonding and low orientational order. P5–P6 exhibit flatter fracture surfaces with minimal pull-out, confirming that microfibrils form tightly bonded bundles with improved alignment and structural integrity.

The microfibril evolution schematic in Figure 15d clarifies that microfibril packing and ordering govern mechanical properties and fracture behavior. At the early spinning stage, the porous microfibrillar network consists of randomly distributed microfibrils with very low orientation (Figure 15e(A)). Under tensile loading, the network first aligns along the stretching direction, accompanied by microfibril sliding and elongation. After stretching, microfibrils are highly aligned along the fiber axis with enhanced orientational order (Figure 15e(B)). Following densification, microfibrils exhibit high packing density, with regularly arranged crystalline layers alternating with amorphous regions to form a dense composite structure that optimizes both orientation uniformity and structural integrity.

#### 3.4.3. Crystallographic Orientation Imaging

Transmission electron microscopy (TEM) and selected-area electron diffraction (SAED) are standard tools for characterizing crystalline orientation and its spatial distribution in PAN-based fibers at the nanoscale.

**Figure 16:**
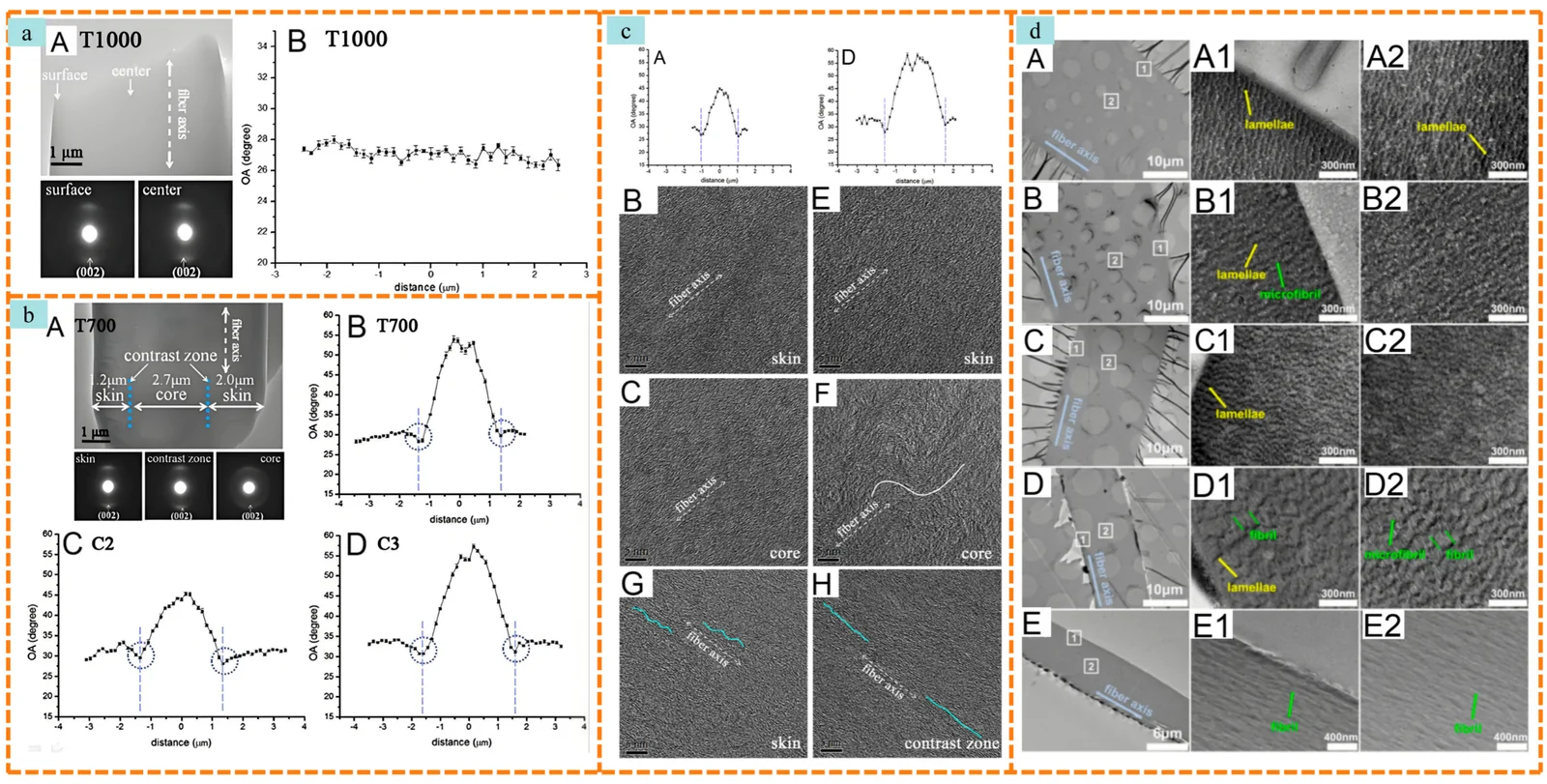
(**a**) (**A**,**B**) Longitudinal morphology of T1000 with SAED patterns from surface and core regions and corresponding OA distribution; (**b**) (**A**–**D**) longitudinal morphology of T700 with SAED patterns from skin, contrast zone and core regions and corresponding OA distribution; (**c**) (**A**–**H**) OA plots and HRTEM images for three samples: sample 1 (OA distribution, skin microstructure (**b**), core microstructure), sample 2 (OA distribution, skin, core), sample 3 (skin HRTEM, contrast zone HRTEM) [73]; (**d**) (**A**–**E**) HRTEM morphology of longitudinal ultrathin sections of P1-P5 fibers: (**A1**–**E1**) skin region; (**A2**–**E2**) core region [30].

Guo et al. [73] investigated the continuous variation in orientation angle (OA) across longitudinal sections of high-strength PAN-based carbon fibers via TEM. T1000 fibers exhibit no distinct contrast zone at the surface, and SAED patterns show no measurable difference in the (002) arc between surface and central regions. The OA distribution of T1000 is approximately flat with fluctuations below 3°, indicating homogeneous orientation without a resolvable orientation inflection point (OIP) (Figure 16a). By contrast, T700 fibers exhibit two dark contrast zones separating skin and core regions (marked by dashed lines). The angular spread of the (002) arc in the core is broader than in the skin and contrast zones. The OA curve for T700 rises gradually through the skin, decreases continuously to a minimum at the contrast zone (forming the OIP), and then rises rapidly and nonlinearly into the core (Figure 16b).

Sample 1 shows an OA difference of 14° between skin (30°) and core (44°), with basal planes in the core slightly more crumpled than in the skin, consistent with lower core orientation (Figure 16c(A–C)). For sample 2, skin OA is approximately 33° with basal planes preferentially aligned along the fiber axis; near the core center, substantial misorientation appears with OA ≈ 57° and increased crumpling (Figure 16c(D–F)). For sample 3, crystallites in the contrast zone are larger and less crumpled than those in the skin, accounting for OIP formation (Figure 16c(G,H)). The contrast zone thus represents a region of strong preferred layer-plane orientation, whereas basal planes near the core center are largely randomly oriented.

He et al. [30] investigated skin-core structure and orientational evolution in PAN precursor fibers during dry-jet wet spinning post-processing using high-resolution TEM (HRTEM). At the coagulation bath stage (P1), the skin exhibits a relatively regular lamellar structure, whereas the core is loose with randomly entangled chains and very low orientation (Figure 16d(A)). During water washing (P2), fiber swelling loosens the lamellae, and entangled chains begin to relax and undergo preliminary alignment along the stretching direction, producing an initial skin-core orientation difference (Figure 16d(B)). In boiling water stretching (P3), transverse lamellae in P3 fibers are disrupted and reorganized under tensile force and temperature, and chains align directionally to form longitudinal microfibrillar structures, marking the onset of axial microfibril orientation (Figure 16d(C)). At the densification stage (P4), high-temperature crystallization and tensile stress drive tight stacking of oriented microfibrils into crystalline layers, improving chain arrangement regularity; microfibrils in the skin increase in size and packing density with further improved orientation (Figure 16d(D)). During heat setting (P5), steam stretching and thermal annealing homogenize microfibrillar structures in skin and core, achieving highly uniform crystal orientation with greatly diminished skin-core differences and maximum overall orientation degree (Figure 16d(E)).

#### 3.4.4. Nanoscale Imaging

Atomic force microscopy (AFM) and scanning tunneling microscopy (STM) provide nanometer to atomic-scale resolution for surface characterization of PAN fibers. These techniques simultaneously capture topographical, mechanical, and electronic properties, offering insight into orientational ordering mechanisms not accessible by conventional microscopy.

**Figure 17:**
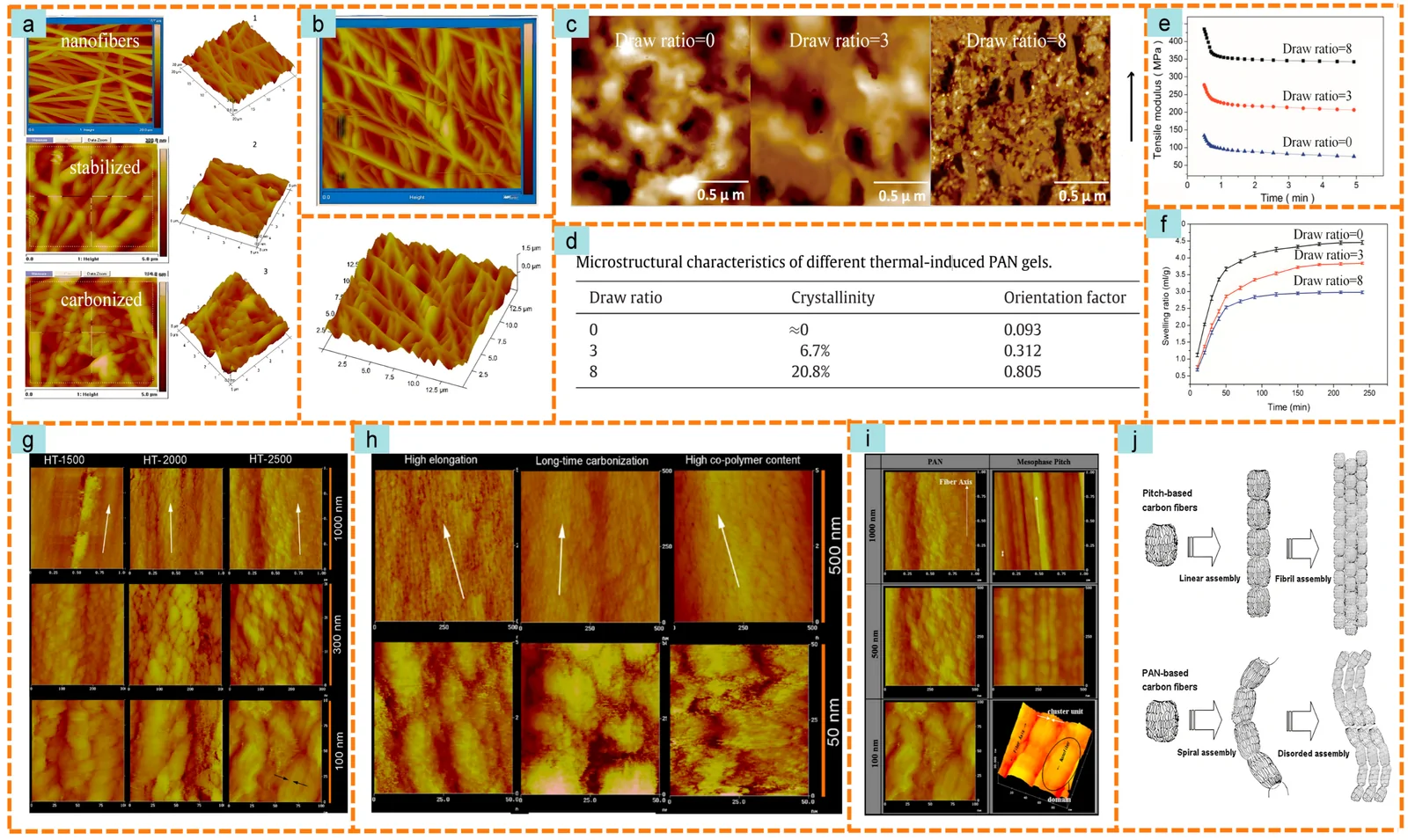
(**a**) 2D and 3D AFM images of PAN copolymer nanofibers mat at 9 wt% in DMF; (**b**) AFM images of carbonized PAN-CNT composites from 3 wt% CNT concentration [85]; (**c**) AFM micrographs of thermally induced PAN gels at different draw ratios; (**d**) microstructural characteristics; (**e**) tensile modulus versus time; (**f**) swelling ratio versus time [86]; (**g**) STM images of HT-1500, HT-2000, and HT-2500 fibers; (**h**) STM images of carbon fibers prepared under high elongation, long-time carbonization, and high copolymer content; (**i**) STM images of PAN-based and mesophase pitch-based carbon fibers; (**j**) schematic domain arrangement models [75].

Kaur et al. [85] investigated the effect of processing stage and CNT incorporation on PAN nanofiber orientation via 2D and 3D AFM. As-spun PAN nanofibers are randomly entangled with no axial preference, and surfaces exhibit high, unoriented roughness with no directional correlation between adjacent fibers (Figure 17a). After stabilization, cyclization-induced shrinkage reduces fiber diameter and slightly decreases surface roughness without producing new preferential orientation. Carbonization produces further diameter reduction and smoother surfaces, but random entanglement persists. CNT incorporation modulates fiber diameter and surface undulations but does not disrupt the random orientational character (Figure 17b).

Tan et al. [86] observed the anisotropic evolution of microfibrillar networks in uniaxially stretched PAN gels using tapping-mode AFM. At zero draw ratio, PAN chains form an isotropic fractal network with no orientational trend. At a draw ratio of 3, the network densifies and chains align moderately along the stretching direction, with the orientation factor increasing to 0.312. At a draw ratio of 8, microphase separation produces PAN-rich and PAN-poor domains, the structure compacts, and oriented microstructures are clearly visible with the orientation factor rising to 0.805 (Figure 17c,d). Stress relaxation behavior of PAN gels at three draw ratios shows tensile modulus increasing with draw ratio (Figure 17e). Swelling kinetics in DMSO show the maximum swelling ratio decreasing significantly with draw ratio, consistent with densification via pore volume reduction (Figure 17f).

Li et al. [75] characterized mesodomain structures on longitudinal carbon fiber surfaces via STM. Mesodomain size increases and boundary definition improves with heat treatment temperature; at 2500 °C, strip-like structures approximately 7 nm in diameter appear, demonstrating that high-temperature graphitization improves orientational order (Figure 17g). High elongation produces distinctly elongated mesodomains with the strongest directional spiral alignment and highest tensile strength. Long-duration carbonization yields moderate-sized mesodomains with intermediate packing density and moderate strength. High comonomer content produces large, irregular mesodomains with disordered spiral alignment and the lowest strength (Figure 17h). Processing parameters thus tailor mesodomain morphology to regulate orientational order and determine fiber performance.

PAN-based and mesophase pitch-based carbon fibers exhibit distinct orientational modes (Figure 17i). Pitch-based fibers show uniformly granular mesodomains, approximately 80 nm long and 70 nm in diameter, arranged linearly along the fiber axis with high strip linearity. PAN-based fibers show irregularly twisted mesodomains arranged in a spiral winding pattern with poor linearity. These two orientational modes account for the high modulus of pitch-based fibers and the high strength of PAN-based fibers. Corresponding mesodomain structure models are proposed in Figure 17j, clarifying the core mechanisms of orientational ordering.

### 3.5. In Situ Characterization Techniques

In situ characterization techniques are currently predominantly deployed in laboratory fundamental research. The translation of these high-resolution dynamic methods to industrial-scale production lines remains an ongoing technical challenge. These methods capture the dynamic structural evolution of PAN fibers under processing-relevant conditions, complementing the spatiotemporal constraints of ex situ analysis. These techniques resolve transient changes in molecular chain alignment during spinning, drawing, pre-oxidation, and carbonization, and establish time-resolved correlations between processing parameters, orientational structures, and macroscopic performance. Based on the underlying measurement principle and accessible length scale, they fall into three categories: in situ scattering, in situ spectroscopy, and in situ mechanics-structure coupling.

#### 3.5.1. In Situ X-Ray Scattering

In situ X-ray scattering, with millisecond temporal resolution and multiscale structural sensitivity, provides a powerful approach for investigating dynamic orientational evolution in PAN fibers. By coupling synchrotron WAXD and SAXS with controlled environmental stages such as pre-oxidation and carbonization, this method enables time-resolved probing of crystalline orientation and mesostructural evolution under combined temperature and stress fields.

**Figure 18:**
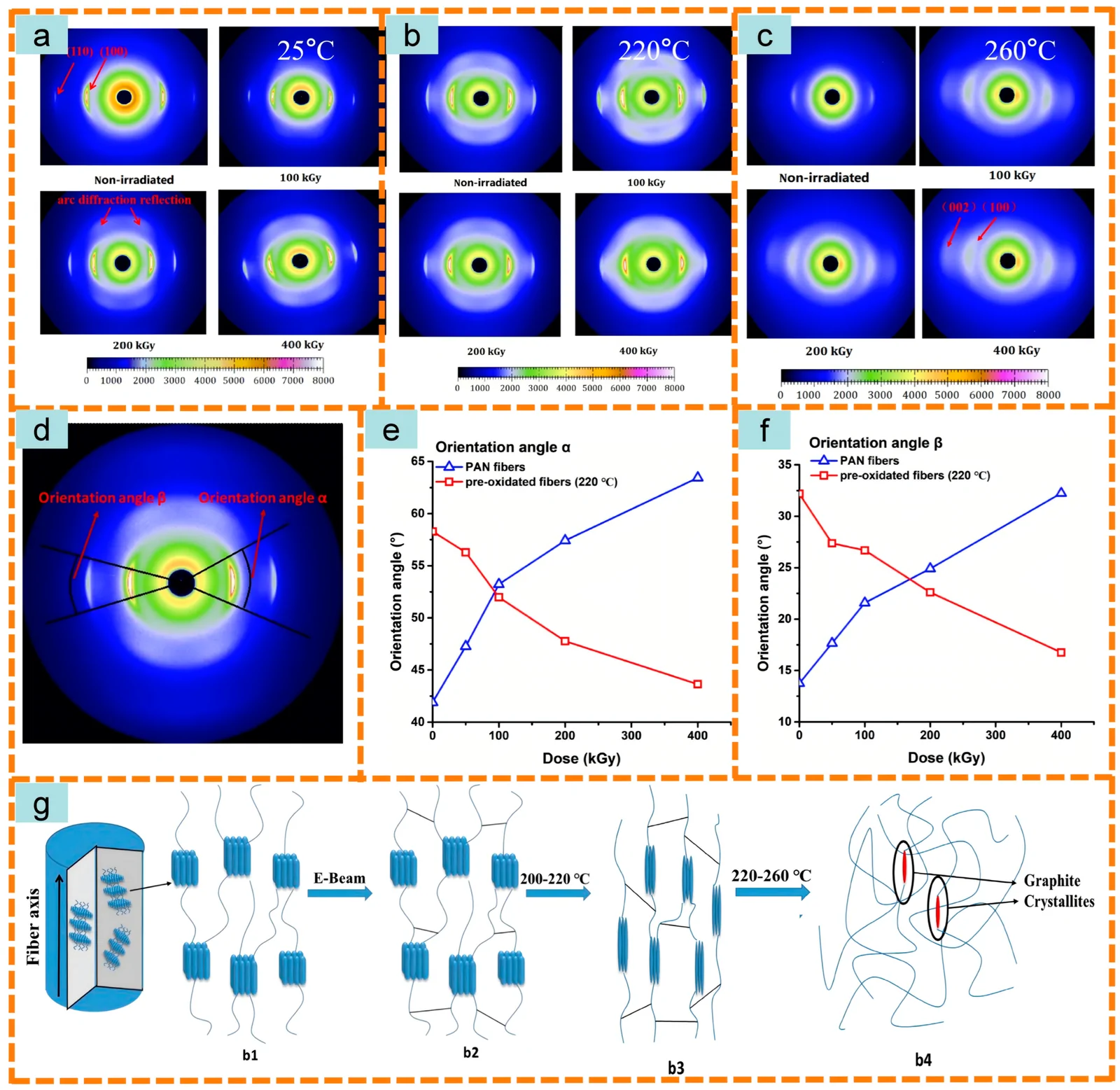
2D WAXD patterns of irradiated and non-irradiated PAN fibers during pre-oxidation at (**a**) 25 °C, (**b**) 220 °C, and (**c**) 260 °C; (**d**) diffraction arc of (100) and (110) crystal plane; (**e**,**f**) orientation angle α and β distributions along the fiber axis; (**g**) schematic of crystallite evolution during pre-oxidation for irradiated fibers [87].

Zhang et al. [87] examined the effect of γ-ray irradiation pretreatment on orientational evolution during PAN fiber pre-oxidation using in situ 2D WAXD. At 25 °C, unirradiated PAN fibers show intense (100) and (110) crystalline reflections aligned along the fiber meridian, confirming high initial molecular chain orientation (Figure 18a). At irradiation doses ≥200 kGy, diffraction rings evolve into four symmetric arcs, attributed to radiation-induced chain scission and amorphous phase formation. During pre-oxidation at 220–260 °C, diffraction arcs from irradiated samples progressively broaden and weaken (Figure 18b,c). At 400 kGy, a new (002) peak corresponding to nascent graphitic structures emerges, indicating that irradiation accelerates both orientational structural disruption and cyclization kinetics. Orientation angles decrease in the early pre-oxidation stage, reflecting chain disordering, but irradiated samples show partial orientation recovery in the late thermal treatment stage (Figure 18d–f). This recovery is associated with radiation-induced crosslinking that restricts chain mobility, as illustrated schematically in Figure 18g.

**Figure 19:**
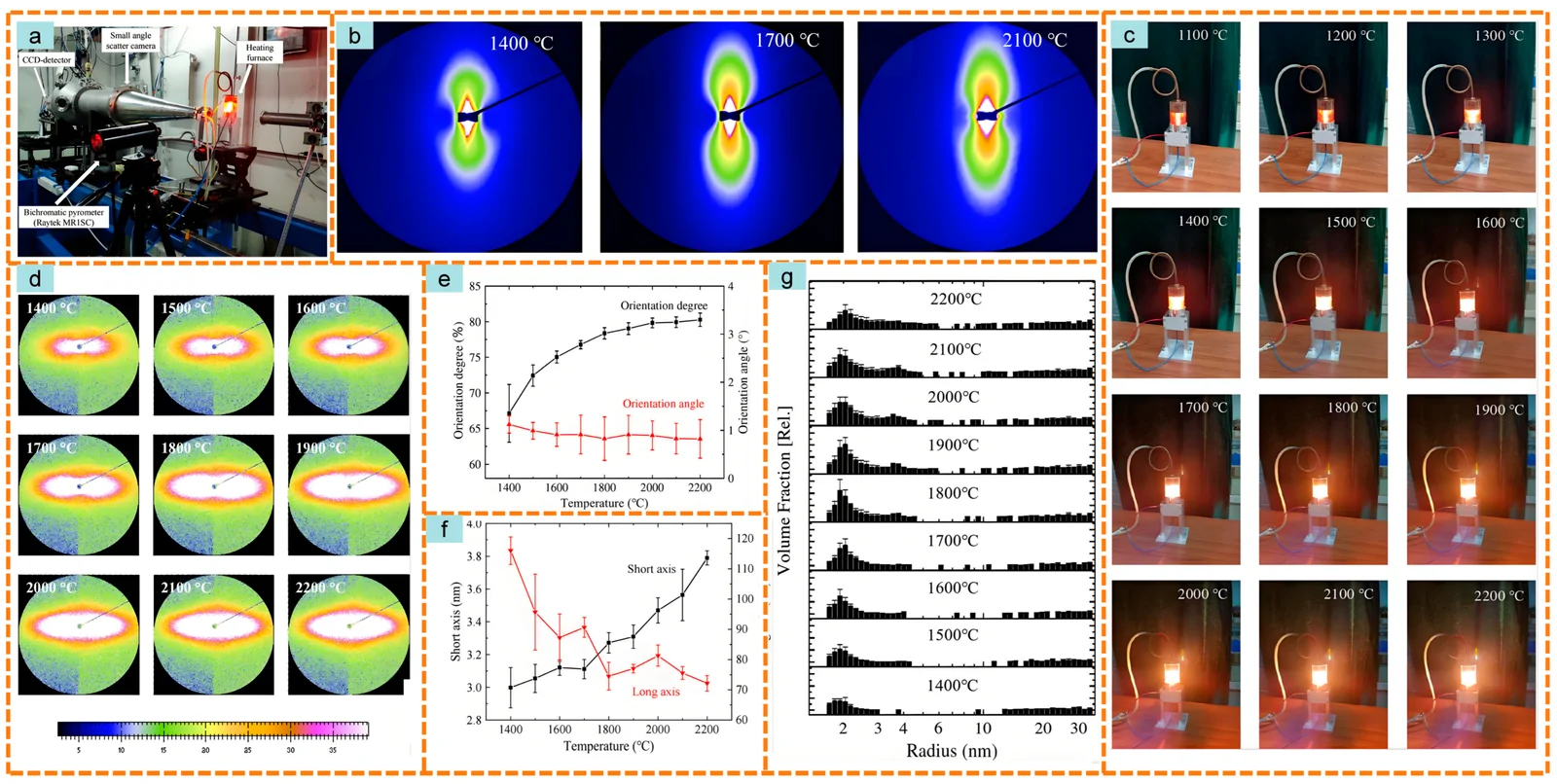
(**a**) in situ SAXS setup for T700 carbon fiber graphitization at BSRF beamline 1W2A; (**b**) 2D SAXS patterns of T700 carbon fibers at 1400 °C, 1700 °C, and 2100 °C [88]; (**c**) fiber photographs at different graphitization temperatures; (**d**) SAXS images at different temperatures; (**e**) orientation angle and degree evolution; (**f**) Changes in long axis and short axis of pores; (**g**) pore size distribution from equatorial scattering [89].

Xiao et al. [88,89] constructed an in situ orientational analysis platform by integrating a custom-built direct-resistance graphitization furnace with the SAXS station at beamline 1W2A of the Beijing Synchrotron Radiation Facility (Figure 19a). The platform enables dynamic tracking of microvoid orientation during carbon fiber graphitization (Figure 19b,c). In situ monitoring over 1400–2200 °C reveals that internal microvoid morphology evolves from fan-shaped or dumbbell-shaped to elliptical with increasing temperature, indicating enhanced preferential alignment of acicular microvoids along the fiber axis (Figure 19d). The axial orientation degree increases rapidly from 0.65 at 1400 °C to 0.78 at 1800 °C, and reaches 0.81 at 2200 °C (Figure 19e). The pore size distribution broadens with an increasing fraction of large pores as temperature rises (Figure 19f,g). This work confirms the synergistic mechanism of microcrystal orientation-induced microvoid alignment during graphitization, and establishes a quantitative correlation between microvoid orientation degree and graphitization extent.

Combined in situ WAXD and SAXS measurements thus provide direct dynamic evidence for both inheritance and modification of hierarchical structures during thermal treatment. WAXD results confirm that the preferred axial orientation of crystalline domains is retained and enhanced as temperature rises, while crystallite size and in-plane order are continuously modified. SAXS results further show that mesoscale microvoids preserve their axial alignment while undergoing progressive shape evolution, bridging the gap between precursor structural features and final carbon fiber properties.

#### 3.5.2. In Situ Spectroscopy

In situ spectroscopy probes molecular-level orientational dynamics by monitoring vibrational mode evolution of characteristic functional groups during thermal processing.

**Figure 20:**
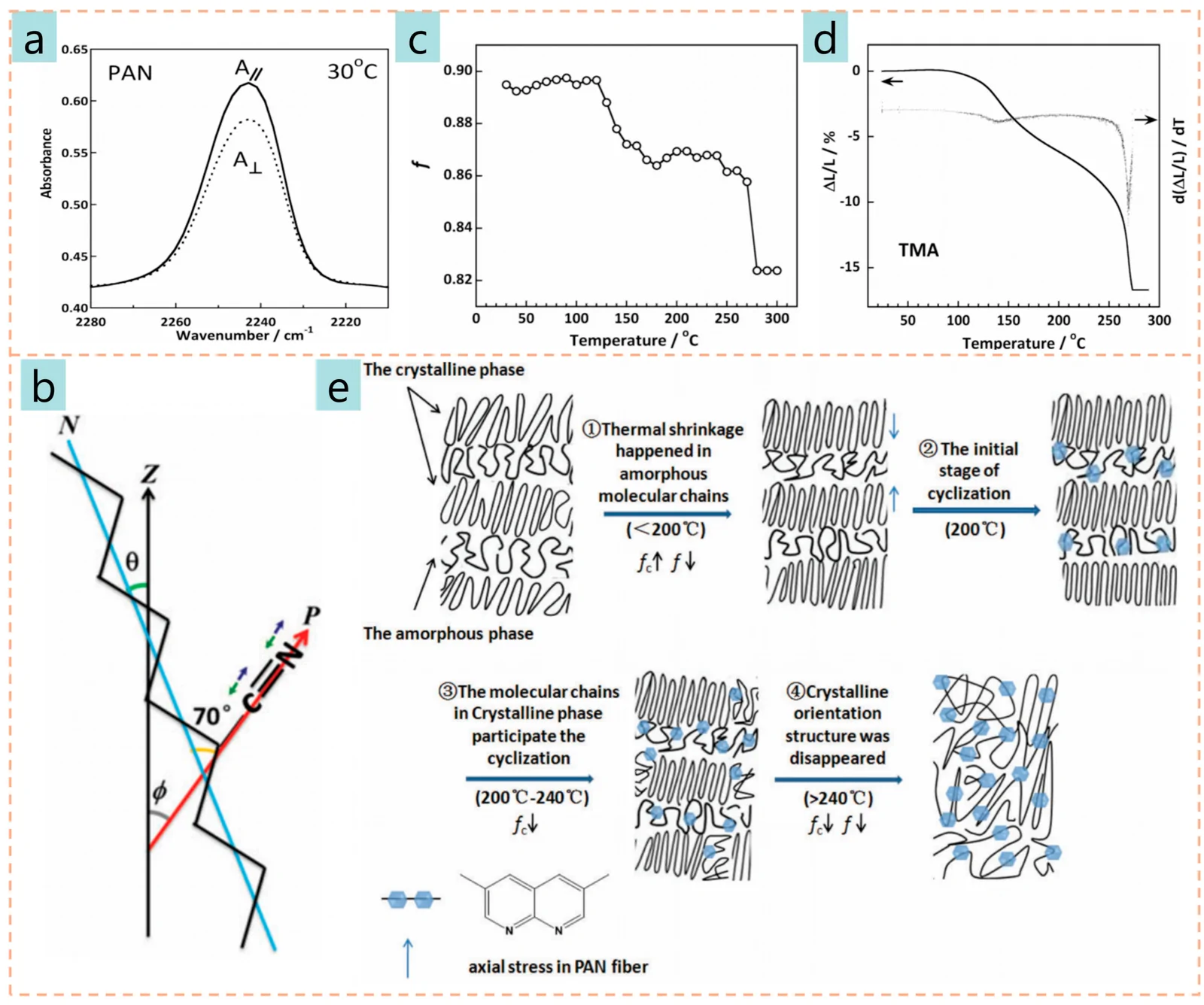
(**a**) Polarized IR spectra of ʋ_C≡N_ band for PAN precursors; (**b**) schematic of molecular chain orientation relative to fiber axis; (**c**) orientation factor f versus stabilization temperature; (**d**) thermomechanical curve of PAN precursor; (**e**) schematic of orientation structure evolution during stabilization [90].

Zhou et al. [90] investigated orientational evolution in PAN precursors during thermal stabilization using polarized infrared spectroscopy. Polarized IR spectra of the -C≡N stretching vibration show that the absorbance ratio A_‖_/A_⊥_ decreases with temperature, reflecting attenuation of preferential chain orientation (Figure 20a). Using a spatial orientation model in which the -C≡N bond forms a fixed 70° angle with the PAN main chain axis (Figure 20b), the angle θ between the chain axis and fiber axis is derived from the dichroic ratio D = A_‖_/A_⊥_, and the molecular chain orientation factor f is calculated via the Herman’s equation. The f-temperature curve in Figure 20c starts at 0.90 at 25 °C. Below 200 °C, f decreases mildly, dominated by thermal relaxation of amorphous chains. Between 200 °C and 290 °C, f declines more gradually, driven by chain conformational rearrangement and microdomain shrinkage during cyclization, eventually reaching 0.82. The initial decline in f above Tg directly reflects the thermal relaxation window described in Section 2.2.4; the subsequent plateau marks the point at which ladder crosslinking has become sufficiently dense to immobilize chain segments, after which orientation is chemically fixed. The corresponding thermomechanical curve is shown in Figure 20d. The fiber first undergoes ~5% shrinkage from thermal relaxation, followed by 10% shrinkage from chain conformational adjustment during cyclization. These temperature ranges and magnitudes correspond closely with the two-stage evolution of f, verifying the coupling between microscopic orientational evolution and macroscopic deformation. These results are integrated into a multiscale evolution model in Figure 20e, which summarizes the stepwise orientational structural evolution of PAN fibers during pre-oxidation.

#### 3.5.3. In Situ Mechanics–Structure Coupling

In situ mechanics–structure coupling integrates mechanical loading systems with structural characterization tools, enabling real-time analysis of orientational structural response under temperature-stress fields that mimic industrial processing conditions. This approach bridges macroscopic mechanical loading and microscale orientational evolution.

**Figure 21:**
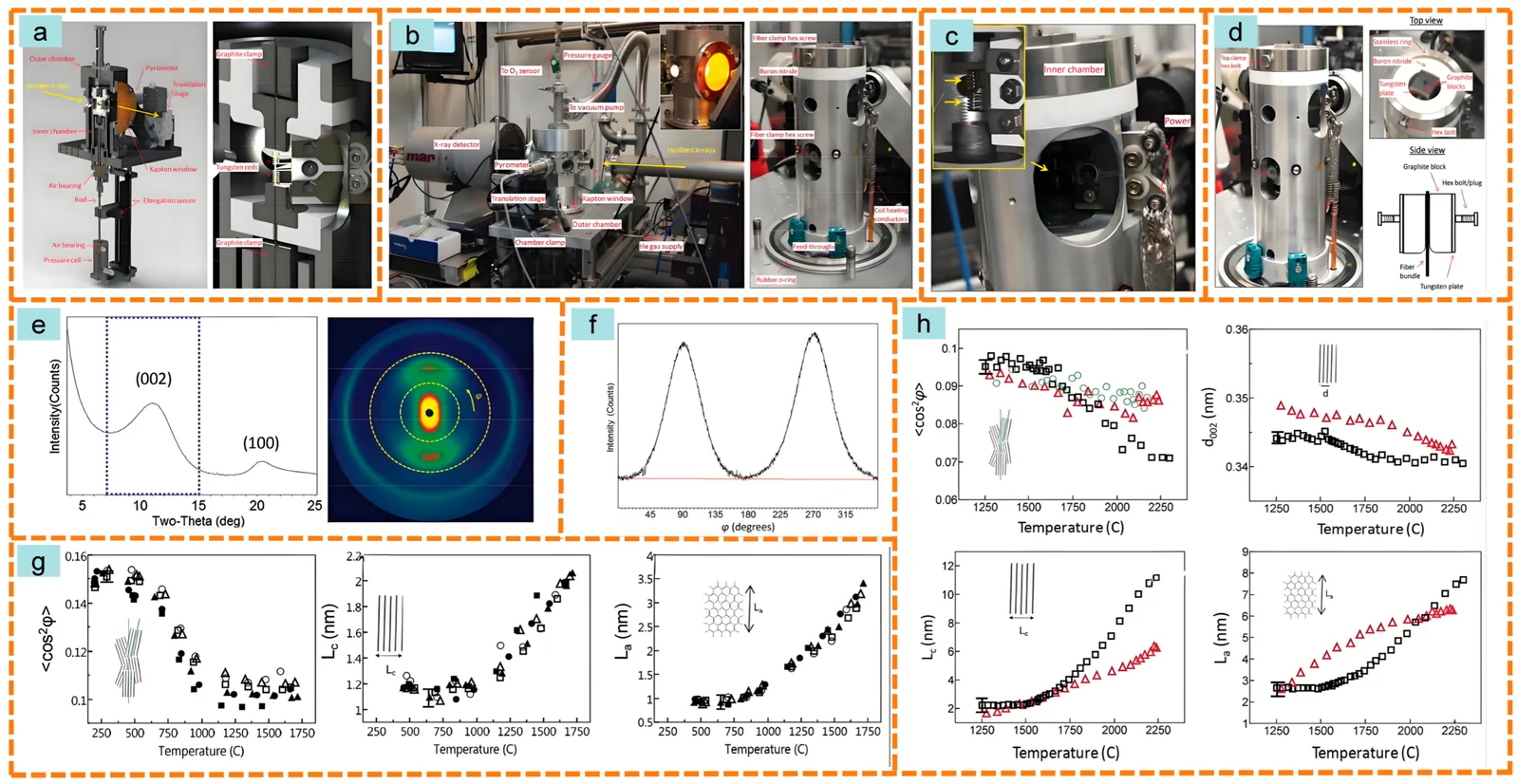
(**a**) Computer renderings of high-temperature tensile device; (**b**) device setup at APS in hutch 5ID-B; (**c**) tungsten coil fiber heating assembly; (**d**) inner chamber with fiber clamp; (**e**) radial integration of WAXD pattern showing intensity versus 2θ with 2D WAXD inset; (**f**) azimuthal intensity variation; (**g**) microstructure orientation and crystal sizes at high stress 15 MPa and low stress 3 MPa; (**h**) orientation, d-spacing, and crystal sizes at 20 MPa for T300 (red triangles), IM-7 (green circles), and P25 fibers (black squares) [91].

Behr et al. [91] developed an in situ analysis platform by integrating a high-temperature tensile stage with synchrotron WAXD (Figure 21a–d). The system employs two heating modes: tungsten coil radiative heating for non-conductive fibers, and direct resistive heating up to 2300 °C. Combined with fiber-specific tensile clamps and a vacuum chamber, it enables real-time orientation characterization over 500–2300 °C and 0–20 MPa. Radial integration (Figure 21e) and azimuthal scanning (Figure 21f) of WAXD patterns yield quantitative parameters including cos^2^φ, where φ is the angle between the graphite layer normal and the fiber axis; smaller cos^2^φ indicates higher graphite layer orientation along the fiber axis. With tungsten coil heating, orientation degree increases significantly with temperature from 500 °C to 1100 °C, with cos^2^φ decreasing from 0.15 to 0.1. Above 1100 °C, cos^2^φ reaches a plateau. Higher applied tensile stress (15 MPa versus 3 MPa) reduces the temperature required to reach target orientation but does not alter the plateau above 1100 °C or significantly affect crystallite dimensions Lc and La (Figure 21g). Direct resistive heating up to 2300 °C reveals strong precursor-type dependence (Figure 21h). For PAN-based T300 and IM-7 fibers, orientation degree shows minimal variation with temperature; cos^2^φ decreases only from 0.093 at 1250 °C to 0.089 across 1250–2250 °C. For pitch-based P25 fibers, orientation degree increases substantially above 1500 °C, with cos^2^φ dropping from 0.096 at 1250 °C to 0.071 at 2250 °C without a clear plateau. This contrast demonstrates that precursor molecular structural anisotropy is the primary factor governing orientational tunability at high temperatures.

### 3.6. Comparison and Combined Deployment of Characterization Techniques

The hierarchical, multiscale nature of orientational order in PAN fibers means that no single analytical technique can resolve all relevant length scales with sufficient accuracy. X-ray scattering, vibrational spectroscopy, optical birefringence, electron microscopy, and in situ methods each probe orientational features at characteristic dimensions. This section compares their operating principles, key performance metrics, and practical limitations, and discusses strategies for their combined application in PAN fiber orientation research.

#### 3.6.1. Technical Performance Metrics

Key performance parameters of the principal orientation characterization techniques are summarized in Table 3.

X-ray scattering methods, particularly when implemented at synchrotron sources, enable quantitative multiscale characterization of both crystalline and mesoscale orientational features. WAXD delivers crystalline orientation factors with a measurement uncertainty below ±0.02, while SAXS resolves the orientational distribution of 1–300 nm microvoids. A key strength of X-ray methods is their ability to distinguish orientational order in crystalline versus amorphous regions. Performance depends strongly on beam quality, conventional laboratory XRD systems lack dynamic monitoring capability and show insufficient sensitivity for phases below 1% volume fraction.

Polarized FTIR and Raman spectroscopy form a complementary suite of molecular-level probes. FTIR is sensitive to the orientational state of polar functional groups, particularly the cyano stretch near 2240 cm^−1^, making it well suited for assessing chain arrangement in amorphous regions. Raman spectroscopy characterizes microdomain orientation via the CH_2_ bending vibration near 1450 cm^−1^, with spatial resolution down to 1 μm. FTIR is limited by infrared spot size and cannot resolve sub-micrometer orientational heterogeneities. Raman is susceptible to fluorescence interference, typically mitigated by using a 785 nm near-infrared excitation laser to improve signal-to-noise ratio.

Optical birefringence provides a rapid route to bulk fiber orientation assessment via measurement of the refractive index difference Δn. Equipment costs are roughly one-tenth those of X-ray systems, making birefringence suitable for industrial on-line monitoring with millisecond temporal resolution. Spatial resolution is comparatively modest at approximately 1 μm. The method typically requires annealing at 80 °C for 30 min to eliminate stress-birefringence contributions. Traditional measurements suffer from sign ambiguity in the phase difference; circularly polarized light methods resolve the sign ambiguity but restrict high-precision measurements to the range −π/3 to π/3.

Microscopic imaging modalities cover length scales from the macroscopic down to the atomic level. Polarized light microscopy provides visual assessment of global orientational uniformity. SEM resolves the arrangement of 200–500 nm microfibrils. HRTEM identifies orientational features of 5–10 nm crystallites. AFM achieves atomic-scale characterization of surface topography and its correlation with orientational order. Electron microscopy requires vacuum conditions, and sample preparation can introduce structural artifacts that complicate interpretation of intrinsic orientational information.

In situ methods capture the dynamic evolution of orientational order via multi-field coupling experiments. Synchrotron-based in situ WAXD has resolved the three-stage evolution of the orientation factor during PAN fiber drawing. in situ mechanics-Raman coupling has mapped skin-core orientational gradients during pre-oxidation. The core strength of in situ approaches is their ability to establish time-resolved correlations between processing parameters and structural evolution. Practical challenges include infrared radiation interference and sample drift at temperatures above 500 °C, and large dynamic datasets often require machine learning (ML) assisted analysis.

Direct numerical comparison of orientation values across different characterization techniques is not valid, as each method probes a distinct structural level. WAXD samples only the paracrystalline sub-population (defect-free chain bundles), while polarized FTIR averages over all –C≡N dipoles including defective interfibrillar regions. The two therefore report different weighted averages and should not be numerically equated. Optical birefringence reflects the bulk-averaged orientation of all structural units, including both crystalline and amorphous regions, and its values are further affected by stress-induced birefringence in as-spun fibers. For example, the same PAN precursor may give a crystalline orientation factor of 0.86 via WAXD and a chain orientation factor of 0.70 via polarized FTIR. This intrinsic methodological difference explains much of the numerical scatter in orientation values across the literature, and readers should take the measurement principle into account when comparing results from different studies.

#### 3.6.2. Advantages and Limitations

X-ray scattering excels at quantitative multiscale orientational analysis. WAXD calculates the crystalline orientation factor from the azimuthal distribution of the (100) diffraction peak, while SAXS derives microvoid aspect ratio and orientation angle from equatorial scattering spot morphology. In PAN pre-oxidation studies, synchrotron X-ray scattering has simultaneously monitored crystalline orientation relaxation and microvoid directional alignment, clarifying the synergy between chemical crosslinking and structural ordering. Several constraints limit broader applicability: synchrotron beamtime requires competitive application and is inaccessible to most laboratories; conventional laboratory XRD has minute-scale temporal resolution and cannot capture transient orientational changes; and sensitivity to amorphous-phase orientation is limited, necessitating combination with spectroscopic techniques. Laboratory diffractometers cannot support real-time on-line monitoring on production lines, and synchrotron beamtime is restricted to fundamental research; neither is suitable for high-throughput industrial quality control.

Spectroscopic methods offer chemically specific probes of orientational order. Polarized FTIR quantifies pre-oxidation progress via cyano group dichroism: as cyclization degree increases from 0 to 0.8, the cyano orientation factor decreases by 43%. Raman mapping has shown that orientational order in the skin layer of steam-stretched PAN fibers is 30–40% higher than in the core, providing direct evidence for skin-core structural heterogeneity. FTIR spatial resolution is limited by spot size to ~5 μm, preventing detection of sub-micrometer domain heterogeneities. Raman signal intensity is comparatively low, requiring integration times of 10–60 s, and thermal radiation from hot samples substantially degrades the signal-to-noise ratio. Bench-top systems are used predominantly for offline laboratory analysis. Fiber-optic probe configurations can be adapted for on-line monitoring, but their performance is limited by interference from industrial environmental heat and dust.

Optical birefringence occupies a unique application in industrial quality control. On-line monitoring studies show that increasing winding speed from 500 to 1500 m/min raises birefringence linearly from 0.018 to 0.028, with a strong positive correlation to tensile strength (R^2^ = 0.96). This enables rapid feedback on process parameter effects. Limitations include an inability to separate crystalline and amorphous contributions to the birefringence signal, stress birefringence contributions introducing measurement deviations of 10–15%, and the need for cross-sectional area calibration for variable-diameter fibers. Novel phase-difference instruments integrating circularly polarized light and phase-locked detection have improved accuracy to ±0.001, partially overcoming traditional constraints. With millisecond response speed, low equipment cost, and compatibility with continuous fiber tow lines, optical birefringence is currently the most widely deployed technique for real-time orientation monitoring in industrial PAN precursor production.

Microscopic imaging delivers direct morphological corroboration of orientational structures. SEM confirms that as-spun PAN fibers consist of 200–500 nm microfibrils; after drawing, microfibril diameter reduces to 100–200 nm with more regular axial alignment. TEM reveals that 5–10 nm crystallites adopt preferential alignment along the fiber axis. Limitations include the vacuum requirement of SEM and TEM, which precludes dynamic monitoring; the limited scan range of AFM (typically <10 μm) reduces statistical representativeness; and sample preparation such as ultramicrotomy can damage intrinsic orientational structures. All modalities are strictly ex situ laboratory techniques requiring dedicated sample preparation, and cannot be implemented for in-line production monitoring.

In situ characterization is uniquely powerful for uncovering dynamic orientational mechanisms. in situ mechanics-Raman coupling experiments show that below the yield point (strain < 5%), orientational evolution is dominated by molecular chain slippage with an orientation growth rate of 0.02 per % strain. After yielding, the growth rate increases to 0.05 per % strain, accompanied by crystallite rotation. Major challenges include precise multi-field coupling control requiring temperature stability within ±1 °C, infrared background interference in high-temperature environments requiring specialized filters, and large dynamic dataset analysis requiring advanced algorithms such as ML for rapid orientation distribution function extraction.

As a general caveat, all quantitative orientational metrics reported in the literature are strongly dependent on sample preparation history, test conditions, and calculation models. Direct numerical comparison across studies should only be performed with matched material systems, processing protocols, and characterization methodologies to avoid misleading conclusions.

#### 3.6.3. Combined Strategies and Objective-Driven Selection

The inherent limitations of individual techniques motivate multi-modal characterization strategies. Coupling WAXD with polarized FTIR enables complementary sampling: WAXD reports the orientation of defect-free paracrystalline bundles, while FTIR averages over the broader chain population including defective interfibrillar regions. In PAN fiber research, the ratio of the crystalline orientation factor from WAXD to the amorphous orientation factor from FTIR remains stable at 1.2–1.3, providing a quantitative metric for assessing structural integrity. Combining optical birefringence with SEM links macroscopic orientational uniformity to microscopic microfibril arrangement; a birefringence standard deviation exceeding 0.005 correlates with increased dispersion of microfibril orientation angles, providing precise feedback for process optimization.

Cross-coupled in situ techniques deliver enhanced mechanistic insight. Synchrotron-based in situ XRD combined with Raman spectroscopy tracks crystalline orientation evolution and cyano group vibrational responses during thermal drawing, revealing a time lag of ~200 ms between molecular chain motion and crystalline reorganization. This finding clarifies microscale orientational regulation mechanisms. In situ electron backscatter diffraction with a high-temperature stage suffers from infrared interference, but modification with a specialized fluorescent screen enables continuous orientation tracking at 1000 °C, providing a new approach for investigating orientational behavior during graphitization.

Optimal technique selection is guided by the specific research objective. For process optimization and quality control, optical birefringence is preferred for on-line real-time monitoring due to its speed and low cost. For mechanistic studies of dynamic orientational regulation, in situ X-ray techniques are the method of choice. For structure–property investigations, a combination of spectroscopy and X-ray scattering is recommended to concurrently acquire crystalline and amorphous domain orientational information. Refined microstructural analysis relies on SEM and TEM, with AFM supplementing nanoscale surface characterization. In situ techniques are indispensable for dynamic orientational evolution studies, and a multiscale real-time monitoring platform based on synchrotron radiation is recommended for fundamental mechanistic research.

For fundamental laboratory research, a multi-modal combination of synchrotron-based in situ X-ray scattering and polarized Raman spectroscopy is recommended to resolve dynamic orientational evolution across multiple length scales and support mechanistic model development. For industrial on-line quality control, a two-tier strategy is proposed. Optical birefringence is the primary real-time monitoring tool for continuous process feedback, paired with periodic off-line WAXD and SEM sampling for in-depth structural verification and calibration.

## 4. Challenges and Theoretical Limitations

### 4.1. Outstanding Fundamental Issues

#### 4.1.1. Cross-Scale Integration Gaps in Multiscale Models

Orientational order in PAN fibers manifests across hierarchical length scales, from molecular chain alignment at the nanometer scale to skin-core heterogeneity at the micrometer level. Most existing theoretical frameworks operate within individual length scales and lack robust coupling across regimes. Molecular dynamics (MD) simulations resolve short-range intermolecular interactions governing chain alignment and have, for example, clarified how cyano dipole interactions drive conformational evolution of individual PAN chains [92]. However, MD simulations do not scale to mesoscopic dimensions. No quantitative model yet connects local cyano group configurations to crystalline nucleation mechanisms, which prevents molecular-scale simulation insights from directly guiding industrial spinning parameter optimization.

Continuum models, including Doi–Edwards theory, describe segmental stretching under macroscopic flow fields [93]. Such continuum descriptions typically neglect the modulating influence of comonomer concentration gradients, such as itaconic acid, on interchain entanglement networks. This simplification introduces predictive deviations, particularly for the correlation between coagulation bath drawing-induced chain alignment and macroscopic tensile strength in wet spinning.

Finite element analysis (FEA) has been widely used to predict the macroscopic mechanical response of PAN fibers [94]. However, standard FEA do not adequately resolve the quantitative link between coagulation-bath draw-induced chain alignment and the resulting macroscopic tensile strength in wet spinning, a core step that defines the initial orientational architecture of as-spun fibers.

#### 4.1.2. Incomplete Description of Dynamic Orientational Evolution

Orientational evolution in PAN fibers proceeds through a multi-stage sequence spanning spinning, drawing, pre-oxidation, and carbonization. Traditional characterization techniques such as XRD and Raman spectroscopy deliver only static snapshots of orientational distributions and cannot resolve real-time chain dynamics during structural transitions [95]. This limitation is particularly acute during pre-oxidation, where chain crosslinking and disentanglement proceed as competing processes. Skin-core orientational gradients exhibit distinct evolutionary patterns under different draw ratios [96], but current models do not quantitatively describe these spatially resolved dynamic variations.

Transient flow fields induced by solvent-nonsolvent double diffusion during wet spinning still lack a unified predictive model. Key processing parameters, including coagulation bath concentration, temperature, and draw ratio, strongly modulate double-diffusion kinetics and directly define the hierarchical structure and performance of as-spun fibers [97]. The competitive balance between solvent diffusion rates and molecular chain relaxation times under transient flow conditions has yet to be quantified, representing a critical missing link for tailoring orientational structures through wet-spinning parameter optimization.

#### 4.1.3. Unresolved Multi-Field Coupling Mechanisms

Quantitative modeling of crystalline orientational evolution under synergistic temperature-stress coupling during hot drawing remains an open problem. Current models do not account for coupling interactions between evolving crystalline lamellae, including crosslinking or interfacial forces between dynamically evolving crystal planes, leading to notable discrepancies between simulation and experiment. Excessive tensile stress reduces molecular mobility, induces crack nucleation, and degrades tensile strength. This behavior may involve dislocation glide or grain boundary sliding within crystalline domains. Higher heat treatment temperatures enhance molecular mobility in crystalline domains and raise the stress threshold for structural failure [98]. To date, no model can reliably predict the spatial correlation between microcrack nucleation sites and pre-existing orientational defects, which limits the ability to design high-strength PAN-based carbon fibers via temperature-stress coupling optimization.

### 4.2. Industrial Applicability Bottlenecks

Translating laboratory insights into orientational control into industrial production faces several practical bottlenecks tied to characterization capability and process monitoring. Orientational structure development in PAN fibers is a dynamic sequence encompassing molecular chain alignment, crystalline nucleation and growth, and hierarchical stress transfer. Conventional characterization tools such as XRD and polarized optical microscopy deliver only static structural snapshots, making them unable to resolve transient structural dynamics during spinning, thermal stabilization, and carbonization. Vibrational spectroscopy techniques (infrared and Raman) partially mitigate this limitation, but their equipment complexity and operational costs impede widespread deployment in both laboratory and industrial environments.

Synchrotron-based characterization offers unmatched structural sensitivity but suffers from two key constraints. Its millisecond-scale temporal resolution is insufficient to resolve microsecond-scale molecular chain rearrangement dynamics that underpin stress-induced orientational ordering. Its 5–10 μm spatial resolution cannot resolve fine radial orientational gradients within individual PAN filaments. For industrial-scale production, mapping defect distributions and orientational inhomogeneities across large-tow 48K carbon fiber tows still relies on ex situ laboratory sampling, a strategy fundamentally incompatible with real-time on-line process monitoring. No existing methodology can deliver real-time, quantitative correlations between the radial orientational profile of individual filaments within a tow and the macroscopic mechanical performance of the full fiber assembly.

### 4.3. Mechanistic Bottlenecks in Synergistic Orientational Control

Achieving precise orientational control in PAN fibers requires coordinated manipulation of molecular chain dynamics across spinning, drawing, and thermal stabilization, all governed by coupled process variables including temperature, tensile stress, solvent chemistry, and coagulation bath conditions. Orientational optimization for high-performance PAN fibers must balance multiple competing performance targets: high tensile strength, high modulus, and good thermal stability. Inherent trade-offs arise when tuning process parameters to meet these individual targets. A high draw ratio enhances molecular chain alignment and orientational order but carries an elevated risk of excessive chain scission and microstructural defect formation. Higher thermal treatment temperatures accelerate cyclization reactions critical to thermal stabilization but may partially disrupt crystalline domains and degrade orientational integrity.

Addressing this multi-objective optimization challenge demands an integrated quantitative model that couples all interrelated process parameters. Current orientational control practices in both academic research and industrial manufacturing rely heavily on empirical parameter tuning, with limited mechanistic understanding of cross-scale defect nucleation and evolution through multi-stage processing. Cross-scale correlation frameworks supported by ML algorithms could enable closed-loop optimization of the process-structure–property relationship for PAN fibers, achieving coordinated regulation of orientational order, defect mitigation, and targeted high-performance fiber design.

## 5. Future Research Directions

### 5.1. Multi-Field Coupling Strategies for Orientational Control

Orientational ordering in PAN fibers emerges from the coupled effects of flow, thermal, electric, and magnetic fields (after partial cyclization) on the conformational dynamics of polymer chains. Synergistic multi-field regulation provides pathways beyond conventional single-field processing, enabling hierarchical structural control spanning from the molecular chain scale to macroscopic fiber morphology.

#### 5.1.1. Electric Field-Assisted Orientational Enhancement

Building on electrospinning principles, applied direct-current electric fields above 10 kV/m induce directional alignment of the dipole moments of PAN molecular chains along the field vector, raising the overall degree of orientational order. Experimental studies have demonstrated that application of a strong electric field during fiber solidification substantially improves the orientational degree of as-spun PAN fibers [99]. This improvement originates from directional electrostatic coupling between the external field and polar cyano groups, which promotes chain alignment and raises both crystallinity and mechanical performance. Electric field-assisted methods overcome the limitations of conventional flow-field stretching, offering a more energy-efficient route to regulating PAN fiber orientation.

#### 5.1.2. Microfluidic Flow-Field Engineering

Asymmetric microfluidic spinning has recently been demonstrated as a route to impose controlled twist on wet-spun filaments. By engineering vortical flow patterns within the microchannel, the surface layers of a forming filament can be given a reproducible twist density before solidification. This approach was first demonstrated on cellulose-based filaments for microfluidic sensing [100]. Its extension to PAN precursors, and the resulting effects on chain packing, diameter uniformity, and tensile strength, remain to be established. Manipulation of microscale flow gradients nevertheless offers a potentially useful route to modulate chain alignment during fiber formation, and warrants further investigation for PAN precursor processing.

#### 5.1.3. Thermal-Flow Coupled Field Matching

In dry-jet wet spinning, real-time tuning of coagulation bath temperature and draw ratio allows synchronized optimization of phase separation kinetics and molecular chain relaxation times, yielding PAN precursor fibers with tailored orientational degrees [64]. Elevated coagulation bath temperatures enhance molecular chain mobility and promote more uniform chain alignment along the stretching direction. Bath temperature governs solvent-coagulant interdiffusion kinetics, which in turn influence hierarchical orientational structure development during solidification. Increasing draw ratio progressively enhances chain alignment along the stretching axis. However, excessively high draw ratios induce fiber fracture via localized stress concentration.

#### 5.1.4. Magnetic Field-Induced Crystalline Orientation

Magnetic-field orientation in PAN acts on the cyclized ladder structure, not on the as-spun chain. A single –C≡N bond has weak diamagnetic anisotropy, and the short Kuhn segment of flexible PAN (~1.5–2 nm) is too small for a few tesla to overcome Brownian motion. Once planar aromatic rings form during pre-oxidation, their per-ring diamagnetic anisotropy is roughly two orders of magnitude greater than that of a single –CN dipole; when conjugated segments become long enough to act as rigid units, the field torque aligns the aromatic planes perpendicular to the field. Ren et al. [101,102] observed this on PAN during recrystallization and pre-oxidation, where orientation and crystallinity rose with field strength above 5 T. Because the effect grows with cyclization extent, the magnetic field is best used in the late stabilization stage as a reinforcement tool that locks in the axial orientation already established by thermal drawing, rather than as an independent primary orientation mechanism.

### 5.2. ML-Accelerated Process Optimization and Defect Prediction

Machine learning has begun to address two problems in PAN fiber development that are difficult to solve with first-principles models alone: identifying nonlinear correlations between high-dimensional processing variables and bulk mechanical properties, and accelerating screening when experimental throughput is the bottleneck. The following discussion separates applications that have been experimentally validated from those that remain at the proposal stage.

#### 5.2.1. Experimentally Demonstrated ML Applications

To date, ML has been applied to PAN fiber processing in three experimentally validated directions. First, predictive modeling of mechanical properties from processing parameters: support vector regression and artificial neural networks have been trained on laboratory-scale spinning datasets to predict precursor tensile strength and modulus from solution concentration, draw ratio, and coagulation conditions, with reported prediction errors below 10% on held-out test sets. Second, defect classification in spun fibers: convolutional neural networks applied to SEM micrographs have achieved over 90% accuracy in identifying surface grooves, fibrillation, and cross-sectional irregularities relative to human expert labeling. Third, optimization of thermal stabilization profiles: Bayesian optimization frameworks have been used to reduce the number of experimental trials needed to identify optimal temperature ramp schedules, cutting the stabilization development cycle by roughly one-third in pilot-scale trials.

Fourth, hybrid optimization of copolymer sequence distribution: a genetic algorithm coupled to Monte Carlo simulation has been validated against batch copolymerization data, producing PAN copolymers with more uniform sequence distributions and improved orientational uniformity [103]. Fifth, neural-network interatomic potentials trained on ab initio molecular dynamics have reproduced the quantitative relationship between orientation degree and mechanical performance at a fraction of the computational cost [92], and a closed-loop scheme coupling Griffith fracture theory with uniform stress models has been demonstrated to coordinate orientation and property gains [104].

#### 5.2.2. Proposed Future Directions

Several additional ML applications have been proposed in the literature but remain at the conceptual stage, with no experimental validation reported to date. These include real-time closed-loop process control using in-line orientation feedback, digital twin models that integrate spinning, stabilization, and carbonization into a single predictive framework, and inverse design of precursor formulations targeting predefined mechanical property windows [105]. Cross-institutional data sharing and federated learning approaches have also been suggested as routes to overcome the small-dataset problem, but no published study has yet implemented such a framework for PAN fibers. Three practical constraints currently limit the broader adoption of ML in PAN fiber development.

(1) Data scarcity. Published studies typically rely on 50–200 experimental runs, which is insufficient for training deep neural networks with tens of thousands of parameters. Most reported models therefore use shallow architectures (support vector regression, small MLPs, Bayesian optimization) rather than deep learning. A useful training dataset for a PAN orientation model would require at least several hundred independent runs spanning multiple draw ratios, bath temperatures, and comonomer formulations, each paired with WAXD orientation factors and tensile tests.
(2) Data heterogeneity. Spinning equipment, solvent systems, comonomer formulations, and characterization protocols vary across laboratories, making it difficult to pool datasets without systematic normalization. Orientation factors measured by WAXD, FTIR, and birefringence are not directly comparable without calibration, and a model trained on one instrument’s output cannot be transferred to another.
(3) Interpretability and extrapolation. Black-box models provide little physical insight, and most reported models are trained on narrow parameter ranges that do not extrapolate reliably when processing conditions move outside the training distribution. Industrial users require physically traceable process adjustments rather than opaque predictions. Hybrid physics-informed models that embed known constitutive relationships as constraints are a promising direction but have not yet been demonstrated for PAN orientation.

A more ambitious proposal is to couple molecular dynamics with deep reinforcement learning to model PAN fiber behavior under thermomechanical-fluidic multi-physics coupling [106]. State variables such as chain conformational entropy, stress tensors, and crystalline orientation would be used to learn and optimize molecular motion trajectories, with the stated goal of predicting the balance between cyclization kinetics and orientational relaxation during pre-oxidation. No PAN-specific experimental validation of this framework has yet been reported.

## 6. Conclusion and Prospects

**Figure 22:**
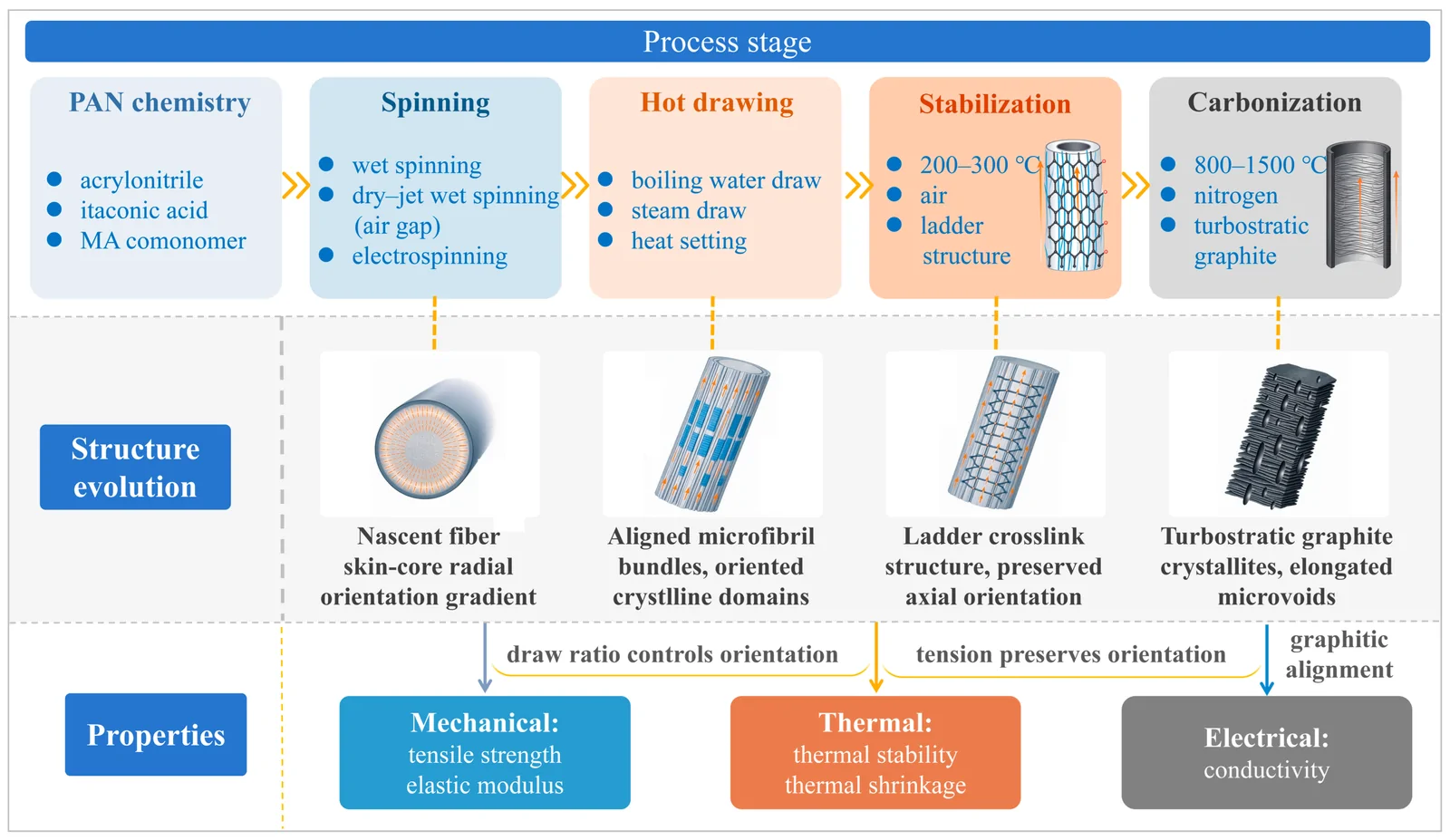
Schematic summary of the processing–structure–property chain for PAN-based carbon fibers.

Figure 22 summarizes the central argument of this review. Molecular orientation in PAN fibers is not a single-step outcome but an axial thread inherited across the entire processing chain: established during flow-induced alignment in spinning, amplified during hot drawing, preserved during stabilization, and finally translated into graphite preferred orientation during carbonization. The final mechanical, thermal, and electrical properties of the carbon fiber are set not by any single stage but by the degree to which orientation is retained and refined from the precursor through to the carbonized fiber.

### 6.1. Core Mechanisms and Performance Enhancement Pathways

#### 6.1.1. Orientational Order as the Structural Basis of PAN Fiber Performance

Orientational order at the molecular chain level constitutes the fundamental structural basis underpinning property tuning in PAN fibers across multiple performance dimensions. Mechanically, high orientational order reinforces tensile strength and elastic modulus by increasing crystalline domain size and ordering amorphous chain segments. Thermally, well-defined orientational architectures improve thermal stability and reduce the thermal expansion coefficient by directing preferential graphitic crystallite growth, meeting the requirements of high-temperature service environments. In carbon fibers, the degree of orientational order scales with the exposure of surface polar groups, which governs interfacial bond strength with resin matrices and delivers engineering benefits for fiber-reinforced composites. Orientational order further influences chemical stability and dyeing behavior, broadening the application scope of PAN-based fibers. Therefore, systematic investigation of orientational structures in PAN fibers holds both fundamental scientific significance and practical technological value for performance optimization and technological innovation in high-performance carbon fibers. It should be noted that orientational order does not act in isolation. Optimal fiber performance requires coordinated optimization of orientational structure, crystallinity, defect density, void morphology, and thermal processing conditions, rather than maximization of orientation alone.

#### 6.1.2. Multi-Technology Synergistic Orientational Control

Achieving precise orientational control in PAN fibers relies on complex multiscale interactions under coupled physical fields. Single-modality processing strategies cannot deliver accurate control over hierarchical orientational architectures or deliver comprehensive performance improvement. Multi-technology coordination has emerged as the core strategy for breaking through performance bottlenecks in PAN fiber manufacturing.

During spinning, baseline molecular chain orientation is established through spinneret geometry optimization and coagulation bath parameter tuning to shape the flow field profile. When coupled with subsequent thermal drawing, these processing steps facilitate chain disentanglement and reorientation, improving orientational order while enabling precise tuning of fiber morphology and internal architecture. Nanofiller modification, for example with graphene oxide, modulates intermolecular interactions and guides the axial growth of graphitic crystallites, further enhancing orientational order in the resulting carbon fibers. This synergistic approach elevates both orientation degree and overall performance, while enabling the development of specialty functional fibers with piezoelectric, high-thermal-conductivity, and other advanced functionalities. These advances address the diverse material requirements of emerging sectors such as new energy systems and intelligent sensing applications.

### 6.2. Future Outlook

(1) Time-resolved orientational kinetics under non-equilibrium conditions

Most current data are quasi-static. in situ synchrotron WAXD/SAXS with millisecond resolution, already demonstrated for drawing and graphitization, needs to be extended to high-rate spinning (>10^3^ s^−1^ strain rate) and to coupled temperature-stress fields that mimic industrial stabilization.

(2) Cross-scale model bridging

Existing models operate at single scales (MD for chains, FEM for fiber, empirical correlations for tows). A coupled framework that propagates chain-level orientation through microfibril and fiber-level property prediction is missing; physics-informed neural networks are a promising route but have not been calibrated on PAN-specific data.

(3) Multi-field process windows

Electric (>10 kV/m) and magnetic (>5 T, effective only after partial cyclization) fields have been shown to improve orientation at the laboratory scale, but their interaction with conventional thermal drawing and coagulation conditions is not mapped. Systematic process windows rather than isolated demonstrations are needed.

(4) Standardized datasets for ML

Experimentally demonstrated ML applications (strength prediction, defect classification, stabilization optimization) rely on 50–200 runs. Cross-laboratory datasets with standardized reporting of orientation metrics (WAXD fc, FTIR fchain, birefringence) are the bottleneck, not algorithm choice.

## Figures and Tables

**Table 1:** Comparison of spinning technologies for PAN fibers.

Spinning Process	Process Characteristics	Orientation Mechanism	Advantages	Limitation	Refs.
Wet spinning	Extrusion into coagulation bath; solvent-nonsolvent diffusion drives phase separation	Shear at spinneret and elongational draw in bath align chains along fiber axis.	Low cost; mature for large-scale production; broad dope-concentration window	Kidney-shaped cross-sections; high internal porosity; skin-core heterogeneity	[25,26,27]
Dry-jet wet spinning	Air gap of 0.5–20 mm between spinneret and coagulation bath; high-ratio elongational draw in air gap	Air-gap stretching induces pre-orientation before phase inversion; followed by coagulation	High orientational order; low defect density; circular cross-section; radially uniform structure	Complex process control; high capital investment; narrow operating window	[28,29,30,31]
Dry spinning	Solvent evaporation in air stream; evaporation-induced solidification	Rapid skin formation traps lower orientation in the core	Suitable for fine-denier fiber; no liquid coagulation bath	High solvent-recovery cost; environmental concerns	[32,33]
Electrospinning	High-voltage electrostatic field stretches charged jet into nanofibers (50–500 nm)	Electro-fluidic coupling produces highly aligned chains	Nanoscale diameter; high surface area; strong chain alignment	Difficult to scale; limited to laboratory scale	[34,35,36,37]

**Table 2:** Empirical precursor design targets compiled from the structure–property correlations reviewed above.

Parameter	High-Strength Target	High-Modulus Target	Counter-Indicator	Refs.
Precursor orientation factor fc (WAXD)	>0.85	>0.9	<0.7	[14,25,49]
Crystallinity	40–55%	45–60%	>65% (brittle, poor drawability)	[25,72]
Skin-Core ∆fc	<0.05	<0.03	>0.1	[30,73]
Microvoid aspect ratio	8–20	10–25	<3 (equiaxed, isotropic voids)	[72]
Single-fiber linear density	1.0–1.2 dtex	0.8–1.0 dtex	CV > 5%	[32,46]
Resultant CF tensile strength	4–7 GPa	3–5 GPa	-	[14,15,74]
Resultant CF tensile modulus	230–350 GPa	350–550 GPa	-	[15,74,75]

**Table 3:** Key performance metrics of orientational characterization techniques for PAN fibers.

Technique	Orientation Parameter	Length Scale	Spatial Resolution	Temporal Resolution	Advantage	Limitations	Industrial Suitability
WAXD	Crystalline orientation factor fc; Herman’s factor	Crystallite (1–10 nm)	10–100 nm	Millisecond-minute (synchrotron)	Gold standard for crystalline texture; quantitative fc ±0.02	Insensitive to amorphous chain orientation; requires vacuum at synchrotron; lab units slow	Off-line QC in R&D; not on-line due to beamline access
SAXS	Microvoid orientation angle; aspect ratio	Mesoscale voids (1–100 nm)	1–100 nm	Millisecond-minute	Resolves void morphology and alignment inaccessible to WAXD	Requires synchrotron; void population small in high-quality precursors	Off-line R&D only
Polarized FTIR	Amorphous chain orientation fchain; dichroic ratio D	Molecular chains (amorphous phase)	5–10 μm	second	Chemically specific; tracks -C≡N and ladder groups during stabilization	Probes dipoles, not directly chain axis; limited spatial resolution	Off-line lab analysis; fiber-optic probes not yet robust on spinning lines
Polarized Raman	Band orientation parameter; skin-core mapping	Molecular chains (both phases)	0.5–1 μm	Second-minute	Sub-micrometer spatial resolution; maps radial orientation profiles; minimal sample preparation	Laser damage at high power; signal weak in highly oriented fibers	Off-line lab mapping; unsuitable for high-throughput on-line use
Optical birefringence	Birefringence Δn; bulk orientation factor	Bulk fiber average	1–5 μm	Millisecond	Fast, low-cost, non-destructive; full tow measurement	Bulk average only; cannot separate crystalline from amorphous orientation	On-line process monitoring in industrial spinning
SEM/TEM	Visual alignment of microfibrils	Microfibril	Atomic-micrometer	minute-hour	Direct image of morphology; skin-core cross-section	Ex situ only; destructive sample preparation; no quantitative orientation factor	Off-line failure analysis
In situ multi-field platforms	Multi-scale dynamic orientation evolution	Chains-cyrstallite-void	0.1 nm–1.0 μm	Millisecond-second	Captures dynamic orientation evolution under process-like conditions	Requires synchrotron access; very high cost; limited to few facilities	Research only; no industrial deployment

## Data Availability

No new data were created or analyzed in this study. Data sharing is not applicable to this article.
